# Absolute configuration and host-guest binding of chiral porphyrin-cages by a combined chiroptical and theoretical approach

**DOI:** 10.1038/s41467-020-18596-1

**Published:** 2020-09-22

**Authors:** Jiangkun Ouyang, Anne Swartjes, Marc Geerts, Pieter J. Gilissen, Danni Wang, Paula C. P. Teeuwen, Paul Tinnemans, Nicolas Vanthuyne, Sara Chentouf, Floris P. J. T. Rutjes, Jean-Valère Naubron, Jeanne Crassous, Johannes A. A. W. Elemans, Roeland J. M. Nolte

**Affiliations:** 1 Radboud University, Institute for Molecules and Materials, Heyendaalseweg 135, 6525 AJ Nijmegen, The Netherlands; 2 Aix Marseille Univ, CNRS, Centrale Marseille, iSm2, Marseille, France; 3 FSCM - Spectropole, FR1739, Aix-Marseille University, CNRS, Centrale Marseille, 13397 Cedex 20 Marseille, France; 4 Univ Rennes, CNRS, Institut des Sciences Chimiques de Rennes, ISCR-UMR 6226, F-35000 Rennes, France

**Keywords:** Stereochemistry, Molecular capsules

## Abstract

Porphyrin cage-compounds are used as biomimetic models and substrate-selective catalysts in supramolecular chemistry. In this work we present the resolution of planar-chiral porphyrin cages and the determination of their absolute configuration by vibrational circular dichroism in combination with density functional theory calculations. The chiral porphyrin-cages form complexes with achiral and chiral viologen-guests and upon binding one of the axial enantiomorphs of the guest is bound selectively, as is indicated by induced-electronic-dichroism-spectra in combination with calculations. This host-guest binding also leads to unusual enhanced vibrational circular dichroism, which is the result of a combination of phenomena, such as rigidification of the host and guest structures, charge transfer, and coupling of specific vibration modes of the host and guest. The results offer insights in how the porphyrin cage-compounds may be used to construct a future molecular Turing machine that can write chiral information onto polymer chains.

## Abstract (web summary)

Vibrational circular dichroism (VCD) spectroscopy can be useful for determining the absolute configuration of chiral molecules, as long as the signal intensities are high enough. Here, the authors establish the absolute configurations of two large chiral porphyrin cages and, notably, discover that host-guest binding enhances their VCD intensities.

## Introduction

In organic chemistry the determination of the absolute configuration of a chiral molecule is often a problem that is difficult to solve, particularly when X-ray structures of the enantiopure compounds are not available^1,2^. When possible, one may resort to vibrational and electronic circular dichroism spectroscopies in combination with calculations. Circular dichroism (CD) is the difference in response of a chiral molecule to left- and right-circularly polarized radiation, which may relate to the infrared (VCD) or to the UV-vis (ECD) spectral region. A comparison of the frequencies, signs, and intensities of experimental VCD spectra with those calculated by density functional theory (DFT) for a chosen configuration of a chiral molecule can unambiguously capture its absolute configuration, at least in principle^2,3^. For a more reliable result, the analysis can be completed by following the same approach using ECD and time-dependent density functional theory (TD-DFT) calculations, provided the molecule contains a chromophore. Although VCD has a great advantage over ECD, as it requires no chromophores, it often suffers from lower signal intensities. Because of this the experiments have to be performed with highly concentrated samples (Δ*ε* typically scales as 10^−4^–10^−3^ *ε*)^3^. It is of great interest, therefore, to find processes that can enhance VCD intensities^4–7^ and in the past decades chemists have been able to achieve this by different methods including manipulation of the electronic manifold^8,9^, forming metal complexes^8,10^, chiral crystal packing^11^, and fibril formation^12–14^. Until now there are no examples of VCD enhancement by supramolecular interactions, i.e., by forming host–guest complexes^15,16^.

**Fig. 1:**
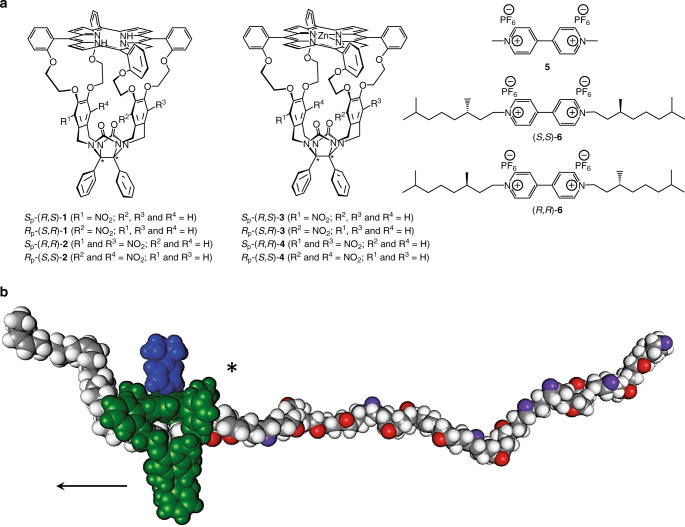
Structure of compounds and writing of chemical information. **a** Molecular structures of hosts and guests: chiral porphyrin-cages **1**-**4** and achiral and chiral viologen guests **5** and **6**. **b** Schematic representation of a chiral manganese porphyrin cage catalyst (green) provided with a 4-*tert*-butylpyridine ligand (blue) gliding along a chiral polymer chain (e.g. polybutadiene), while converting the polymer double bonds into chiral epoxide functions (red and purple), see text.

Herein we report the efficient and straightforward resolution of two planar chiral porphyrin-cages (**1** and **2**, see Fig. 1a) by chiral HPLC and the determination of their absolute configurations by vibrational^3^ and electronic circular dichroism^17^ in combination with DFT and TD-DFT calculations. These absolute configurations have been checked by X-ray diffraction, which confirmed the assignments made by the combined spectroscopic-theoretical analysis. The molecular sizes of the studied compounds are larger than those of any other published compound to date for which VCD has been applied to assign absolute configurations. Furthermore, we present induced circular dichroism (ICD) experiments showing that the chiral porphyrin cage molecules display enantioselectivity in the binding of both achiral and chiral *N*,*N*-substituted 4,4′-bipyridinium (viologen) guest molecules and that on binding one of the interconverting axial enantiomorphs of the guest is preferred. Remarkably, certain combinations of chiral host and guest complexes display amplified VCD spectra, constituting the first example of VCD enhancement in a host–guest system. The work presented here is part of a larger project aimed at encoding information into single polymer chains with the help of catalytic molecular machines (Turing machines)^18^ that write digital data in the form of chemical functions (i.e. chiral epoxides: (*R*,*R*)-epoxide = digit 1, (*S*,*S*)-epoxide = digit 0, Fig. 1b), while gliding along these chains. Chiral cage compounds **1** and **2** provided with a catalytically active metal (e.g. manganese) and light-switchable chiral functions on their cavity walls, are conceived to be used for that purpose^19–21^.

## Results

### Resolution and assignment of chirality

**Fig. 2:**
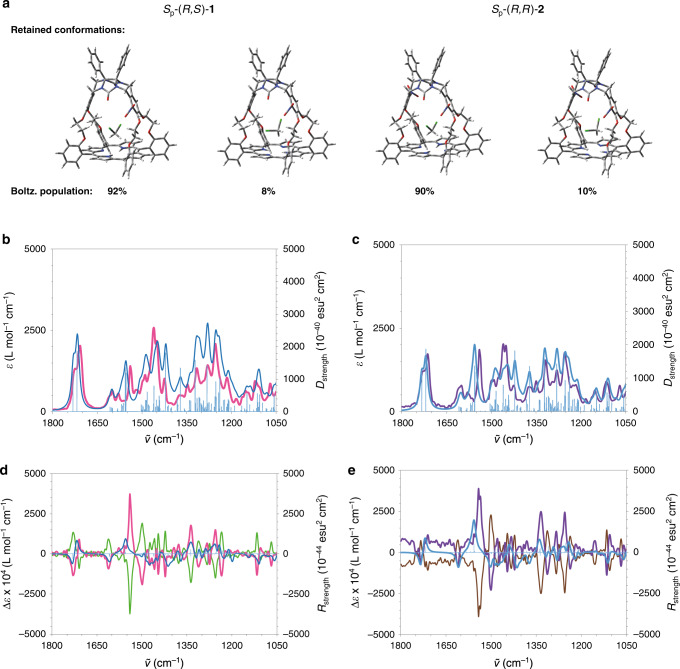
Experimental and calculated IR and VCD spectra. **a** Calculated conformations and Boltzmann populations of *S*_p_-(*R*,*S*)-**1** and *S*_p_-(*R*,*R*)-**2**. **b** IR spectra measured for (+)−**1** (magenta line) and calculated for *S*_p_-(*R*,*S*)-**1** (blue line and blue bars: dipole strengths). **c** IR spectra measured for (+)−**2** (purple line) and calculated for *S*_p_-(*R*,*R*)-**2** (blue and blue bars: dipole strengths). **d** VCD spectra measured for (‒)−**1** (green line), (+)−**1** (magenta line) and calculated for *S*_p_-(*R*,*S*)-**1** (blue line and blue bars: rotational strengths). **e** VCD spectra measured for (‒)–**2** (brown line), (+)−**2** (purple line) and calculated for *S*_p_-(*R*,*R*)-**2** (blue line and blue bars: rotational strengths). Solvent CD_2_Cl_2_, *c* = 1 × 10^−1^ M.

Compounds **1** and **2** contain one and two nitro-functions, respectively, and were prepared from the parent porphyrin cage compound (R^1^, R^2^, R^3^, R^4^ = H) via our previously reported highly selective nitration reaction^22^. The introduction of these nitro-substituents on the side walls of the porphyrin cage provides planar chirality as well as point chirality to the compounds (Supplementary Fig. 1). The resolution of the resulting racemic mixtures was achieved by chiral HPLC (Supplementary Information) and the two enantiomers of **1**, i.e. *S*_p_-(*R*,*S*)-**1** and *R*_p_-(*S*,*R*)-**1**, and those of **2**, i.e. *S*_p_-(*R*,*R*)-**2** and *R*_p_-(*S*,*S*)-**2** were obtained in excellent enantiomeric excesses (ee >99.5%) and turned out to be thermally stable (see Supplementary Figs. 24 and 25). To elucidate the absolute configurations of the porphyrin cages, VCD spectra were recorded and DFT calculations performed on the pure enantiomers of the two cage compounds. In a first approach, the calculations were carried out with the implicit solvent model SMD^23^. The functional B3LYP associated with basis set function 6-311G(d) and the dispersion potential GD3Bj were used for the calculations of the IR and VCD spectra^24^. The results turned out to be unsatisfactory and we improved our model by introducing one explicit solvent molecule in the host molecule, which position was determined with the help of molecular dynamics calculations (see Supplementary Information for details). Although only one conformation of the host with one explicit solvent molecule was taken into account, this model significantly improved the agreement between the measured and calculated spectra. The experimental IR and VCD spectra of (−)**−1** and (+)**−1** were recorded in the region from 1825 to 1025 cm^−1^ ((−) stands for the first eluted fraction showing a negative sign in the CD spectrum at 254 nm and (+) for the second eluted fraction showing a positive sign in the CD spectrum at 254 nm). The experimental and calculated IR spectra of this compound were found to be in excellent agreement (Fig. 2). Furthermore, the calculated VCD spectrum of *S*_p_-(*R*,*S*)**−1** corresponded well with the experimental VCD spectrum of the second fraction (+)**−1**, revealing that the compound in this fraction had the absolute configuration *S*_p_-(*R*,*S*). Based on this result we can assign the *R*_p_-(*S*,*R)*-configuration to the first eluted fraction, i.e. (−)**−1**. The IR and VCD spectra of the enantiomers of the anti-dinitro porphyrin-cages **2** were also calculated and compared with the experimental ones to determine their absolute configurations (Fig. 2). In this way we could assign the *R*_p_-(*S*,*S*)-configuration to the first eluted enantiomer (−)−**2**, and *S*_p_-(*R*,*R*) configuration to the second eluted one, (+)−**2**. The VCD spectra of the enantiomers of the nitro-porphyrin-cages **1** and **2** displayed strong similarities: the carbonyl stretching modes exhibited a couplet that results from a mixing of the two stretching C = O modes. Between 1250 and 1350 cm^−1^, the same succession of three negative and positive bands was a reliable fingerprint of the cavity of the host compound. The vibrational modes contributing to these bands are not dominated by vibrations of one functional group, but a mixing of stretching (C–O, C = C, and C–N) and/or deformations (C–H, N-H bending, and CH_2_ twisting) involving atoms of the whole molecule. The most intense VCD band was located at 1541 cm^−1^ (Fig. 2) and corresponds to a complex vibrational mode that mixes the NO_2_ stretching vibration with the C = C_arom._ and C–H deformations of the nitro-xylylene moieties. The VCD spectra furthermore revealed that several of the VCD band intensities of (−)**−2** and (+)**−2** were higher than those of (−)**−1** and (+)**−1** (Fig. 2), which may result from the fact that **2** has a C_2_ symmetry axis, leading to a double planar chirality.

**Fig. 3:**
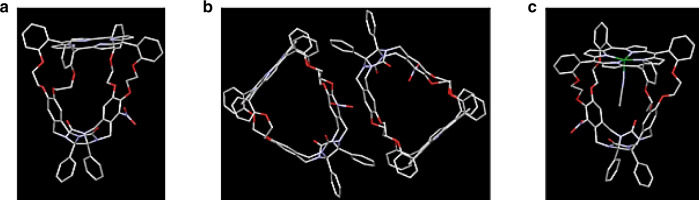
X-ray structures of enantiopure cage compounds. **a** X-Ray structure of (−)-*R*_p_-(*S*,*R*)-**1**. **b** View showing the arrangement of the enantiomeric molecules in the crystal of (−)-*R*_p_-(*S*,*R*)-**1**. **c** X-Ray structure of (+)-*S*_p_**-**(*R*,*S*)-**3** with an acetonitrile molecule inside the cage. Color codes: oxygen, red; nitrogen, purple; carbon, gray; and zinc, green. Hydrogen atoms have been omitted for clarity.

To confirm the assignments of the absolute configurations made by VCD and DFT we decided to grow single crystals from the enantiomers of the cage compounds and perform X-ray analyses. This turned out to be possible for (−)**−1** (Fig. 3a, b), but not for an enantiomer of **2**. The space group of (−)**−1** was non-centrosymmetric P2_1_2_1_2_1_, in line with the chiral nature of the species, and the crystal structure unambiguously proved that (−)**−1** has the absolute configuration *R*_p_**-**(*S*,*R*). We also prepared the enantiopure zinc complexes (−)**−3**, (+)**−3**, (−)**−4**, and (+)**−4** from compounds **1** and **2** (Supplementary Information) and succeeded to grow single crystals from (+)**−3** suitable for X-ray analysis, but unfortunately not from one of the enantiomers of **4**. The crystal structure of (+)**−3** confirmed the absolute configuration assigned on the basis of VCD/DFT and the X-ray structure of (−)**−1** and furthermore revealed the coordination of an acetonitrile molecule to the zinc center inside the zinc (+)**−3** cage (Fig. 3c).

**Fig. 4:**
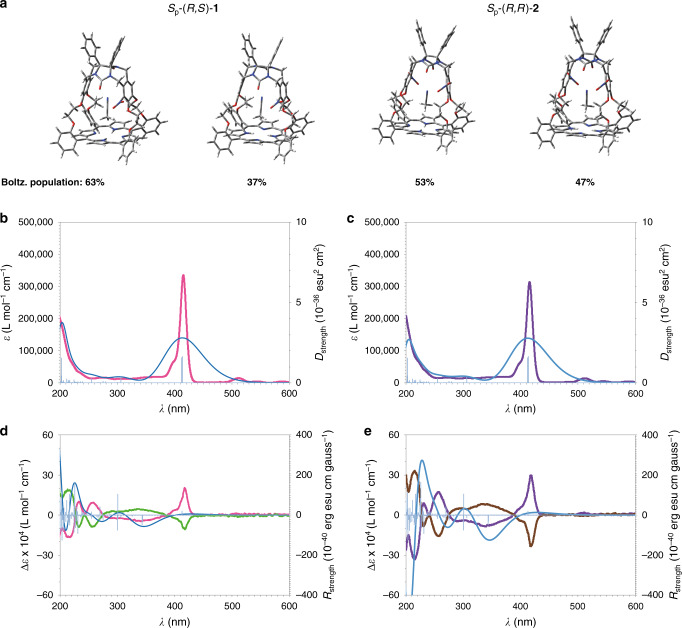
UV and ECD spectra of chiral porphyrin-cage compounds. **a** Calculated conformations and Boltzmann population of *S*_p_-(*R*,*S*)-**1** and *S*_p_-(*R*,*R*)-**2**. **b** UV-Vis spectra measured for (+)−**1** (magenta line) and calculated for *S*_p_-(*R*,*S*)-**1** (blue line and blue bars: dipole strengths). **c** UV-Vis spectra measured for (+)−**2** (purple line) and calculated for *S*_p_-(*R*,*R*)-**2** (blue line and blue bars: dipole strengths). **d** ECD spectra measured for (+)−**1** (magenta line), (*−*)**−1** (green line), and calculated for *S*_p_-(*R*,*S*)**-1** (blue line and blue bars: rotational strengths). **e** ECD spectra measured for (+)−**2** (purple line), (*−*)−**2** (brown line), and calculated for *S*_p_-(*R*,*R*)-**2** (blue line and blue bars: rotational strengths). Solvent CH_3_CN, *c* = 5.55 × 10^−5^ M.

To obtain further insight into the properties of enantiopure **1** and **2**, the ECD spectra of the separated enantiomers were recorded in acetonitrile in the region 190–600 nm (Fig. 4). Each pair of enantiomers displayed mirror-image spectra within experimental errors (see also Supplementary Information, Table 1 and Fig. 27). For *R*_p_-(*S*,*R*)**−1** a positive Cotton effect (CE)^25^ is visible in the region from 250 nm to 320 nm (*λ*_317_ nm: *∆ε* + 6.1 M^−1^ cm^−1^; *λ*_255_ nm: *∆ε−*19.2 M^−1^ cm^−1^; A-value +25.3 M^−1^ cm^−1^ linked to the ^1^L_b_ transition^26^, see Supplementary Fig. S27). As expected, *S*_p_-(*R*,*S*)**−1** displayed a negative CE with similar characteristics. The ECD spectra were calculated by TD-DFT using the LC-WhPBE functional with the def2SVP basis set and SMD for solvation effects (see Supplementary Information for details). As for VCD, better results were obtained with a model that takes into account the presence of one explicit solvent molecule. The calculated UV and ECD spectra of *S*_p_**-**(*R,S*)**−1** and *S*_p_-(*R,R*)**−2** showed a satisfying agreement with the measured spectra of (+)**−1** and (+)**−2**, respectively, unambiguously confirming the assignments of the absolute configurations made above (Fig. 4).

**Table 1:** Host–guest binding titrations.

	(*S*,*S*)-**6**		(*R*,*R*)-**6**	
	*K*_a_	Δ*G*^*o*^	*K*_a_	Δ*G*^*o*^
*R*_p_-(*S*,*R*)-**1**	262 (± 12) x 10^4^	−36.6	325 (± 19) x 10^4^	−37.1
*R*_p_-(*S*,*S*)-**2**	9.4 (± 0.2) x 10^4^	−28.3	10.6 (± 0.6) x 10^4^	−28.7

Association constants *K*_a_ (M^−1^) and binding free energies Δ*G*^*o*^ (kJ mol^−1^) of complexes between chiral hosts *R*_p_-(*S*,*R*)-**1** and *R*_p_-(*S*,*S*)-**2** and viologen guests (*S*,*S*)-**6** and (*R*,*R*)-**6**.

### Host–guest binding

**Fig. 5:**
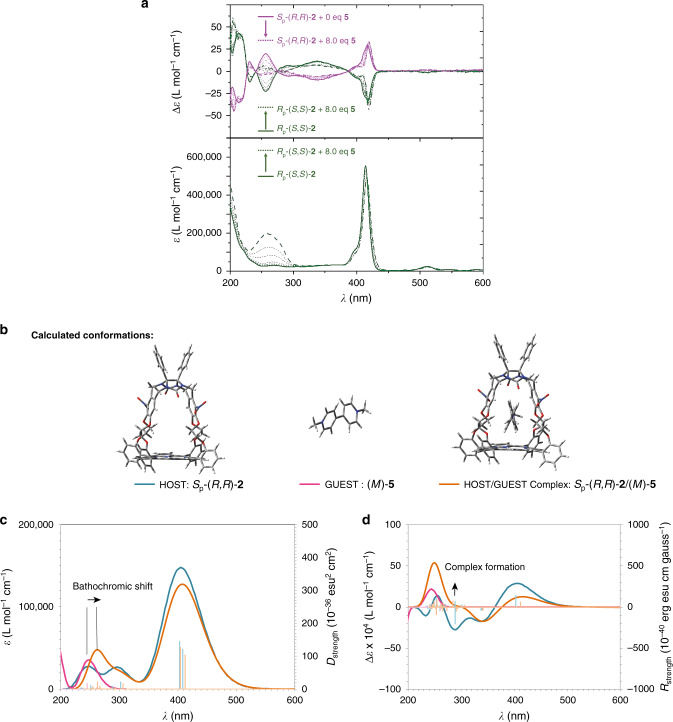
ECD and UV-vis spectra of host–guest complexes. **a** ECD and UV-vis spectra of *S*_p_-(*R*,*R*)-**2** and *R*_p_-(*S*,*S*)-**2** (solvent acetonitrile, *c* = 7.5 × 10^−5^ M) upon addition of increasing amounts of guest **5**. **b** Calculated conformations of host *S*_p_-(*R*,*R*)-**2**, the (*M*)-form of guest **5**, and the complex of this host and guest *S*_p_-(*R*,*R*)-**2**/(*M*)-**5 c**. Calculated UV-vis and **d** calculated ECD spectra of the retained conformations of the host *S*_p_-(*R*,*R*)-**2** (blue line and blue bars: dipole (UV) and rotational (ECD) strengths), of the guest *(M)-****5*** (pink line and pink bars: dipole (UV) and rotational (ECD) strength), and of the complex *S*_p_-(*R*,*R*)-**2**/(*M*)-**5** (orange line and orange bars: dipole (UV) and rotational (ECD) strengths).

Viologen guests can bind into the chiral porphyrin cages and form stable 1:1 host–guest complexes, which are held together by *π*−*π* and van der Waals interactions between the guest and the xylylene ring side walls of the host (vide infra)^27^. To investigate how the binding of viologen guests influences the chiroptical properties of the chiral porphyrin hosts, ECD spectra were recorded^28^. Since the CD signal of **2** is stronger than that of **1**, first experiments with the enantiomers of the former compound were performed. When the achiral guest methyl viologen **5** (*K*_a_ with **2** = 8.4 × 10^4^ M^−1^ in CHCl_3_/CH_3_CN 1:1 v/v) was added in increasing amounts to a solution of *S*_p_-(*R*,*R*)-**2** in acetonitrile, the intensity of the positive CD signal at 252 nm of the host decreased and then became negative (Fig. 5a). Unfortunately, the observed changes (induced CD, abbreviated ICD)^29–36^ at this wavelength coincide with the *π*−π^*^ transition of the guest, which has an absorption maximum in the same region. The addition of **5** to a solution of *R*_p_-(*S*,*S*)-**2** in acetonitrile resulted in an opposite ICD signal, as expected. In order to better understand the ICD phenomenon, we optimized one conformation of the complex formed by *S*_p_-(*R,R*)-**2** and **5** using DFT calculations. Two possibilities were considered: viologen **5** is bound in a ‘horizontal’ orientation (perpendicular to the xylylene sidewalls and parallel to the porphyrin ring) or in a ‘vertical’ orientation (parallel to the xylylene side walls and perpendicular to the porphyrin ring) in the cavity of the host. Attempts to optimize the complex *S*_p_-(*R*,*R*)-**2**/**5** starting from geometries in which the guest is in a horizontal position, always converged to a conformation in which guest **5** is oriented in a vertical position (Fig. 5b). This result is in line with previous NMR experiments^27^. In this vertical conformation, the guest is held in the host by a combination of electrostatic interactions, hydrogen bonding, *π*−*π*, CH−*π*, and van der Waals interactions. Interestingly, the chiral environment of the cavity of the host induces an asymmetry in the equilibrium between the two enantiomorphic (twisted) conformations of **5**: according to the calculations, only the left (*M*)-twisted guest can be hosted by the porphyrin cage of *S*_p_-(*R,R*)-**2** (Fig. 5b). The ICD phenomenon observed here is comparable to the one reported for the binding of a viologen derivative in cyclodextrin host molecules^37^. Using the (*M*)-axial conformation of the guest, we calculated the UV and ECD spectra of the host–guest complex using TD-DFT (Fig. 5c, d and Supplementary Information). The calculated UV spectra showed that the *π*−π* absorption band of the guest indeed is superimposed on a *π*−π* absorption band of the host. When the host–guest complex is formed, the *π*−π* absorption band of the guest shifts to lower energies (bathochromic shift) and appears in an area where the host absorbs less. For the (*M*)-**5** enantiomorph, the ECD band of the *π*−π* transition at 248 nm transition is positive. The bathochromic shift of the *π*−π* band in the complex superimposes it on the negative band at 264 nm of the host, thus canceling the signal, in agreement with the experiments (Fig. 5a). The calculations clearly show that the ICD phenomenon essentially results from the presence of only the (*M*)-enantiomorphic conformation of the guest **5** in the complex with host *S*_p_-(*R,R*)-**2**. In separate experiments we also added the chiral guests (*S*,*S*)-**6**, and (*R*,*R*)-**6** to hosts *S*_p_-(*R*,*R*)-**2** and *R*_p_-(*S*,*S*)-**2**. The ECD spectra showed that these guests displayed similar ICD behavior as observed for the achiral guest **5** (Supplementary Figs. 35 and 36). Finally, these guests were also used to form complexes with hosts *S*_p_-(*R*,*S*)**−1** and *R*_p_-(*S*,*R*)**−1** (Supplementary Figs. 38 and 39). The measured ICD trends were similar to the ones recorded for *S*_p_-(*R*,*R*)-**2** and *R*_p_-(*S*,*S*)-**2** and these guests, as expected from the theoretical analysis.

Fluorescence titration experiments were carried out in CHCl_3_/CH_3_CN (1:1, v/v) to investigate the binding of chiral viologen guests (*S*,*S*)-**6** and (*R*,*R*)-**6** to chiral hosts *R*_p_-(*S*,*R*)-**1** and *R*_p_-(*S*,*S*)-**2** (Supplementary Figs. 48–51). Upon complexation of the guests, the bands at 650 and 715 nm in the fluorescence spectra of the hosts decreased in intensity as a result of quenching. From the titration curves the association constants (*K*_a_) and the binding free energies Δ*G*^*o*^ of the complexes between *R*_p_-(*S*,*R*)-**1** and *R*_p_-(*S*,*S*)-**2**, and the two chiral guests (*S*,*S*)-**6** and (*R*,*R*)-**6** were calculated. The results are presented in Table 1. Compared to *R*_p_-(*S*,*S*)-**2**, the *K*_a_-values for the complexes between *R*_p_-(*S*,*R*)-**1** and the chiral guests (*S*,*S*)-**6** and (*R*,*R*)-**6** are significantly higher, which may result from the lower steric hindrance in the latter host as a result of the presence of only one nitro group. Furthermore, the binding constants of the complexes between (*R*,*R*)-**6** and *R*_p_-(*S*,*R*)-**1** and *R*_p_-(*S*,*S*)-**2** are somewhat higher than those of the complexes between (*S*,*S*)-**6** and *R*_p_-(*S*,*R*)-**1** and *R*_p_-(*S*,*S*)-**2**. Apparently, the chiral centers of (*R*,*R*)-**6** and (*S*,*S*)-**6** can still influence the association constants of the complexes with the chiral hosts *R*_p_-(*S*,*R*)-**1** and *R*_p_-(*S*,*S*)-**2**, despite the fact that they are quite remote from the location where the actual binding interactions occur.

**Fig. 6:**
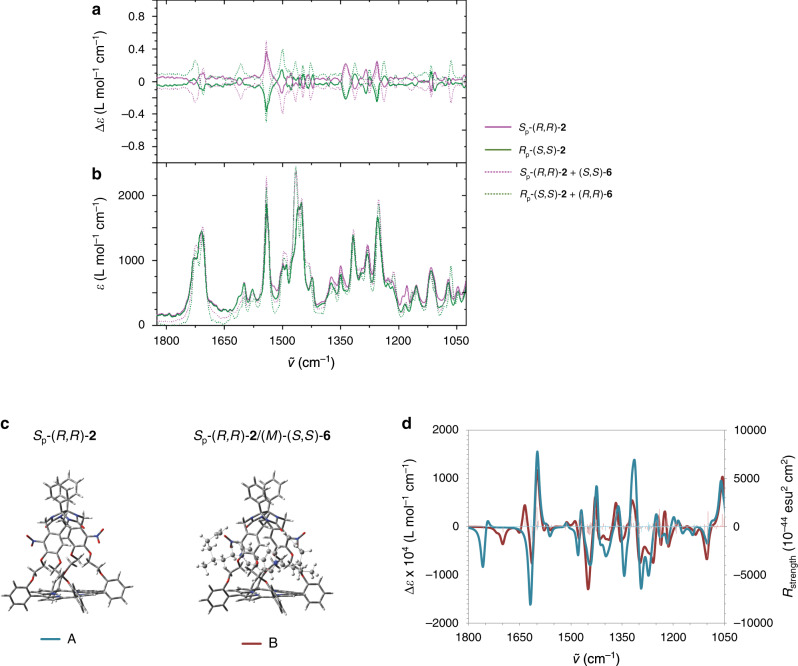
VCD spectra of chiral hosts and chiral host–guest complexes. **a** VCD spectra of *S*_p_-(*R*,*R*)-**2** (purple line) and *R*_p_-(*S*,*S*)-**2** (green line) and of the complexes between *S*_p_-(*R*,*R*)-**2** and (*S*,*S*)-**6** (purple dotted line), and *R*_p_-(*S*,*S*)-**2** and (*R*,*R*)-**6** (green dotted line). **b** IR spectra of these hosts and host–guest complexes. Solvent CDCl_3_. **c** Calculated conformation of the host *S*_p_-(*R*,*R*)-**2** (A) and of the complex *S*_p_-(*R*,*R*)-**2**/(*M*)-(*S*,*S*)-**6**. **d** Calculated VCD spectra of the retained conformation A of the host *S*_p_-(*R*,*R*)-**2** (blue line and blue bars: rotational strengths), and of the conformation B of the complex *S*_p_-(*R*,*R*)-**2**/(*M*)-(*S*,*S*)-**6** (red-brown line and red-brown bars: rotational strengths).

The complexation of the chiral guest can be expected to perturb the conformation of the chiral host and hence change its VCD spectrum, compared to that of the free host molecule. With the aim of understanding this point, the VCD spectra of a series of host–guest complexes were investigated. Since different chiral guests may bring about different perturbances, experiments with the following combinations of complexes were performed: *S*_p_-(*R*,*R*)-**2/**(*S*,*S*)-**6**, *S*_p_-(*R*,*R*)-**2/**(*R*,*R*)-**6**, *R*_p_-(*S*,*S*)-**2/**(*S*,*S*)-**6**, and *R*_p_-(*S*,*S*)-**2**/(*R*,*R*)-**6** (all in 1:1 molar ratios). The VCD and IR spectra were recorded in the 1825-1025 cm^−1^ region. Interestingly, in the VCD spectra, the signals of *S*_p_-(*R*,*R*)-**2/**(*S*,*S*)-**6**, and *R*_p_-(*S*,*S*)-**2**/(*R*,*R*)-**6** at 1064 cm^−1^, 1238 cm^−1^, 1499 cm^−1^, 1541 cm^−1^, and 1704 cm^−1^ were enhanced, i.e. by a factor 1.5–2, relative to that of the free chiral hosts (Fig. 6). To ensure that the enhanced signals were not originating from the free guest molecules, also the VCD spectra of (*R*,*R*)-**6** and (*S*,*S*)-**6** were recorded. (Supplementary Fig. 44). The VCD signals of these free guests were very weak compared to those of the free chiral hosts and we may conclude, therefore, that the observed enhanced signals must result from the formed host–guest complexes. In order to obtain more insight in this phenomenon, DFT calculations were performed. Due to the sizes of the host–guest complexes, these calculations were carried out on only one of the most stable host–guest conformations and the used theoretical level was slightly lowered (see Supplementary Information for details). Hence, we calculated the IR and VCD spectra of the complexes of host *S*_p_-(*R,R*)-**2** with the (*M*)-axial conformation of the guests (*R,R*)-**6**, (*S,S*)-**6** (see Supplementary Fig. 56). It should be noted that these calculated spectra for only one conformation of course are not sufficient to correctly model the experimental spectra and particularly the phenomenon of VCD bands amplification. Although incomplete, these calculations provide us with keys to understand what effects govern this phenomenon. The first effect is related to the formation of the host–guest complex, which rigidifies the structures of both the host and the guest by limiting their conformational space. This has a direct impact on the VCD spectrum, which globally becomes more intense. More specifically, inside the chiral host the axial rotation around the central C–C bond of the 4,4′-bipyridinium moiety of the guest is blocked, allowing only one axial conformation of the guest to be present (Fig. 6c). From the calculated structures we established that this phenomenon is associated with VCD bands between 1500 and 1650 cm^−1^. These bands, which are not present in the VCD spectra of the free guests, correspond to vibrational breathing modes of the 4,4′-bipyridinium moiety. Another effect that can significantly modify the VCD spectra is the mixing of vibrational modes. Indeed, calculations show that in the free host *S*_p_-(*R,R*)-**2** some vibrational modes are mixed, for instance the C = O stretching or the breathing mode of the xylylene rings. Insertion of a guest molecule into the cavity may prevent or modify this mixing, leading to significant changes (frequency shifts, intensity decreases, and, for VCD, change of the signs of bands) in the spectra. For instance, in the free host *S*_p_-(*R,R*)-**2** the breathing modes of the xylylene rings are mixed but, when guest (S,S)-**6** is inserted into the cage, such mixing no longer occurs and the intensity of the corresponding negative band at 1619 cm^−1^ decreases (Fig. 6d). Furthermore, calculations show that in the complex, vibrational modes of both host and guest are coupled. Such spatial coupling between vibrational modes of two molecules in close proximity is generally associated with enhanced VCD bands of which the magnitude may be several orders higher than those of the other bands in the spectrum. It is well-established that such coupling strongly depends on the relative orientations of the guest and the host^38,39^. The calculations revealed that in the complex *S*_p_-(*R,R*)-**2**/(*S,S*)-**6** a coupling between the bending vibrational modes of the CH_2_ and CH_3_ groups of the guest and of the CH_2_ groups of the ethyleneoxy linkers of the cage occurs. This coupling is associated with a significant enhancement of the negative band at 1450 cm^−1^ (Fig. 6d). Finally, the cationic nature of the viologen moiety of the guest may allow charge transfer with the host, resulting in large VCD bands^7,40^. In summary, it is reasonable to believe that the phenomenon of amplification of the VCD bands observed in the measured spectra of the complexes results from a combination of these five phenomena: (i) rigidification of the host and guest structures upon complexation, (ii) enantiomorphism of the guest inside the host, (iii) the loss or the modification of some mixing vibrational modes in the host upon guest complexation, (iv) spatial coupling of specific vibrational modes of the host and guest, and (v) charge transfer between the guest and the host.

For the other combinations of host and guest, i.e. *S*_p_-(*R*,*R*)-**2/**(*R*,*R*)-**6**, and *R*_p_-(*S*,*S*)-**2**/(*S*,*S*)-**6**, VCD enhancements (Supplementary Fig. 47) were observed as well, but the increases in intensity were not as strong as for the host–guest combinations of *S*_p_-(*R*,*R*)-**2/**(*S*,*S*)-**6** and *R*_p_-(*S*,*S*)-**2**/(*R*,*R*)-**6**. We speculate that the differences in the binding strengths of the host–guest complexes may cause these enhancement differences. Indeed, the *K*_a_-value of the complex between *R*_p_-(*S*,*S*)-**2** and (*R*,*R*)-**6** is higher than that of the complex between *R*_p_-(*S*,*S*)-**2** and (*S*,*S*)-**6** (see Table 1), which is in agreement with the observed VCD results. The host–guest complexes of *S*_p_-(*R*,*S*)-**1/**(*S*,*S*)-**6**, *S*_p_-(*R*,*S*)-**1/**(*R*,*R*)-**6**, *R*_p_-(*S*,*R*)-**1/**(*S*,*S*)-**6**, and *R*_p_-(*S*,*R*)-**1**/(*R*,*R*)-**6** were also investigated by VCD (Supplementary Figs. 48 and 49), but no clear amplified signals were observed. This may be attributed to the fact that *S*_p_-(*R*,*S*)-**1** and *R*_p_-(*S*,*R*)-**1** are less symmetric hosts than *S*_p_-(*R*,*R*)-**2** and *R*_p_-(*S*,*S*)-**2** and display weaker VCD signals. Furthermore, the geometric positions of the guests inside the chiral hosts **1** and **2** are different (Supplementary Figs. 50–53), which may also lead to this difference in amplification.

## Discussion

In this study we have reported the resolution of chiral porphyrin-cage molecules and the determination of their absolute configurations by VCD/DFT and X-Ray analysis. To the best of our knowledge, our host compounds are the largest molecules so far that have been analyzed by VCD/DFT with respect to the determination of their absolute configurations. The chiroptical properties, ICD, and viologen guest binding abilities of the porphyrin cage compounds were investigated and analyzed. Comparison of DFT calculations with ICD experiments revealed that the viologen guests bind inside the chiral hosts in one specific axial-chiral conformation. For instance, on complexation of the achiral guest methyl viologen in the host *S*_p_-(*R,R*)-**2**, the axial conformation of the guest changes to the (*M*)-enantiomeric form, which is the only one that binds inside the chiral cavity. Such an enantioselective binding and/or positioning is an important first step towards the realization of enantioselective catalytic reactivity within our chiral hosts and suggests that this can be realized with prochiral guest substrates. Upon guest binding, some of the host compounds displayed amplified VCD signals, i.e. by a factor of 1.5–2. To our knowledge, this is the first time that this phenomenon has been observed in host–guest systems. DFT calculations suggest that the VCD signal enhancements are the result of a combination of phenomena, i.e., rigidification of the host and guest structures on complexation, a specific orientation of the guest inside the host, strength of the host–guest binding, charge transfer between the guest and the host, and coupling of specific vibration modes of the host and guest. The results of our present studies will be used for the further construction of a chiral porphyrin-cage catalyst that can move along a polymer chain while writing digital information in the form of chiral epoxides. Work along this line is in progress.

## Methods

### Synthesis

All solvents were freshly dried under argon atmosphere using standard procedures. All reactions were performed under argon using standard Schlenk techniques. NMR spectra were recorded on a Bruker 500 MHz Spectrometer (^1^H: 500 MHz; ^13^C: 125 MHz) at 298 K. ^1^H and ^13^C NMR chemical shifts are reported relative to residual solvent signals. The analytical and preparative resolutions of the porphyrin cage compounds were performed by chiral HPLC (compound **1**: Chiralpak IE, eluent ethanol/dichloromethane 30/70, v/v; compound **2**: Chiralpak IA, and eluent ethanol/ dichloromethane 30/70, v/v). Details are given in the Supplementary Information. The syntheses of porphyrin cage compounds **3** and **4** and those of the guest compounds **5** and **6** were performed according to standard procedures as indicated in the Supplementary Information.

### ECD and UV-Vis spectra

Circular dichroism spectra were measured on a Jasco J-815 CD spectrometer equipped with a JASCO Peltier cell holder PTC-423 to maintain the temperature at 25.0 ± 0.2 °C. A CD quartz cell with 1 mm of optical path length was used. The CD spectrometer was purged with nitrogen before recording each spectrum, from which the baseline was subtracted. The baseline was always measured for the same solvent and in the same cell as the samples. The spectra are presented without smoothing and further data processing. A chiral porphyrin cage compound (H, 0.75 μmol ≈ 1.04–1.08 mg) was dissolved in 25.00 mL of distilled acetonitrile, and the sample was agitated by ultrasonication to dissolve all solid material, resulting in a pink solution. The stoppered flask was then inverted several times to obtain a homogenous host solution ([H] ≈ 30 µM). Viologen guests (G, 4.5 μmol ≈ 1.99-3.28 mg) were dissolved in 5.00 mL of distilled acetonitrile to obtain homogenous guest solutions ([G] ≈ 900 µM). A baseline of the empty cuvette holder, without any cuvette or sample present, was measured. Blanc spectra were recorded using cuvettes filled with distilled acetonitrile. A titration was then carried out by first measuring 500 μL of host solution without any guest present. Guest solution was then added to obtain solutions with a total of 4, 10, 20, 50, 80, and 130 µL of guest solution and 500 μL of host solution (yielding [G]/[H]-ratios between 0.25 and 8). In the case of the mixed solvent system acetonitrile: chloroform = 1:1 (v/v) a titration was carried out by first measuring 500 μL of host solution without any guest present. Guest solution was then added to obtain solutions with a total of 10, 20, and 80 µL of guest solution and 500 μL of host solution (yielding [G]/[H]-ratios between 0.6 and 4.5). After each series, the measured solution was stored to recover the host and the cuvette was flushed with distilled acetonitrile three times. When measuring a new host solution, the cuvette was flushed an additional three times with host solution and then filled with 500 µL of host solution to start a new series of measurements. At the end of the experiments, reference spectra for the chiral guests were measured by mixing 400 µL of distilled acetonitrile with 300 μL of guest solution. ECD- and UV-vis measurements were performed at the same time using a Jasco J-815 CD-spectrometer. The obtained experimental data for the host compounds and the host–guest complexes are shown in the Supplementary Information, Supplementary Figs. 27–39 and Supplementary Table 1.

### Fluorescence titration experiments

The fluorescence experiments were performed using a Jasco FP-8300 spectrophotometer, equipped with a Peltier temperature controller. The machine was allowed to warm at least half an hour before the start of the measurement. All samples were measured in quartz fluorescence cuvettes of 1×1 cm path length, with an internal volume of 3500 µL. The titrations were carried out using the Spectral Measurement-option, using 418 nm as the excitation wavelength and by observing the emission between 425 and 800 nm. The temperature was kept at 25.0 ± 0.1 °C. An excitation bandwidth of 5 nm and an emission bandwidth of 5 nm were used to obtain a good signal-noise ratio, and the scanning speed was set to 1000 nm/min. For the titration experiments a solvent mixture of CHCl_3_/MeCN (1:1 (v/v)) was used to keep both the host and guest soluble. The starting concentration of host was 1.5 µM, to which in ca. 30 steps at least 10 equivalents of guest were added from a concentrated stock solution. The experimental procedure was as follows: a dry 1:1 (v/v) mixture of the above mentioned two solvents was prepared in a volumetric flask. A host-stock solution of 50 µM was made by dissolving the host in the solvent mixture, which was weighed to determine the density. Three host-measurement solutions of 1.5 µM were prepared from a weighed amount of host solution (300 µL), which was diluted to a total volume of 10.00 mL. A guest-stock solution of 200 µM was made in 5.00 mL of the same solvent mixture. Three guest-measurement solutions were prepared, each containing 1.5 µM host and 45 µM guest, which were made by mixing weighed amounts of host-stock (150 µL) and guest-stock (1125 µL) solutions and diluted to a total volume of 5.00 mL. The obtained experimental data are presented in the Supplementary Information, Supplementary Figs. 40–43 and Supplementary Table 2.

### IR and VCD measurements

Infrared (IR) spectra and vibrational circular dichroism (VCD) spectra were recorded using two instruments. The first one was a Vertex70 spectrometer to which a PMA50 optical bench was coupled, both being supplied by Bruker company. In the PMA50 set-up the infrared radiation (3800–600 cm^−1^ range) is focused by a BaF_2_ lens on a ZnSe photo-elastic modulator (PEM, 50 kHz frequency). The circularly polarized beam is then directed onto the sample and finally collected by a D313/QMTC detector. A calibration of the PEM at a fixed wavenumber was performed before recording any VCD spectrum, to ensure a proper chiroptical signal within a spectral region of 600 cm^−1^ around the tuning wavenumber. Typically, calibrations at 1450 cm^−1^ allowed us to obtain a spectrum over the most meaningful region for conjugated organic systems. The second instrument was a JASCO FVS-6000 spectrometer, equipped with a MCT-V detector. The spectrometers were allowed to warm up over the course of 3 h before a measurement was performed. The detector was cooled with liquid nitrogen before and during the measurement. Samples were measured in a BaF_2_ liquid sample cell with a 200-µm optical path length, which was stored in a closed box in the presence of desiccant when not in use. Spectra were acquired with a scan range of 2000–850 cm^−1^, a resolution of 4 cm^−1^, and the aperture set at 5.0 mm. A total of 16 accumulations were obtained for the IR spectra and 3000 accumulations for the VCD spectra. Spectra were processed by the spectrometer software by applying zero filling and apodization using a cosine function. Reference samples for the host were obtained by preparing a 19.5-mM solution of *S*_p_-(*R*,*R*)-**2** (or *R*_p_-(*S*,*S*)-**2**) in CDCl_3_. Reference samples for the guests were obtained by dissolving (*S*,*S*)-**6** (or (*R*,*R*)-**6**) in CD_3_CN with a concentration [**6**] ~ 40 mM. The host–guest mixtures were made from the separately prepared host and guest solutions. Four host solutions were prepared by dissolving 3.0 mg of *S*_p_-(*R*,*R*)-**2** (or *R*_p_-(*S*,*S*)-**2**) in 250 µl of CDCl_3_. Guest solutions were made by dissolving (*S*,*S*)-**6** (or (*R*,*R*)-**6**) in CD_3_CN with a concentration [**6**] ~40 mM. Subsequently, mixtures were prepared by adding 50 µL of the (*S*,*S*)-**6** (or (*R*,*R*)-**6**) solution (1 equiv.) to the desired *S*_p_-(*R*,*R*)-**2** (or *R*_p_-(*S*,*S*)-**2**) solution. The mixtures were briefly swirled to mix the contents and the solvent was then removed from each mixture by blowing a gentle stream of argon gas over the solution. Thereafter, each of the mixtures was re-dissolved in 250 µl of CDCl_3_ and the solvent was removed again by blowing a gentle stream of argon gas over the solution. This step was repeated one more time. Before the measurement the desired mixture was re-dissolved in 150 µl of CDCl_3_ and transferred to the sample cell for measurement ([**2**] ~ [**6**] ~14 mM). Due to the low signal, for host *S*_p_-(*R*,*S*)-**1** (or *R*_p_-(*S*,*R*)-**1**), the applied concentration was 28 mM. The results are presented in the Supplementary Information and Supplementary Figs. 44–49.

### NMR studies on host–guest complexes

The results of these studies are presented in the Supplementary Information and Supplementary Figs. 50–53.

### Calculations of the IR, VCD, UV, and ECD spectra

Firstly, spectra of isolated molecules without explicit solvent molecules were calculated for the *S*_p_-(*R*,*R*)-**2** enantiomer. Only the average effects of the solvent were taken into account using the implicit solvation model SMD (“Solvation Model based on Density”). Each geometry was optimized using Density Functional Theory with the B3LYP functional and triple zeta 6-311G(d) basis set. Empirical dispersion was added with the D3 version of Grimme’s dispersion with Becke-Johnson damping (GD3BJ). The vibrational frequencies, IR absorption, and VCD intensities were calculated using the same level of theory. Frequencies were scaled by a factor of 0.975. IR absorption and VCD spectra were constructed from calculated dipole and rotational strengths assuming Lorentzian band shape with a half-width at half maximum of 8 cm^−1^. All calculations were performed using the Gaussian16 package. The conformations selected for the calculations of the averaged IR/VCD spectra of *S*_p_-(*R*,*R*)-**2** were obtained by molecular dynamics calculations, see below. Eight conformations were selected and optimized. Boltzmann populations estimated from enthalpies calculated at 298 K revealed that the 5 most stable conformations among the 8 found are required for the calculation of the averaged spectra (see Supplementary Information, Supplementary Table 3, and Supplementary Fig. 54). In this way, an acceptable but not satisfying agreement was obtained between the spectra measured for the enantiomer (+)−**2** and calculated for the enantiomer *S*_p_-(*R*,*R*)-**2** (see Supplementary Information and Supplementary Fig. 55). Calculations performed by introducing an explicit solvent molecule inside the two most stable conformations found for *S*_p_-(*R*,*R*)-**2** made the agreement between measurement and calculation significantly more satisfactory. The position of the explicit solvent molecule (CD_2_Cl_2_) in the cage of porphyrin *S*_p_-(*R*,*R*)-**2** was initially determined using molecular dynamics calculations and thereafter the complex was optimized using the SMDCD_2_Cl_2_/GD3BJ-B3LYP/6-311 G(d) DFT level. Given the quality of this result with only two conformations and considering the large size of the studied system for which a significant amount of calculation time is required, this model was not extended to other conformations. The results obtained for compound **2** were also used for the calculations of the spectra of the *S*_p_-(*R*,*S*)-**1** enantiomer, as well as for the calculations of the UV and ECD spectra of both compounds **1** and **2**. The geometries used for the *S*_p_-(*R*,*S*)-**1** compound were optimized from the geometries of the retained conformations of *S*_p_-(*R*,*R*)-**2** in which one of the two nitro groups was replaced by a hydrogen atom. As with **2**, a good balance between accuracy of the results and the consumed cpu time was obtained by considering only two conformations with one explicit solvent molecule using the SMD_CD2Cl2_/GD3BJ-B3LYP/6-311G(d) theoretical level. For the calculations of the UV and ECD spectra a similar approach was applied but with an explicit CH_3_CN molecule instead of CD_2_Cl_2_. The geometry optimizations were performed using WB97XD functional associated with the 6-31G(d) basis set and the SMD implicit solvent model. Based on these geometries, the ECD and UV spectra were calculated using the time-dependent density functional theory (TD-DFT) with LC-WhPBE functional and def2SVP basis set. Calculations were performed for vertical 1A singlet excitation using 100 states. For a comparison between theoretical results and the experimental values, the calculated UV and ECD spectra were modeled with a Gaussian function using a half-width of 0.37 eV. Due to the approximations of the used theoretical model, an almost constant offset was observed between measured and calculated wavelengths. Using UV spectra, all calculated wavelengths were calibrated by a factor of 1.05.

### Molecular dynamics calculations

Molecular dynamics (MD) simulations were performed starting from the atomistic coordinates of the optimized structure obtained from quantum chemical calculation. The porphyrin molecule was inserted in a cubic box whose sides measured 15 Å and solvated with ~1000 molecules of dichloromethane or acetonitrile. We initially minimized the energy and then performed equilibrium MD under periodic boundary conditions in NPT ensemble. MD trajectory was followed for 100 ns in NVT ensemble. The temperature during the simulation was held constant at 300 K using Berendsen thermostat. Fast smooth Particle-Mesh Ewald summation was used for long-range electrostatic interactions, with a cutoff of 1.0 nm for the direct interactions. We used the clustering analysis tool of GROMACS (gmx cluster) to explore the different conformations from the MD trajectory. The GROMOS clustering algorithm with a RMSD cut-off was used to determine the structurally similar clusters. This approach allowed us to select eight geometries that were optimized and used for the calculations of the average IR and VCD spectra (Supplementary Information and Supplementary Fig. 55). The same approach was used to the selection of geometries of the cage porphyrin with a solvent molecule inside (Supplementary Information and Supplementary Fig. 54).

### VCD bands amplification and Induced CD phenomenon

The geometries the host *S*_p_-(*R*,*R*)-**2** and guests **5**, (*S*,*S*)-**6**, and (*R*,*R*)-**6** were optimized using the WB97XD functional software associated with the 6-311 G(d) basis set but without implicit solvent effects in order to keep a reasonable use of cpu time. In order to better understand the Induced CD phenomenon, we optimized one conformation of the complexes formed by *S*_p_-(*R,R*)-**2** and the two enantiomorphic conformations of viologen **5**. Two possibilities were considered: viologen **5** is hosted in a ‘horizontal’ orientation (perpendicular to the xylylene sidewalls and parallel to the porphyrin ring) or in a ‘vertical’ position orientation (parallel to the xylylene side walls and vertical to the porphyrin ring) in the cavity of the host. Attempts to optimize the complex *S*_p_-(*R*,*R*)-**2**/(*M*)-**5** starting from geometries in which the guest is in a ‘horizontal’ position all converged towards a conformation in which **5** had rotated to a ‘vertical’ position. Similarly, all attempts to optimize the *S*_p_-(*R*,*R*)-**2**/(*P*)-**5** complex converged to the *S*_p_-(*R*,*R*)-**2**/(*M*)-**5** complex. Based on the optimized geometries of (*M*)-**5**, *S*_p_-(*R*,*R*)-**2**, and the complex *S*_p_-(*R*,*R*)-**2**/(*M*)-**5**, the corresponding ECD and UV spectra were calculated using time-dependent density functional theory (TD-DFT) with the LC-WhPBE functional and def2SVP basis set. Calculations were performed for vertical 1A singlet excitation using 50 states. In order to compare the theoretical results with the experimental values, the calculated UV and ECD spectra were modeled with a Gaussian function using a half-width of 0.37 eV. Due to the approximations of the used theoretical model an almost constant offset was observed between the measured and calculated wavelengths. Using UV spectra, all calculated wavelengths were calibrated by a factor of 1.05. For the *S*_p_-(*R*,*R*)-**2**/(*M,S,S*)-**6** and *S*_p_-(*R*,*R*)-**2**/(*M,R,R*)-**6** complexes, we looked for the most stable conformations adopted by the side chains of the viologens complexed in the cavity. We used a methodology combining DFT calculations and simulated annealing performed at the semi-empirical level. From an initial geometry of the complex optimized using the WB97XD/6-311G(d) DFT level, we carried out a simulated annealing at the AM1 semi-empirical level, allowing the side chains of the viologen to adopt different conformations on the dihedral angles only. The bond lengths, valence angles, and dihedral angles not involved in the conformational mobility of the viologen side chains were fixed in these simulated annealing calculations. The lower energy conformation found by this approach was then fully optimized using the WB97XD/6-311G(d) level (Supplementary Information and Supplementary Fig. 54) before calculating the IR and VCD spectra (Supplementary Information and Supplementary Fig. 56). The AMPAC10 program was used for all the semi-empirical simulated annealing calculations.

### X-ray structures

Single crystals of **(−)-***R*_p_-(*S*,*R*)-**1** and **(+)-***S*_p_-(*R*,*S*)-**3** were both grown from a mixture of CHCl_3_ and CD_3_CN (1:1 v/v). Reflections were measured on a Bruker D8 Quest diffractometer with sealed tube and Triumph monochromator (*λ* = 0.71073 Å). Software package used for the intensity integration was Saint. Absorption correction was performed with SADABS. The structures were solved with direct methods using SHELXT. Least-squares refinement was performed with SHELXL-2014 against $\left| {{\mathrm{F}}_{\mathrm{h}}^{\mathrm{o}}} \right|^2$of all reflections. Non-hydrogen atoms were refined freely with anisotropic displacement parameters. Hydrogen atoms were placed on calculated positions or located in difference Fourier maps. All calculated hydrogen atoms were refined with a riding model. Experimental data is presented in the Supplementary Information, Supplementary Table 4, and Supplementary Figs. 57 and 58.

## Supplementary information

Supplementary Information

## Data Availability

The syntheses, resolution, and characterization of the compounds, the ECD and VCD measurements, the DFT calculations, and the host–guest binding studies are described in the Supplementary Information. Crystallographic data have been deposited at the Cambridge Crystallographic Data Centre (CCDC) under CCDC numbers 1989977 for *R*_p_-(*S*,*R*)-**1** and 1989978 for *S*_p_**-**(*R*,*S*)-**3**. Any further relevant data are available from the corresponding authors on request.
