# Supplementary material for: Absolute configuration and host-guest binding of chiral porphyrin-cages by a combined chiroptical and theoretical approach
Source: Nat Commun. 2020 Sep 22;11:4776. doi: 10.1038/s41467-020-18596-1 (PMC7508876; doi:10.1038/s41467-020-18596-1)
Supplement: Supplementary file 1 — Supplementary Information [file 41467_2020_18596_MOESM1_ESM.pdf]

Supplementary Information

Absolute configuration and host-guest binding of chiral porphyrin-cages by a combined chiroptical and theoretical approach

Jiangkun Ouyang, et al.

Supplementary Methods

Materials and methods

All solvents were freshly dried under argon atmosphere using standard procedures. All reactions were performed under argon using standard Schlenk techniques. NMR spectra were recorded on a Bruker 500 MHz Spectrometer (^{1}H: 500 MHz; ^{13}C: 125 MHz) at 298 K. ^{1}H and ^{13}C NMR chemical shifts are reported relative to residual solvent signals. Circular dichroism spectra were measured on a Jasco J-815 CD spectrometer equipped with a JASCO Peltier cell holder PTC-423 to maintain the temperature at 25.0 ± 0.2°C. A CD quartz cell with 1 mm of optical path length was used. The CD spectrometer was purged with nitrogen before recording each spectrum, from which the baseline was subtracted. The baseline was always measured for the same solvent and in the same cell as the samples. The spectra are presented without smoothing and further data processing. Fluorescence spectra were measured on a JASCO FP-8300ST spectrofluorometer. Infrared (IR) spectra and vibrational circular dichroism (VCD) spectra were performed in a PMA50 optical bench coupled to a Vertex70 spectrometer, which were both supplied by Bruck company.

Chiral HPLC separations

Analytical chiral HPLC separation of the enantiomers of mono-nitro porphyrin cage 1

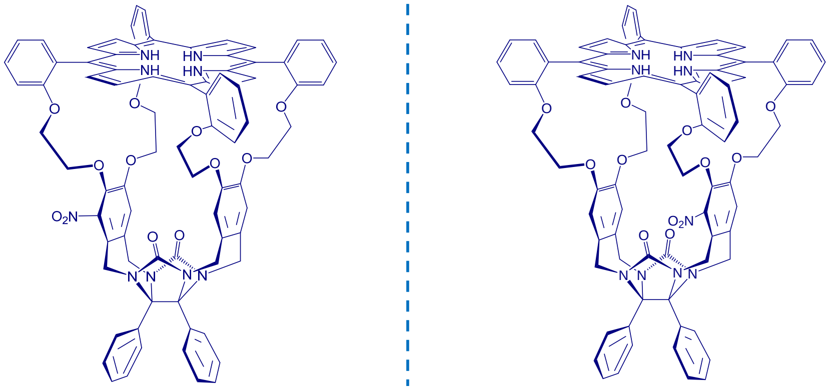

The sample was dissolved in dichloromethane, injected onto the chiral column, and the enantiomers detected by UV-Vis (λ 420 nm) and CD (λ 254 nm). The flow-rate was 1 mL/min.

Column	Mobile Phase	t1	k1	t2	k2	α	Rs
Chiralpak IE	Ethanol / dichloromethane (30/70)	4.25 (-)	0.44	7.41 (+)	1.51	3.42	6.53

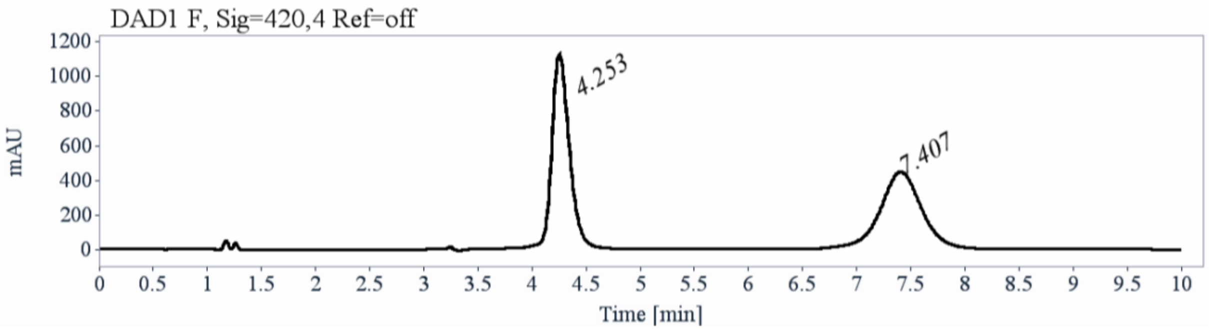

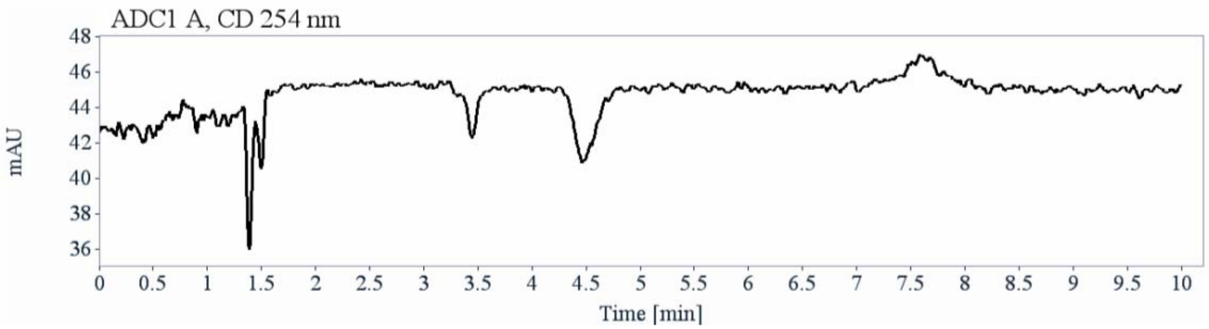

RT [min]	Area	Area%	Capacity Factor	Enantioselectivity	Resolution (USP)
4.25	12640	51.14	0.44		
7.41	12077	48.86	1.51	3.42	6.53
Sum	24718	100.00			

Preparative separation of the enantiomers of mono-nitro porphyrin cage 1

Sample preparation: About 200 mg of racemic compound **1** was dissolved in 33 mL of a mixture of ethanol / dichloromethane (25/75).

Chromatographic conditions: Chiralpak IE (250 x 10 mm), Ethanol / dichloromethane (30/70) as mobile phase, flow-rate = 5 mL/min, UV detection at 254 nm.

Injections (stacked): 47 times 700 µL.

First fraction: 97 mg of the first eluted enantiomer with ee > 99.5%.

Second fraction: 94 mg of the second eluted enantiomer with ee > 99.5%.

Chromatograms of the collected fractions:

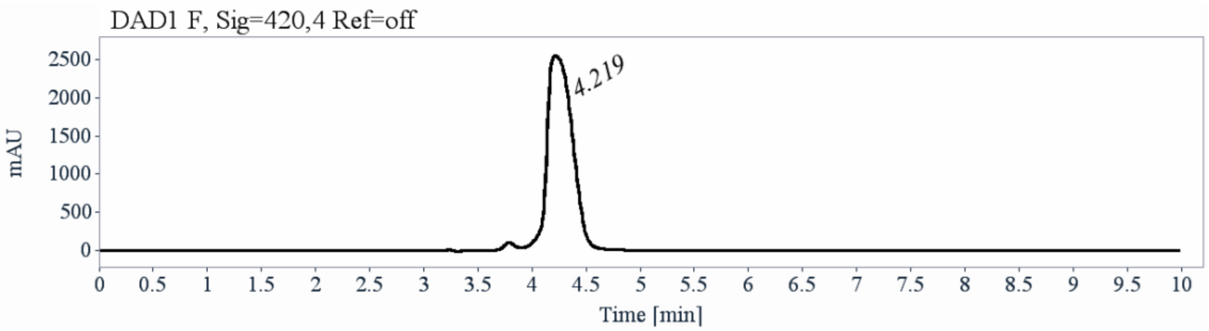

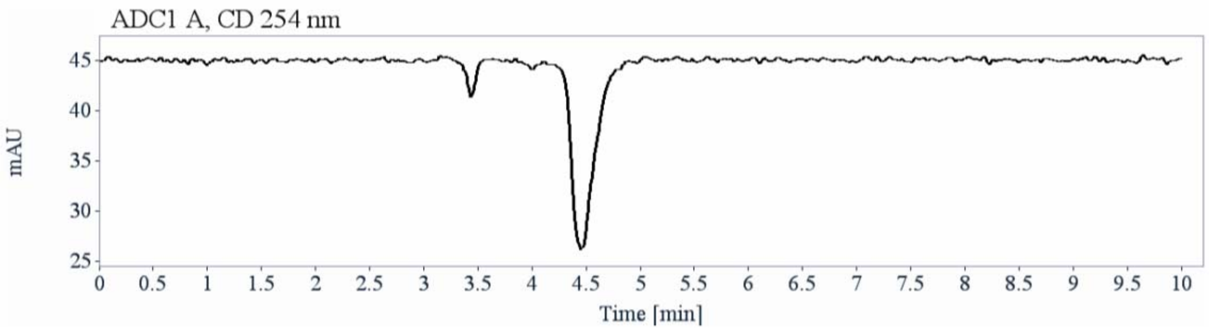

RT [min]	Area	Area%
4.22	40388	100.00
Sum	40388	100.00

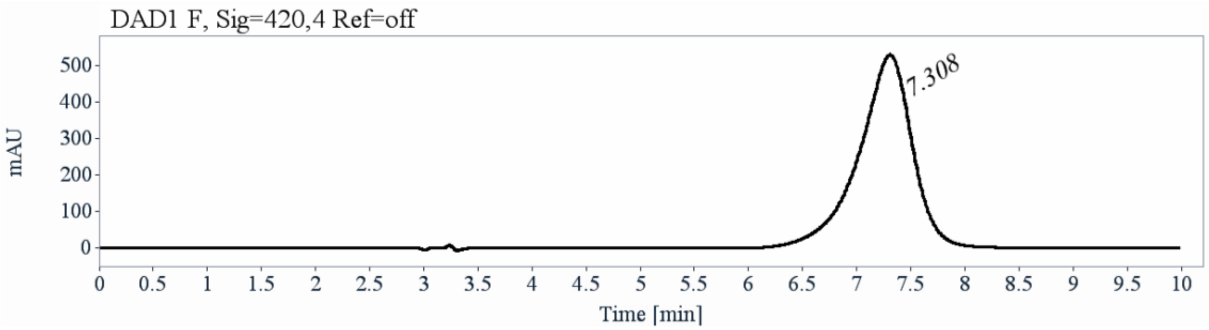

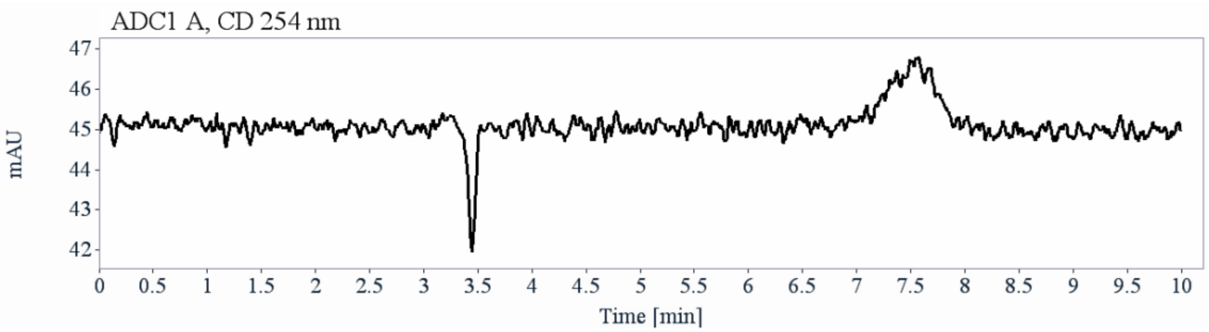

RT [min]	Area	Area%
7.31	18162	100.00
Sum	18162	100.00

Analytical chiral HPLC separation of the enantiomers of anti-dinitro porphyrin cage 2

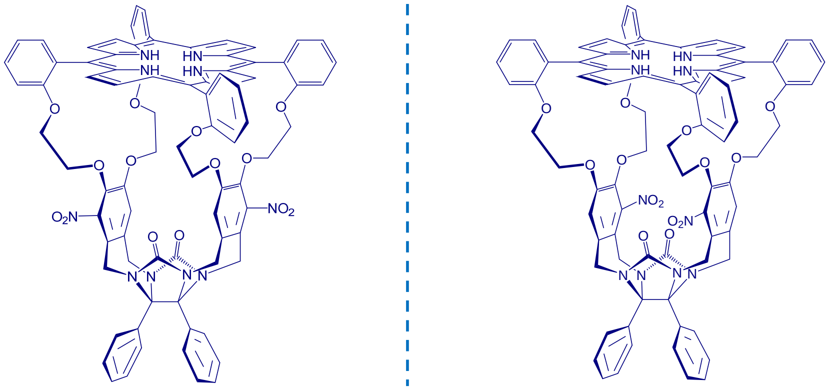

The sample was dissolved in dichloromethane, injected onto the chiral column, and the enantiomers detected by UV-Vis (λ 420 nm) and CD (λ 254 nm). The flow-rate was 1 mL/min.

Column	Mobile Phase	t1	k1	t2	k2	α	Rs
**Chiralpak IA**	Ethanol / dichloromethane (30/70)	3.09 (-)	0.05	5.27 (+)	0.79	16.99	7.91

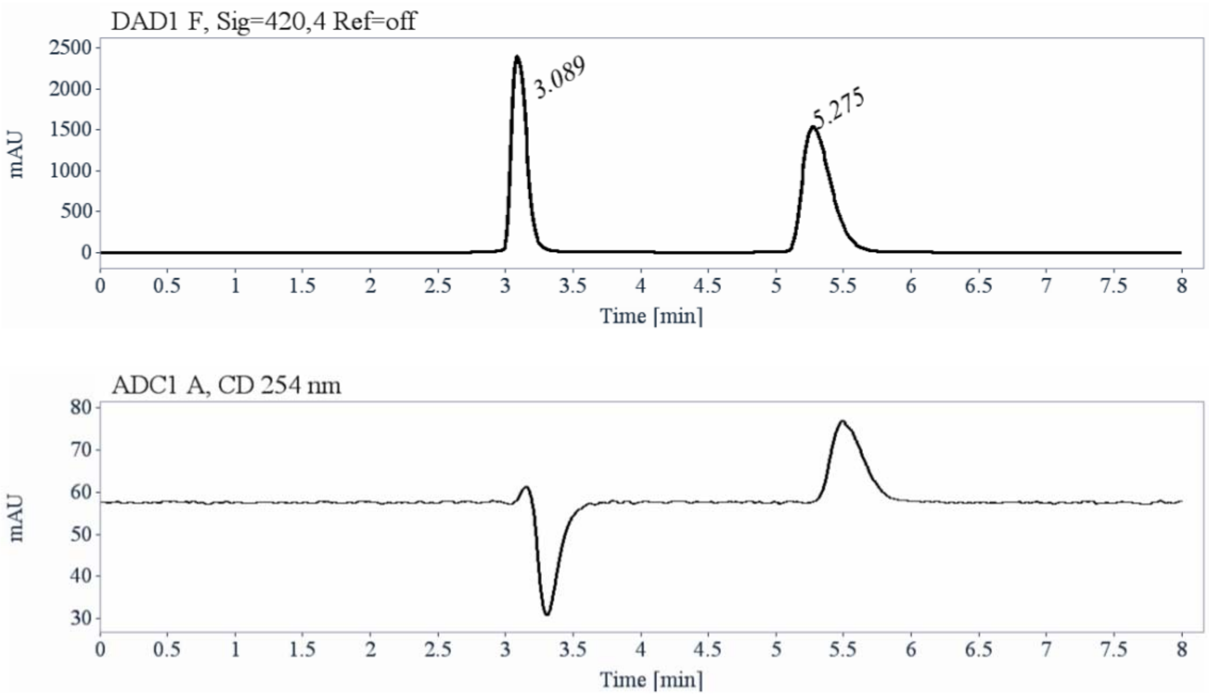

RT [min]	Area	Area%	Capacity Factor	Enantioselectivity	Resolution (USP)
3.09	1326	53.91	0.05		
5.27	1134	46.09	0.79	16.99	7.91
Sum	2460	100.00			

Preparative separation of the enantiomers of anti-dinitro porphyrin cage 2

Sample preparation: About 60 mg of racemic **2** was dissolved in 5.1 mL of a mixture of ethanol / dichloromethane (20/80).

Chromatographic conditions: Chiralpak IA (250 x 10 mm), Ethanol / dichloromethane (30/70) as mobile phase, flow-rate = 5 mL/min, UV detection at 254 nm.

Injections (stacked): 17 times 300 µL, every 3.5 minutes.

First fraction: 5.0 mg of the first eluted enantiomer with ee > 99.5%.

Second fraction: 5.0 mg of the second eluted enantiomer with ee > 99.5%.

Chromatograms of the collected fractions:

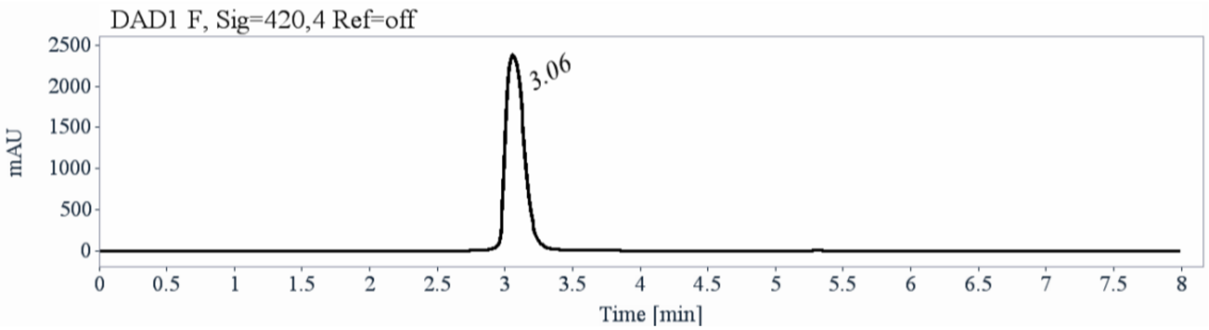

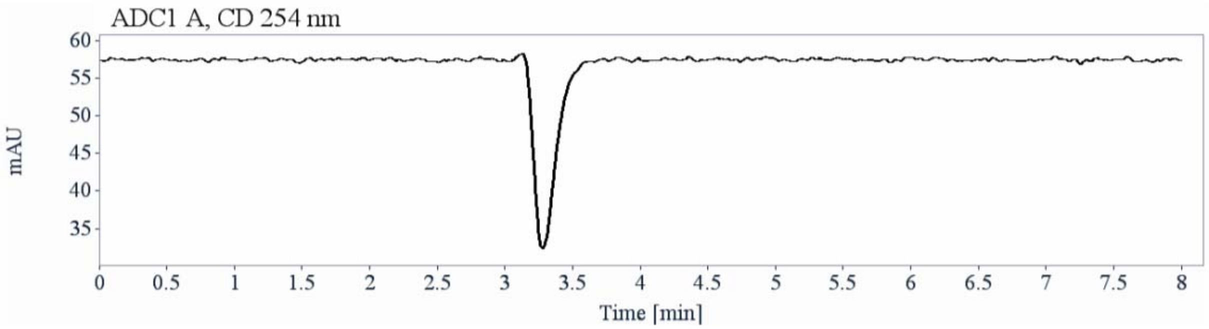

RT [min]	Area	Area%
3.06	1474	100.00
Sum	1474	100.00

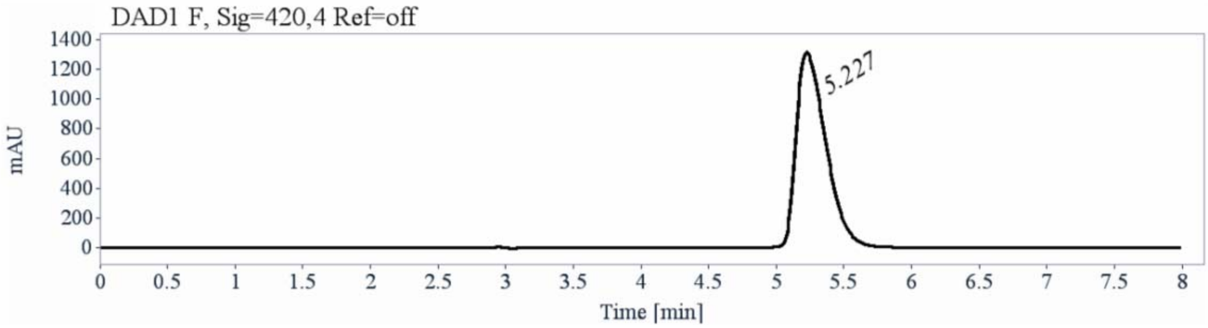

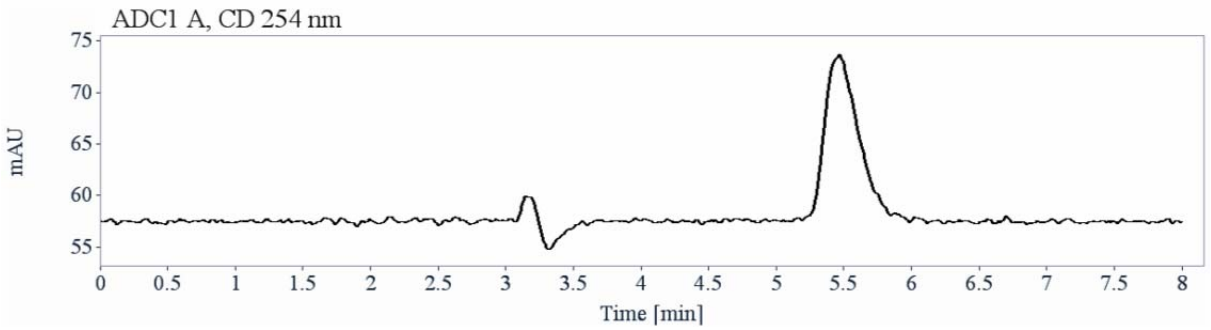

RT [min]	Area	Area%
5.23	993	100.00
Sum	993	100.00

Optical rotations

Optical rotations of the enantiomers could not be measured due to the intense purple color of the compounds.

Assignment of chirality to porphyrin cages 1 and 2

The introduction of one nitro group or two nitro groups to the sidewalls of the original porphyrin cage molecule (Supplementary Figure 1, compounds **1** and **2**), leads to symmetry breaking. For both **1** and **2** two types of chirality, i.e. planar chirality and point chirality, are generated at the same time. To assign the planar chirality, the two enantiomers of the two cages were first rotated 90° to the left and to the right, respectively (Supplementary Figure 1). Then, a plane containing 4 atoms (labeled as A, B, X, and Y with A ≠ B) was selected. A fifth atom Z, named the pilot atom, located above the plane and restricted in its rotation around the Y-X axis, was chosen .[1-5] Because atom A is closest to the nitro group, it has priority with respect to atom B, according to the chirality sequence rule. Starting from pilot atom Z and looking at the plane via the tracing path Y-X-A, the rotation can be counterclockwise (S_p) or clockwise (R_p). In the case of mono-nitro porphyrin cage **1**, the planar chiralities (S_p) and (R_p) combined with the point chiralities present at the quaternary carbon atoms of the glycoluril skeleton, i.e. (*R*,*S*) and (*S*,*R*), respectively, lead to the chirality assignments of S_p-(*R*,*S*) and R_p-(*S*,*R*), for the two enantiomers, respectively. A similar procedure can be followed for anti-dinitro porphyrin cage **2**, leading to the assignments of S_p-(*R*,*R*) and R_p-(*S*,*S*) for the two enantiomers, respectively.

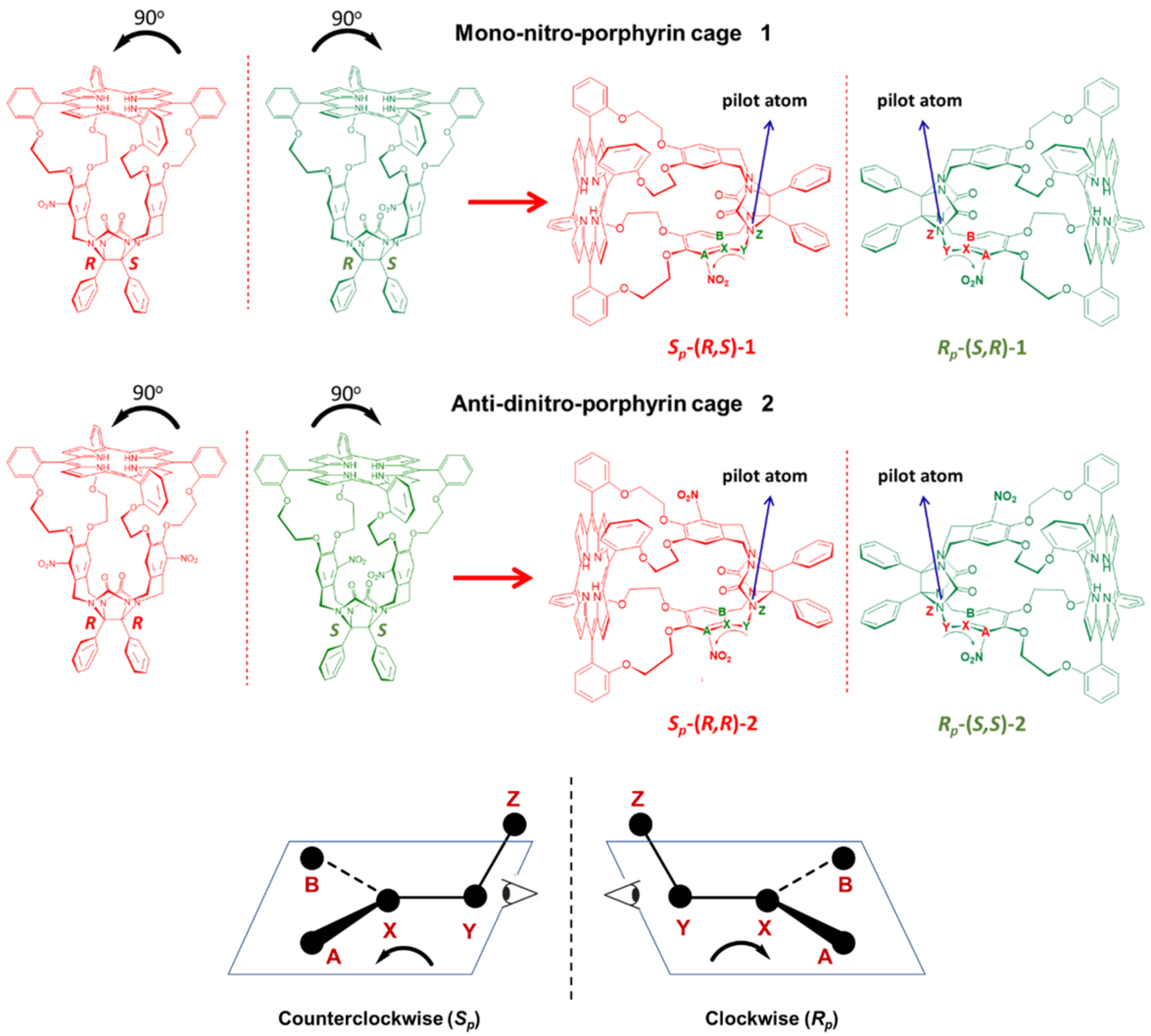

Supplementary Figure 1 Assignment of absolute configuration. Assignment of the chirality to the mono-nitro porphyrin cage **1** and the anti-dinitro porphyrin cage **2**, and the drawings of the chirality tracing paths .

Synthesis and characterization of zinc chiral porphyrin cages 3 and 4

Pophyrin cage 3

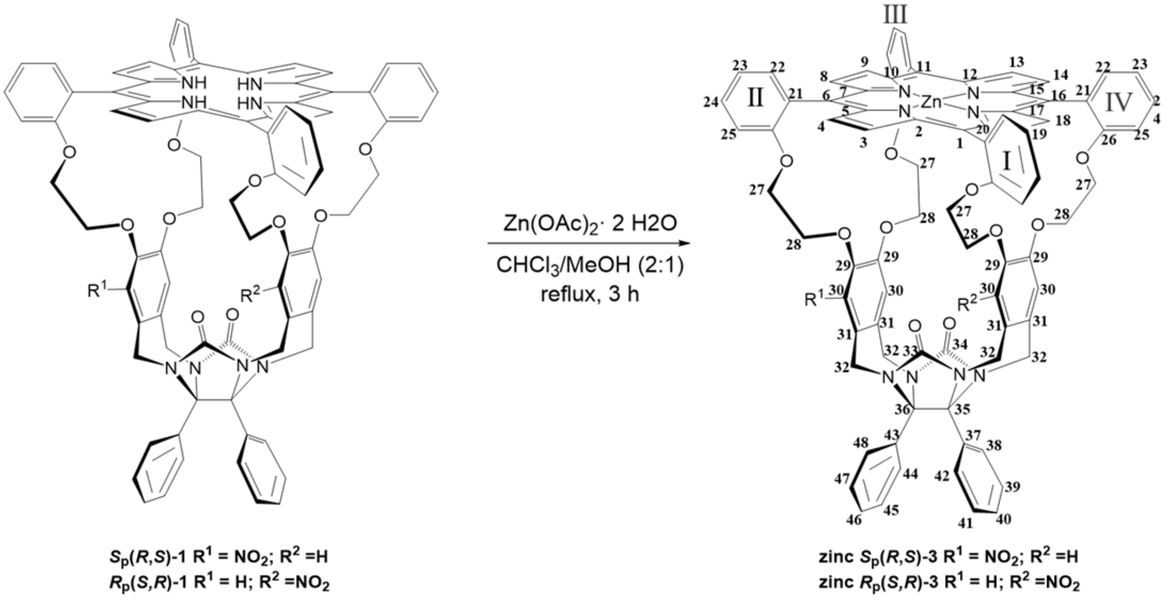

A flask was charged with one of the enantiomers of mono-nitro porphyrin cage, i.e. (S_p-(R,S)-**1** or R_p-(S,R)-**1**), (10 mg, 0.007 mmol) in a degassed mixture of chloroform (2 mL) and methanol (1 mL). Then, zinc acetate dihydrate (6.5 mg, 0.035 mmol) was added. The mixture was excluded from light and refluxed under argon for 3 h. After cooling, the solvent was evaporated and the residue was dissolved in dichloromethane (10 mL). The organic layer was washed with water (2×) and concentrated in vacuo. The residue was purified by column chromatography (chloroform/acetonitrile, 15:1 (v/v)). Yields: 10 mg (96%) of zinc S_p-(R,S)-**3** and 9.7 mg (93%) of zinc R_p-(S,R)-**3** as purple solids. Melting points of the enantiomers: > 300 ℃. Optical rotations could not be measured due to the intense purple color of the enantiomers.

NMR

The NMR spectra of the two enantiomers are identical. Below, the NMR spectral data for zinc S_p-(*R*,*S*)-**3** are given.

^{1}H-NMR (500 MHz, Chloroform-*d*) δ 8.95 (d, 1H, β-pyrrole-H-**8**, *J* = 4.7 Hz), 8.90 (d, 1H, β-pyrrole-H-**18**, *J* = 4.7 Hz), 8.87 (d, 1H, β-pyrrole-H-**9**, *J* = 4.6 Hz), 8.84 (d, 1H, β-pyrrole-H-**19**, *J* = 4.6 Hz), 8.79 (d, 1H, β-pyrrole-H-**13**, *J* = 4.6 Hz) 8.77 (d, 1 H, β-pyrrole-H-**14**, *J* = 4.6 Hz), 8.74 (d, β-pyrrole-H-**3**, *J* = 4.6 Hz), 8.71 (d, β-pyrrole-H-**4**, *J* = 4.6 Hz), 8.38 (dd, 1H, ArH-**22**(**II**), *J* = 7.3, 1.7 Hz), 8.22 (dd, 1H, ArH-**22**(**IV**), *J* = 7.3, 1.7 Hz), 8.03 (dd, 1H, ArH-**22**(**III**), *J* = 7.3, 1.7 Hz), 7.95 (dd, 1H, 1H, ArH-**22**(**I**), *J* = 7.3, 1.7 Hz), 7.78-7.73 (m, 4H, ArH-**24** (**I**, **II**, **III**, **IV**), 7.48 (t, 1H, ArH-**23**(**II**), *J* = 7.2, 7.2 Hz), 7.42 (t, 1H, ArH-**23**(**IV**), *J* = 7.4, 7.4 Hz), 7.37-7.32 (m, 5H, ArH-**23**(**I**, **III**), **25**(**I**, **III**, **IV**)), 7.23 (d, 1H, ArH-**25**(**II**), *J* = 7.2 Hz), 7.08-7.02 (m, 2H, ArH-**41**,**45**), 6.99-6.94 (m, 2H, ArH-**40**,**46**), 6.90-6.82 (m, 4H, ArH-**39**,**42**,**44**,**47**), 6.67 (dt, 1H, ArH-**38**, *J* = 7.7, 7.7 Hz), 6.28 (s, 1H, ArH-**30**(**IV**)), 6.23 (s, 1H, ArH-**30**(**III**)), 6.05 (s, 1H, ArH-**30**(**I**)), 4.37-4.30 (m, 2H, CH_2-**32a**(**II**, **III**)), 4.27-4.21 (m, 4H, CH_2-**32a**(**I**), **27a**(**I**, **III**, **IV**)), 4.16-4.04 (m, 2H, CH_2-**32a**(**IV**), **27a**(**II**)), 4.01-3.94 (m, 3H, CH_2-**27b**(**I**, **II**, **III**)), 3.78-3.64 (m, 4H, CH_2-**32b**(**I**, **II**, **III**, **IV**), 3.58-3.56 (m, 1H, CH_2-**28a**(**I**)), 3.48-3.39 (m, 4H, CH_2-**28a**(**III**, **IV**), CH_2-**28b**(**III**, **IV**)), 3.24 (t, 1H, CH_2-**28b**(**I**)), 3.10 (m, 1H, CH_2-**28a**(**II**)), 2.43 (m, 1H, CH_2-**28b**(**II**)).

^{13}C NMR (126 MHz, Chloroform-d) δ 158.86 (ArC-**26**(**II**)), 158.84 (ArC-**26**(**III**)), 158.72 (ArC-**26**(**IV**)), 158.48 (ArC-**26**(**I**), 156.88(C=O-**34**), 156.34 (C=O-**33**), 150.63 (C-**2**, **5**), 150.61 (ArC-**29**(**III**)), 150.20 (C-**7**), 150.17 (C-**12**, **15**), 150.12 (C-**10**), 149.78 (C-**20**), 149.60 (C-**17**), 147.38 (C-**29**(**I**)), 145.97 (ArC-**30**(**II**)), 145.36 (C-**29**(**IV**)), 138.19 (C-**29**(**II**)), 135.92 (ArC-**22**(**I**)), 135.58 (ArC-**22**(**III**)), 135.45 (ArC-**22**(**IV**)), 134.08 (ArC-**22**(**II**)), 131.59 (C-**13**), 131.38 (C-**14**), 132.86 (ArC-**21**(**IV**)), 132.37 (ArC-**21**(**II**)), 132.35 ((ArC-**21**(**II**)), 132.22 (131.32 (C-**9**), 131.56 (131.30 (C-**3**), 131.20 (C-**4**), 131.10 (C-**8**), 130.86 (C-**19**), 130.59 (C-**18**), 129.70 (C-**24**(**II**)), 129.46 (C-24(**IV**)), 129.39 (C-**24**(**I**, **III**)), 128.89 (ArC-**40**), 128.82 (ArC-**46**), 128.76 (ArC-**39**), 128.66 (ArC-**47**), 128.54 (ArC-**41**), 128.52 (ArC-**45**), 128.29 (ArC-**38**), 127.61 (ArC-**42**), 127.39 (ArC-**44**), 120.97 (C-**31**(**II**)), 120.12 (ArC-**23**(**II**)), 120.04 (ArC-**23**(**IV**)), 119.73

(ArC-**23**(**I**)), 119.69 (ArC-**23**(**III**)), 117.39 (ArC-**30**(**IV**)), 116.14 (ArC-**1**), 116.12 (ArC-**11**), 116.10 (ArC-**16**), 116.04 (ArC-**6**), 114.47 (ArC-**30**(**III**)), 113.24 (ArC-**30**(**I**)), 112.46 (ArC-**25**(**II**)), 112.43 (ArC-**25**(**IV**)), 111.73 (ArC-**25**(**I**)), 111.21 (ArC-**25**(**III**)), 85.01 (C-**35**), 84.28 (C-**36**), 71.61 (C-**28**(**II**)), 68.20 (C-**28**(**III**, **IV**)), 67.32 (C-**27**(**I**)), 67.08 (C-**27**(**III**)), 66.78 (C-**27**(**IV**)), 66.73 (C-**28**(**I**)), 65.53 (C-**27**(**II**)), 44.73 (C-**32**(**IV**)), 44.46 (C-**32**(**III**)), 44.13 (C-**32**(**I**)), 38.78 (C-**32**(**II**)).

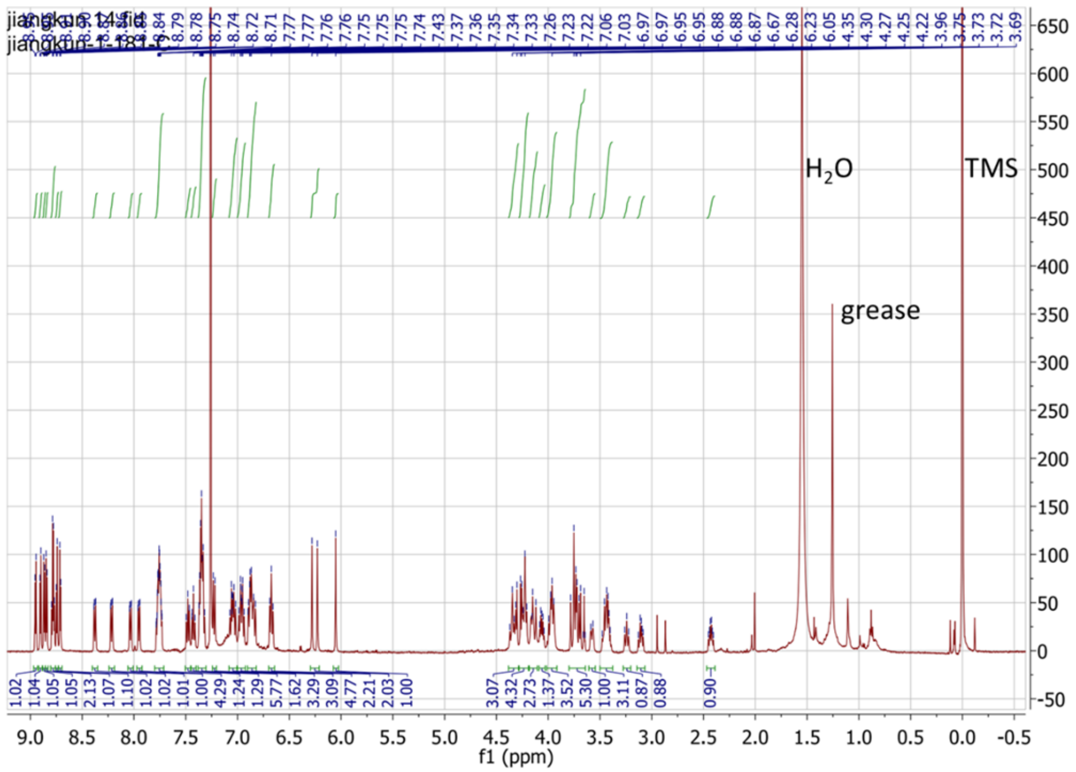

Supplementary Figure 2 ^{1}H NMR spectrum (500 MHz, 298 K) of zinc S_p-(R,S)-**3** in $CDCl_3$.

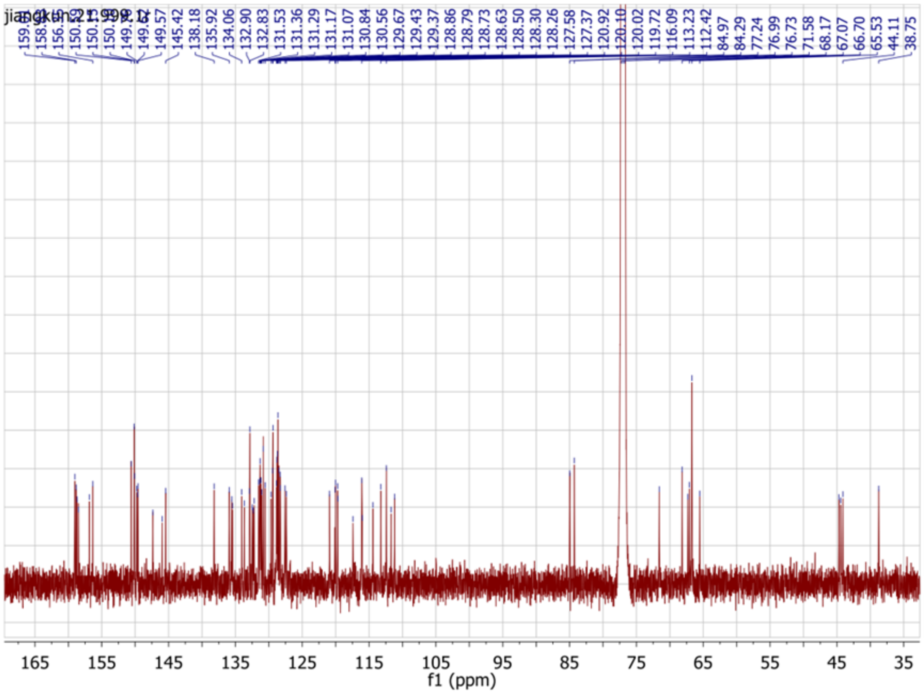

Supplementary Figure 3 ^{13}C NMR spectrum (126 MHz, 298 K) of zinc S_p-(R,S)-**3** in $CDCl_3$

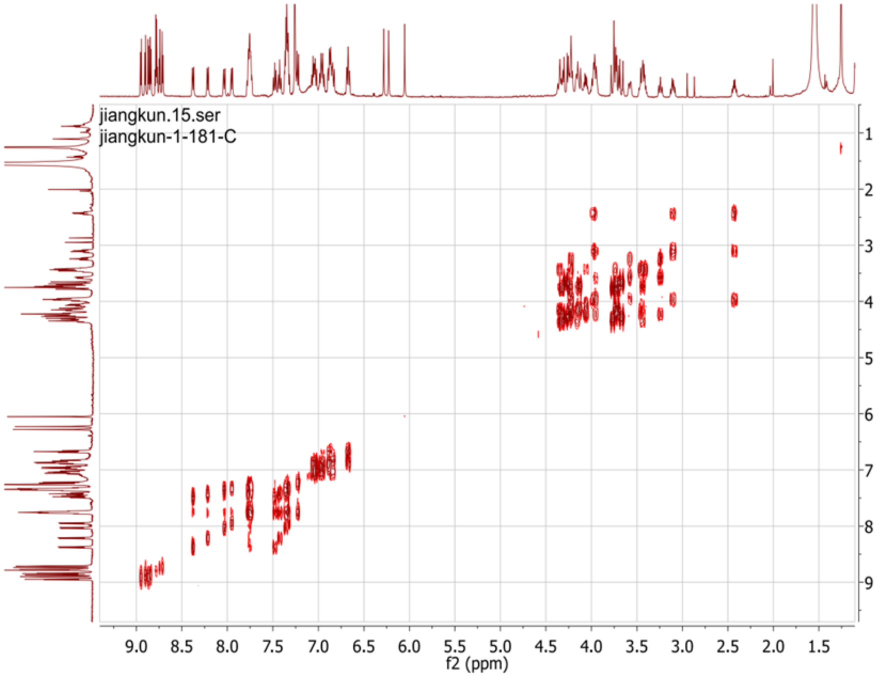

Supplementary Figure 4 ^{1}H-^{1}H COSY NMR spectrum (500 MHz, 298 K) of zinc S_p-(R,S)-**3** in $CDCl_3$.

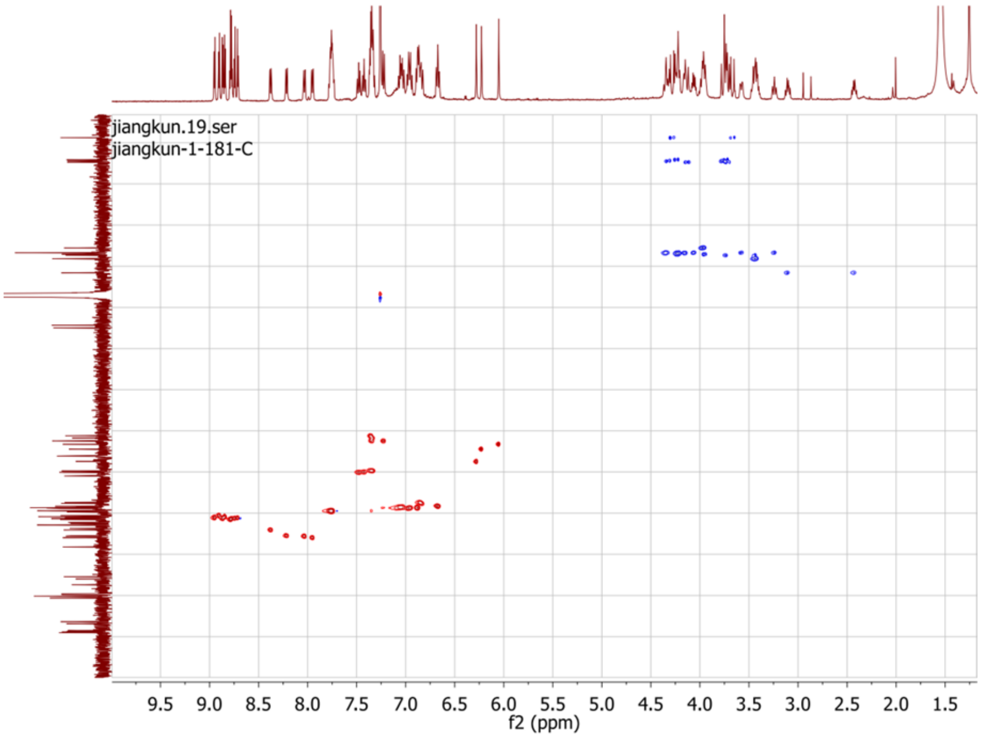

Supplementary Figure 5 ^{1}H-^{13}C HSQC NMR spectrum (500 MHz, 298 K) of zinc S_p-(R,S)-**3** in $CDCl_3$. CH_2 groups are indicated in red and CH/CH_3 groups in blue.

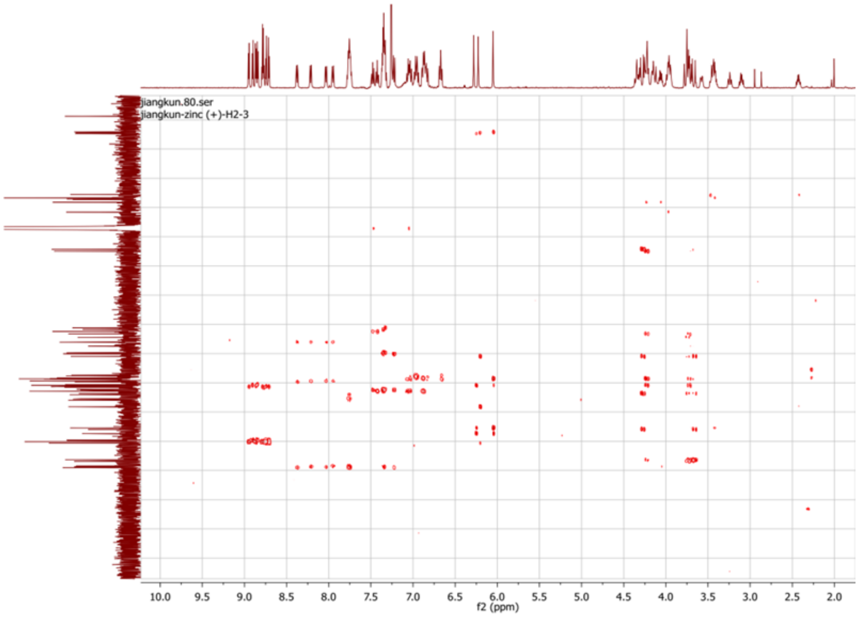

Supplementary Figure 6 ^{1}H-^{13}C HMBC NMR spectrum (500 MHz, 298 K) of zinc S_p-(R,S)-**3** in $CDCl_3$.

MS

MALDI-TOF MASS: m/z: 1453.871 $(M+H)^+$; calculated for $C_{84}H_{60}N_9O_{12}Zn + H^+$ m/z: 1453.84 (the masses of two enantiomers are identical).

a

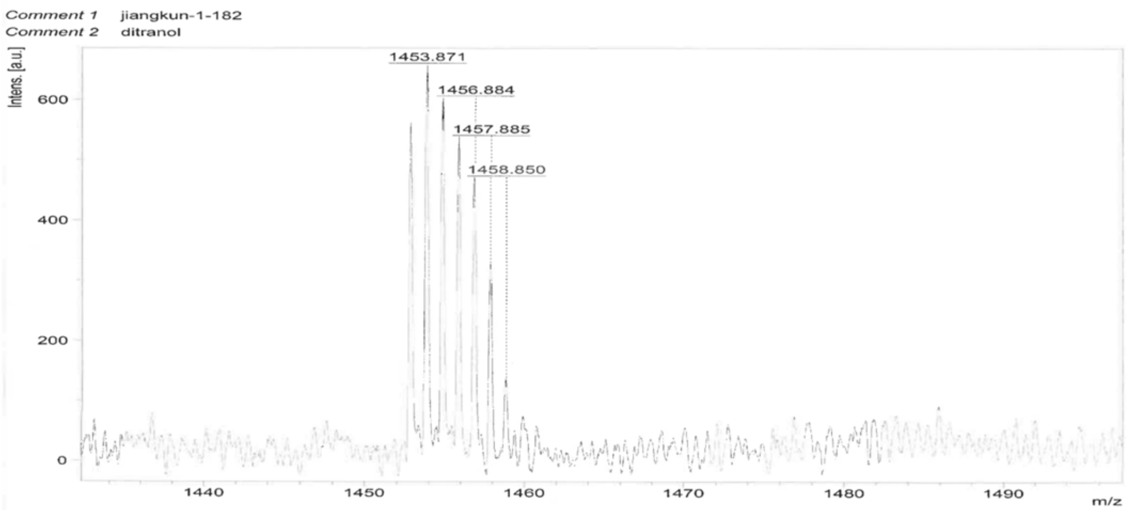

D:\Data_Nijmegen_FNWI\Chemistry\Nolte\Jiangkun\jiangkun-1-182\0_F8\1\1SRef

m/z	S/N	Quality Fac.	Res.	Intens.	Area
1451.880	8	17.6	18539	374	80.3
1452.886				562	
1453.871	14	2665	5944	630	393
1455.869	9	311	6418	423	238
1456.884				472	
1457.885				328	
1458.850				137	

b

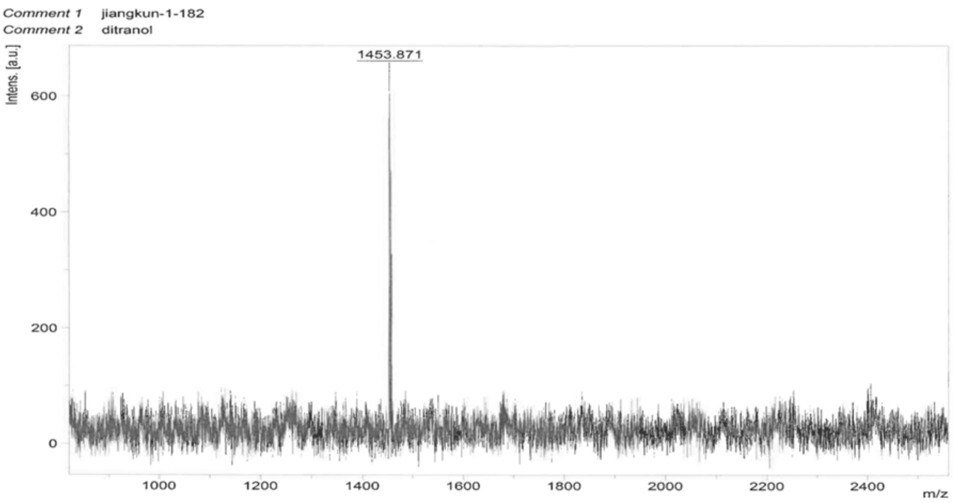

D:\Data_Nijmegen_FNWI\Chemistry\Nolte\Jiangkun\jiangkun-1-182\0_F8\1\1SRef

m/z	S/N	Quality Fac.	Res.	Intens.	Area
1451.880	8	17.6	18539	374	80.3
1453.871	14	2665	5944	630	393
1455.869	9	311	6418	423	238

Supplementary Figure 7 MALDI-TOF MASS spectra of zinc S_p-(*R*,*S*)-**3**. **a** Magnification. **b** Full spectrum.

For the HRMS-ESI study a solution containing CsI was used, because many mass peaks were present in its absence, e.g. those corresponding to zinc mono-nitro porphyrin cage with MeOH, MeCN, and with TFA. Cs^+ can bind to the porphyrin cage, and Cs^+ has 100% abundance. The detected signal is [M + Cs^+]. HRMS-ESI: m/z: 1584.2742 $(M + Cs)^+$ calculated for $C_{84}H_{61}CsN_9O_{12}Zn$ 1584.27855.

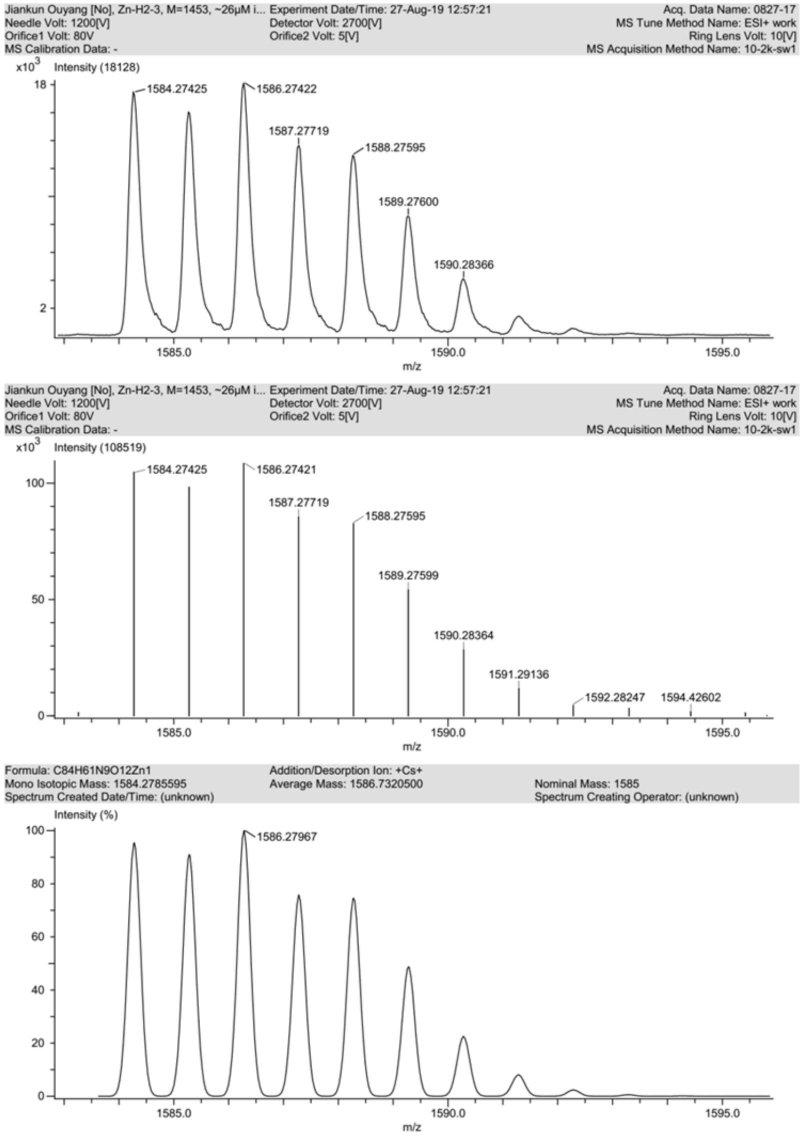

Supplementary Figure 8 HRMS-ESI spectrum of zinc S_p-(R,S)-**3.**

IR, UV-Vis, CD, and fluorescence spectra

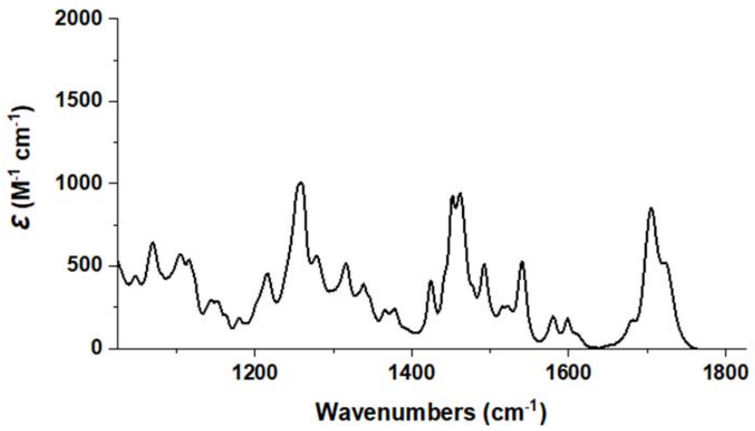

Supplementary Figure 9 Part of the IR spectrum of zinc S_p-(*R*,*S*)-**3**. [c] = 2.6 × 10^{-4} M in $CDCl_3$.

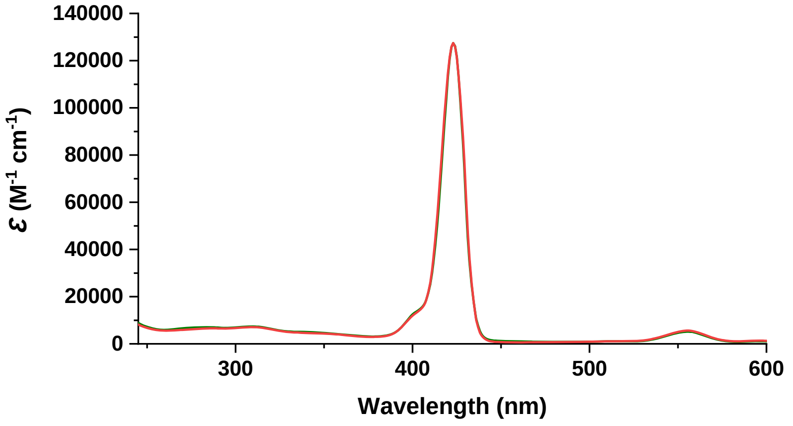

Supplementary Figure 10 UV-Vis spectrum of zinc S_p-(*R*,*S*)-**3** (green line) and zinc R_p-(*S*,*R*)-**3** (red line), [c] = 2 × 10^{-5} M in $CHCl_3$.

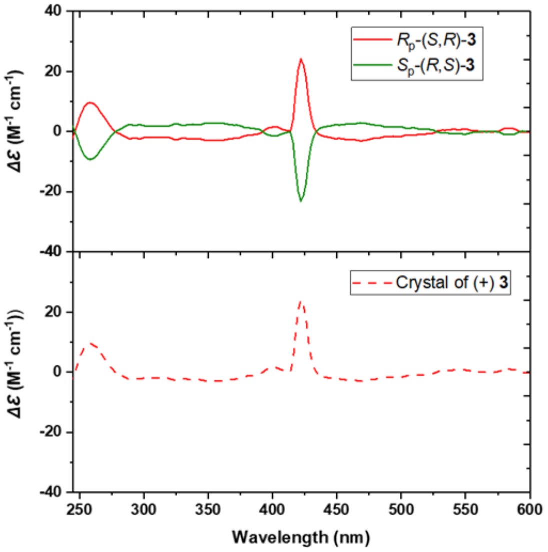

Supplementary Figure 11 CD spectra of zinc S_p-(*R*,*S*)-**3** (green line), of zinc R_p-(*S*,*R*)-**3** (red line), and of a dissolved crystal of (+)-**3**; [c] = 2×10^{-5} M in $CHCl_3$.

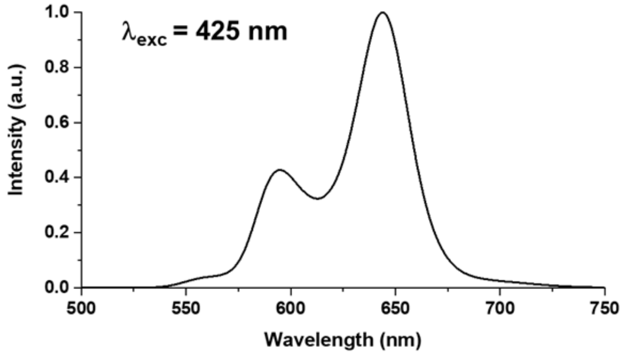

Supplementary Figure 12. Fluorescence spectrum of zinc S_p-(*R*,*S*)-**3** at 298 K in $CHCl_3$.

Porphyrin cage 4

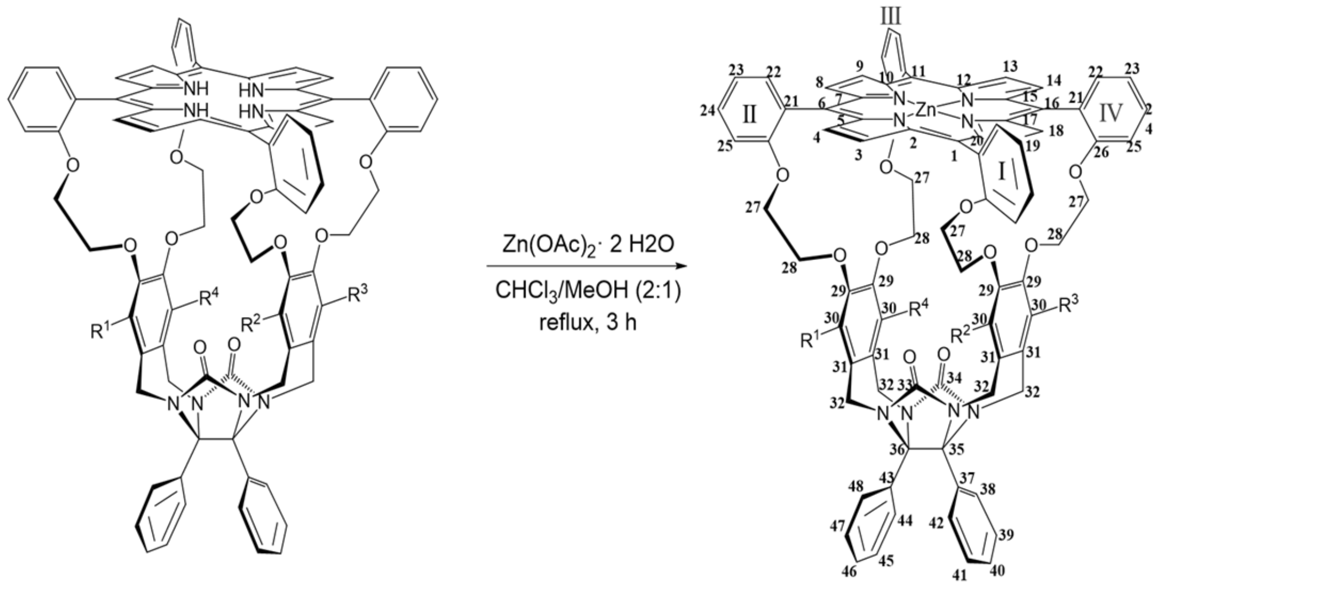

$S_p(R,R)$-**2** $R^1,R^3 = NO_2$; $R^2,R^4 = H$
$R_p(S,S)$-**2** $R^1,R^3 = H$; $R^2,R^4 = NO_2$

zinc $S_p(R,R)$-**4** $R^1,R^3 = NO_2$; $R^2,R^4 = H$
zinc $R_p(S,S)$-**4** $R^1,R^3 = H$; $R^2,R^4 = NO_2$

A flask was charged with one of the enantiomers of anti-dinitro porphyrin cage (S_p)-(R,R)-**2** or **(**R_p)-(S,S)-**2**) (10 mg, 0.007 mmol) in a degassed mixture of chloroform (2 mL) and methanol (1 mL). Zinc acetate dihydrate (6.5 mg, 0.035 mmol) was added, the mixture was excluded from light, and refluxed under argon for 3 h. After cooling, the solvent was evaporated and the residue was dissolved in dichloromethane (10 mL). The organic layer was washed with water (2×) and concentrated in vacuo. The residue was purified by column chromatography (chloroform/acetonitrile, 15:1 (v/v)). Yield: 9.8 mg (95%) of S_p-(R,R)-**4** and 9.9 mg (95%) of R_p-(S,S)-**4**) as purple solids. Melting point of the enantiomers: > 300 ℃. Optical rotations could not be measured due to the intense purple color of the enantiomers.

NMR

The NMR spectra of the two enantiomers are identical. Below, the spectral data of zinc S_p-(R,R)-**4** are shown.

^{1}H-NMR (500 MHz, Chloroform-d) δ 8.95 (d, 2H, ß-pyrrole-H-**3,4**, *J* = 5.0 Hz), 8.84 (d, 2H, β-pyrrole-H-**13**, **14** *J* = 5.0 Hz), 8.74 (d, 2H, β-pyrrole-H-**8**,**9**), 8.71 (d, 2H, ß-pyrrole-H-**18**,**19**), 8.39 (dd, 2H, ArH-**22**(**II**, **IV**), *J* = 7.3 Hz, 1.7 Hz), 8.00 (dd, 2H, ArH-**22**(**I**, **III**), *J* = 7.3 Hz, 1.8 Hz), 7.77 (td, 4H, ArH-**24**, *J* = 7.6, 7.8, 1.5 Hz), 7.49 (td, 2H, ArH-**23**(**II**, **IV**), *J* = 7.6, 7.8, 1.5 Hz), 7.37-7.33 (m, ArH-23(**I**, **III**), ArH-**25**(**I**, **III**), 4H), 7.24 (d, 2H, ArH-**25**(**II**,**IV**), *J* = 10.0Hz), 7.09 (t, 2H, ArH-**41**, **45**, *J* = 6.0Hz), 6.99 (t, 2H, ArH-**40**, **46**, *J* = 7.4Hz), 6.87-6.84 (m, 4H, ArH-**42**, **44**, ArH-**39**, **47**), 6.60 (d, 2H, ArH-**38**, **48**, *J* = 7.4Hz), 6.21 (s, ArH-**30**(**I**,**III**) 2H), 4.41-4.35 (m, 4H, CH_2-**32a**(**I**, **III**), CH_2-**27a**(**I**, **III**)), 4.27 (d, 2H, CH_2-**32a**(**II**, **IV**), *J* = 16.9Hz), 4.14 (d, 2H, CH_2-**27b**(**I**, **III**), *J* = 9.2Hz), 4.07-3.99 (m, 4H, CH_2-**27**(**II**, **IV**)), 3.80 (d, 2H, CH_2-**32b**(**I**, **III**), *J* = 15.7Hz), 3.69(d, 2H, CH_2-**32b**(**II**, **IV**), *J* = 16.9Hz), 3.63 (d, 2H, CH_2-**28a**(**I**, **III**), *J* = 15.7Hz), 3.41 (t, 2H, CH_2-**28b**(**I**, **III**), *J* = 9.8Hz), 3.29-3.24 (m, CH_2-**28a**(**II**, **IV**)), 2H), 2.37-2.34 (m, CH_2-**28b**(**II**, **IV**)), 2H).

^{13}C-NMR (126 MHz, Chloroform-d) δ 158.88 (ArC-**26**(**II**, **IV**)), 158.47 (ArC-**26**(**I**, **III**)), 156.30 (C=O-**33**, **34**), 150.52 (β-pyrrole-C-**7**, **17**), 150.47 (ArC-**29**(**I**,**III**)), 150.00 (β-pyrrole-C-**2**, **12**), 149.89 (β-pyrrole-C-**5**, **15**), 149.71 (β-pyrrole-C-**9**, **19**), 145.91 (ArC-**30**(**II**, **IV**)), 138.19 (ArC-**29**(**II**, **IV**)), 135.67 (ArC-**22**(**I**, **III**)), 134.10 (ArC-**22**(**II**, **IV**)), 133.65 (ArC-**31**(**I**, **III**)), 131.24 ((β-pyrrole-C-**9**, **19**), 131.18 ((β-pyrrole-C-**8**, **18**), 131.08 ((β-pyrrole-C-**4**, **14**), 130.89 (β-pyrrole-C-**3**, **13**), H129.73 (ArC-**24**(**I**, **III**)), 129.43 (ArC-**24**(**II**, **IV**)), 129.10 (**40**, **46**), 128.96 (**39**, **47**), 128.66 (**41**, **45**), 128.20 (**38**, **48**), 127.14 (**42**, **44**), 120.43 (ArC-**31**(**II**, **IV**)), 120.41 (ArC-**31**(**II**, **IV**)), 120.12 (ArC-**23**(**II**, **IV**)), 119.64 (ArC-**23**(**I**, **III**)), 116.04 (ArC-**6, 16**), 115.97 (ArC-**1, 11**), 114.22 (ArC-**30**(**I**, **III**)), 112.11 (ArC-**25**(**II**, **IV**)), 110.67 (ArC-**25**(**I**, **III**)), 84.64 (C-**35**, **36**), 71.61 (C-**28**(**II**, **IV**)), 67.29 (C-**28**(**I**, **III**)), 66.56 (C-**27**(**I**, **III**)), 65. 14 (C-**27**(**II**, **IV**)), 44.41 (C-**32**(**I**, **III**)), 38.95 (C-**32**(**II**, **IV**)).

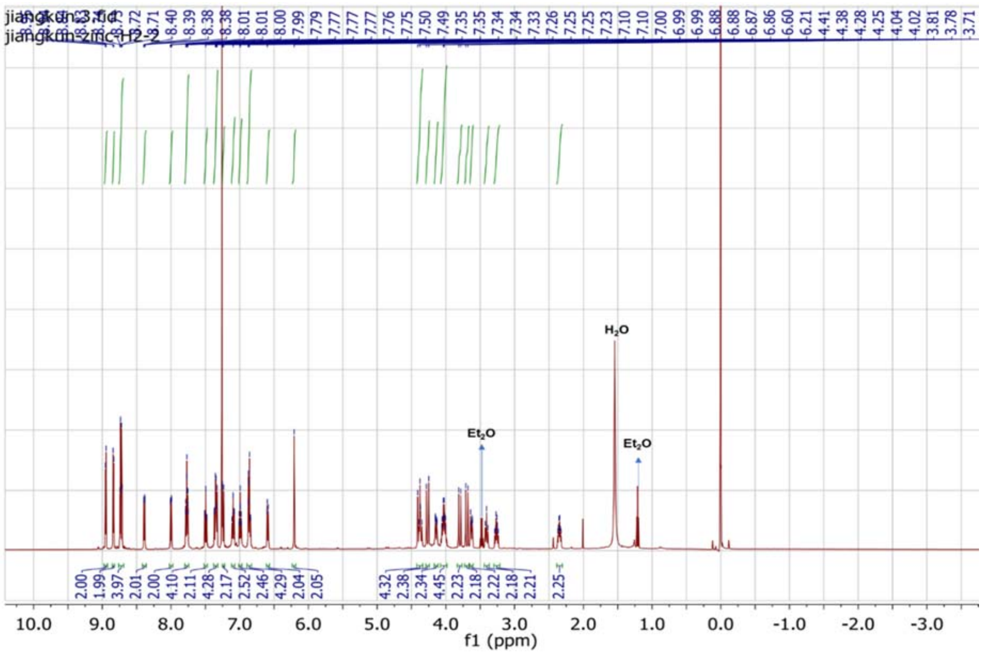

Supplementary Figure 13. ^{1}H NMR spectrum (500 MHz, 298K) of zinc S_p-(R,R)-**4** in $CDCl_3$.

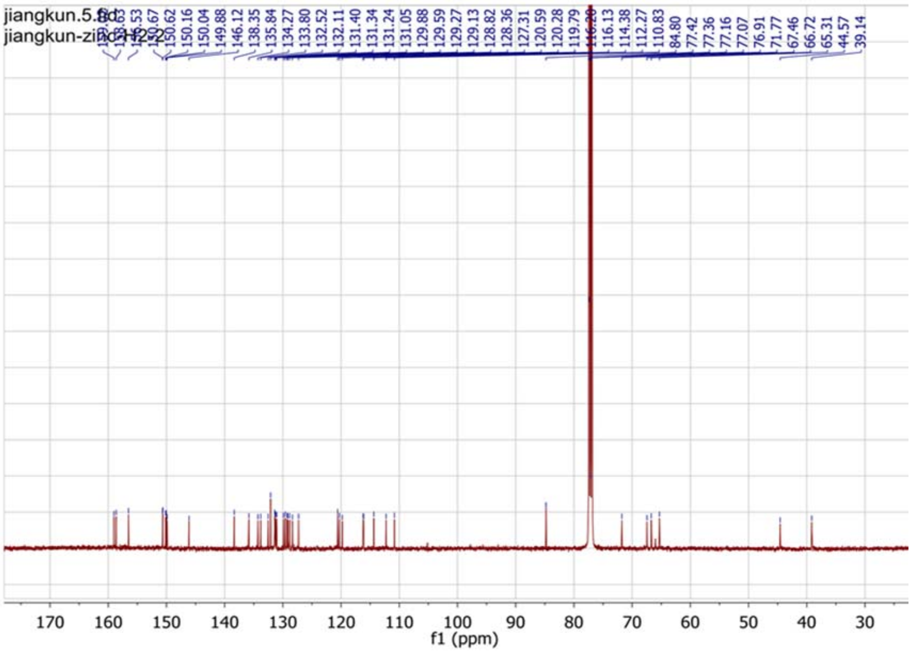

Supplementary Figure 14. ^{13}C NMR spectrum (126 MHz, 298K) of zinc S_p-(R,R)-**4** in $CDCl_3$.

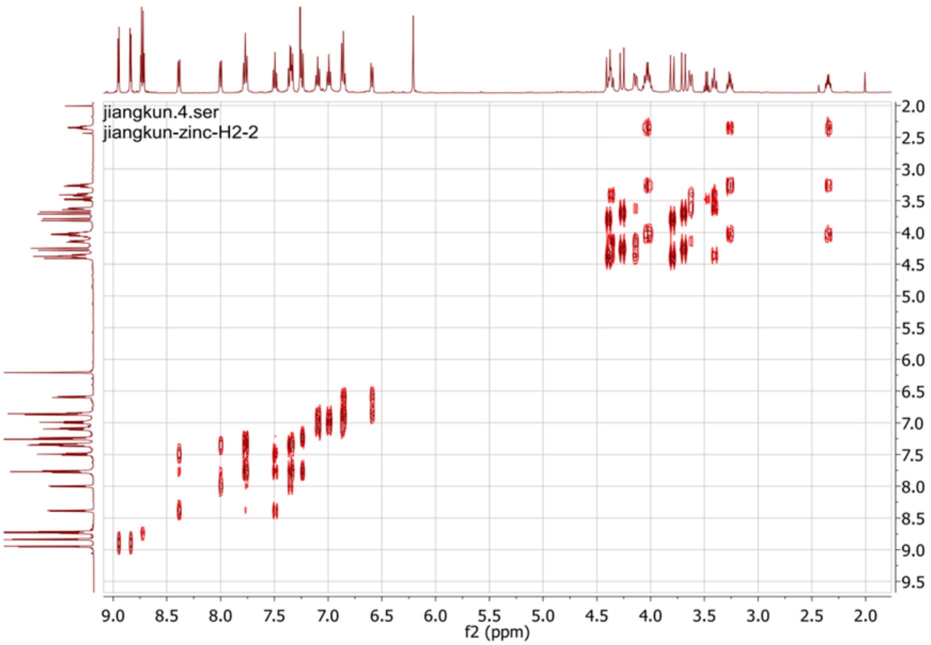

Supplementary Figure 15. ^{1}H-^{1}H COSY NMR spectrum (500 MHz, 298 K) of zinc S_p-(R,R)-**4** in $CDCl_3$.

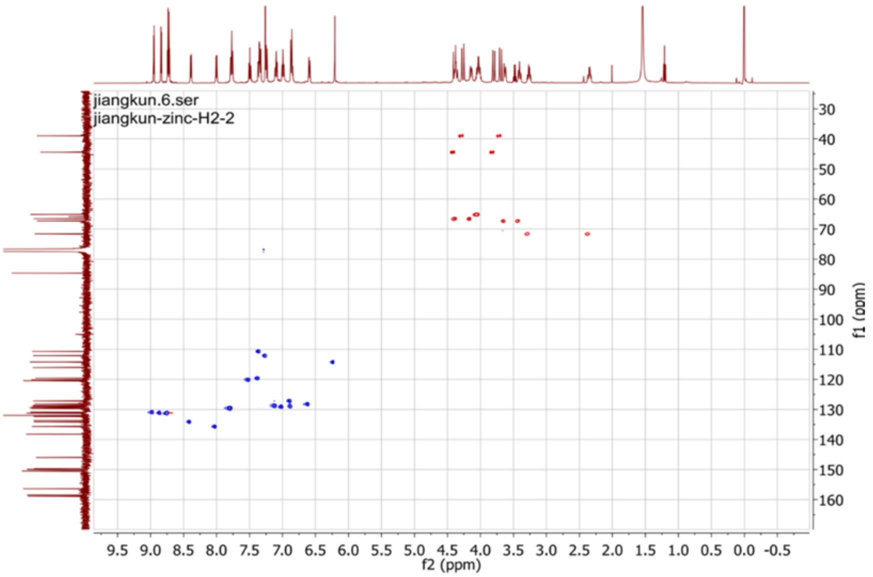

Supplementary Figure 16. ^{1}H-^{13}C edited HSQC NMR spectrum (500 MHz, 298 K) of zinc S_p-(R,R)-**4** in $CDCl_3$. CH_2 groups are indicated in red and CH/CH_3 groups in blue.

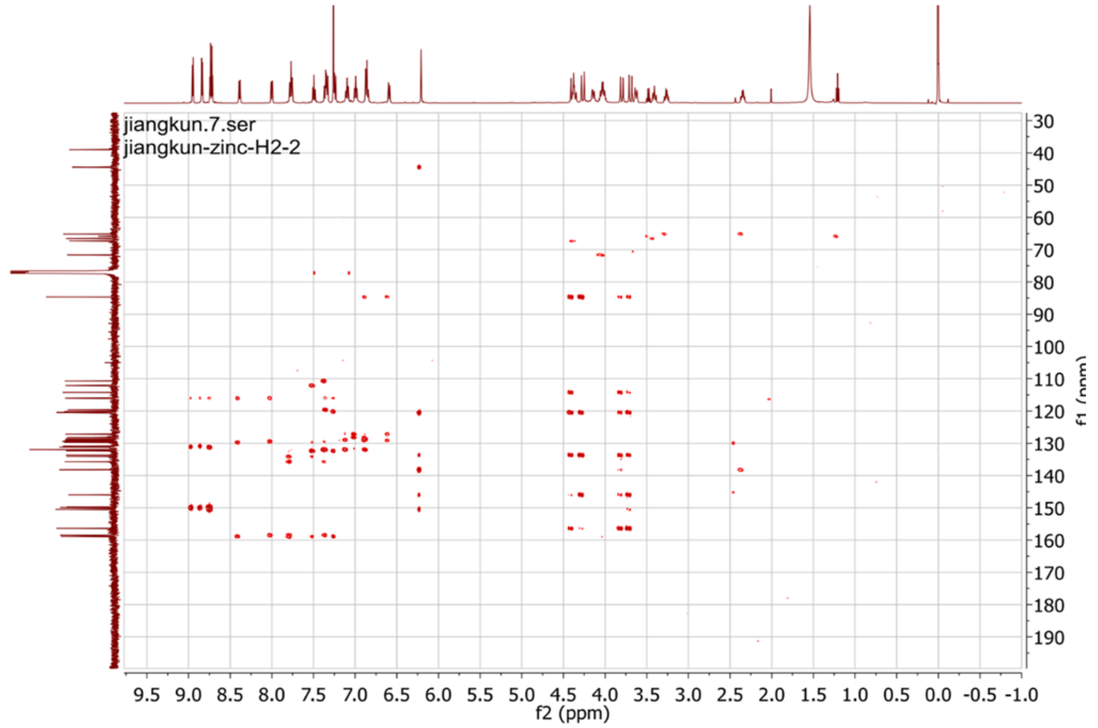

Supplementary Figure 17. ^{1}H-^{13}C HMBC NMR spectrum (500 MHz, 298 K) of zinc S_p-(R,R)-**4** in CDCl3.

MS

MALDI-TOF MASS: m/z: 1497.606 $(M+H)^+$, calculated for $C_{84}H_{60}N_{10}O_{14}Zn + H^+$ m/z: 1497.83 (the masses of the two enantiomers are identical).

a

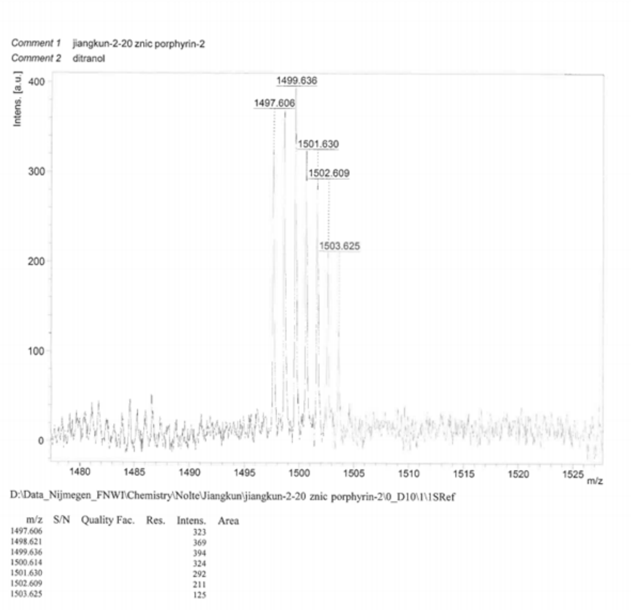

b

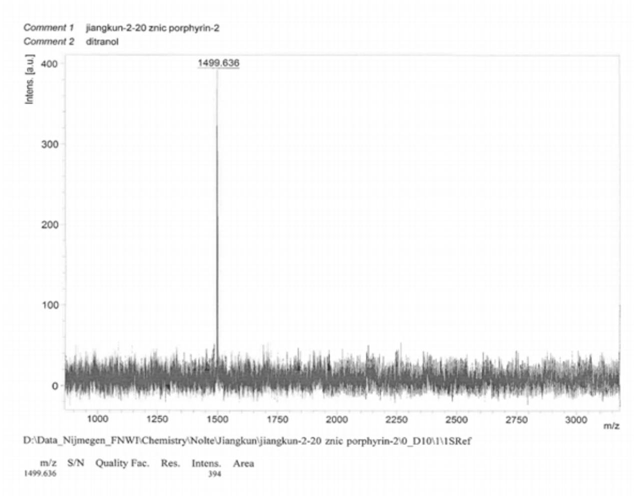

Supplementary Figure 18. MALDI-TOF MASS spectra of zinc S_p-(R,R)-**4**. **a** Magnification. **b** Full spectrum.

For the HRMS-ESI study, again a solution containing CsI was used (see above). The detected signal is [M + Cs^+]. HRMS-ESI: m/z: 1629.2614.2742 $(M + Cs)^+$ calculated for $C_{84}H_{60}CsN_{10}O_{14}Zn$ 1629.26363.

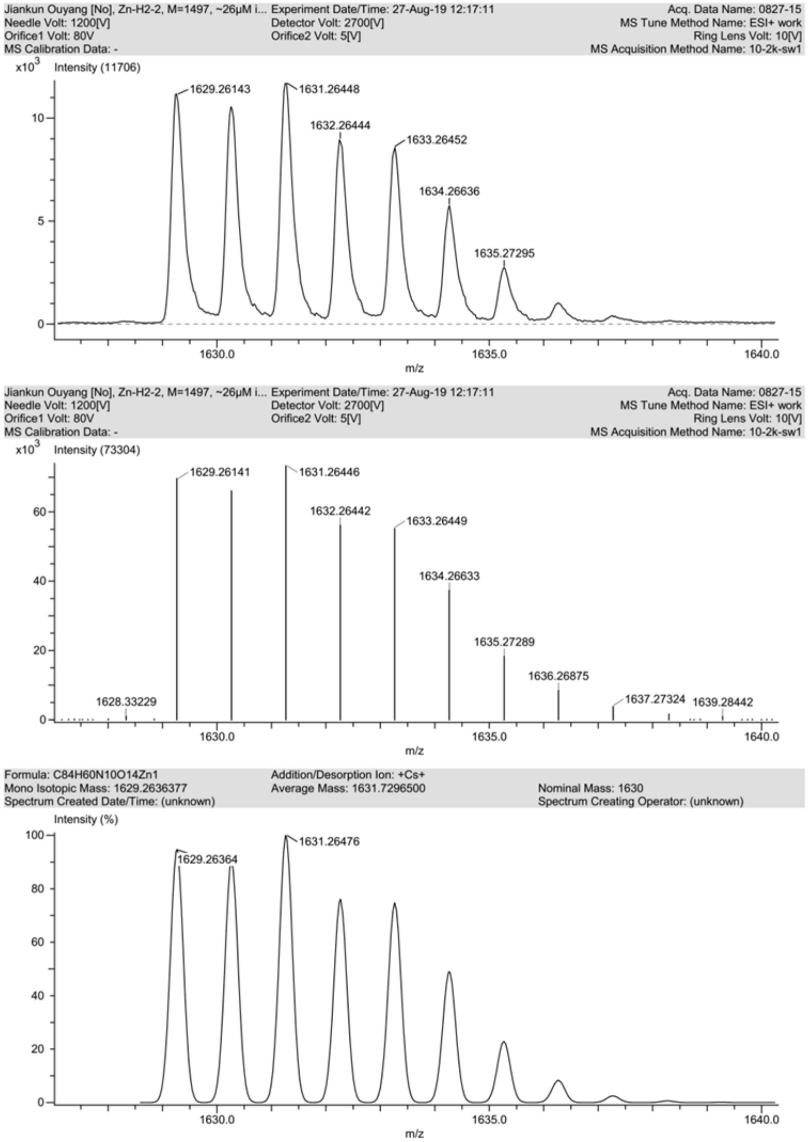

Supplementary Figure 19. HRMS-ESI spectrum of zinc S_p-(R,R)-**4.**

IR, UV-Vis, CD, and fluorescence spectra

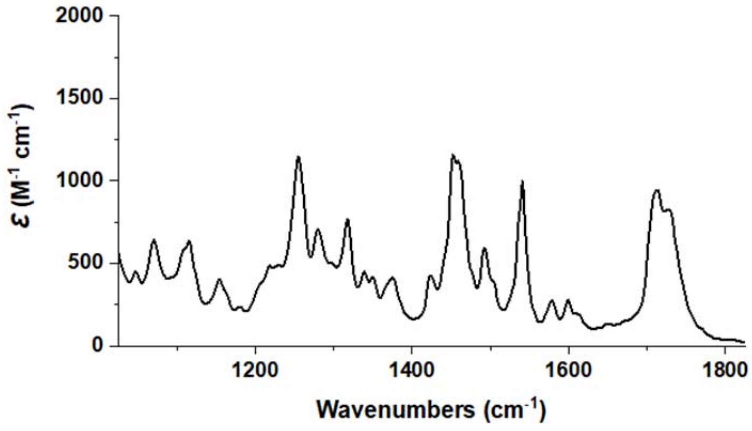

Supplementary Figure 20. IR spectrum of zinc S_p-(*R*,*R*)-**4**. [c] = 2.6 × 10^{-4} M in $CDCl_3$.

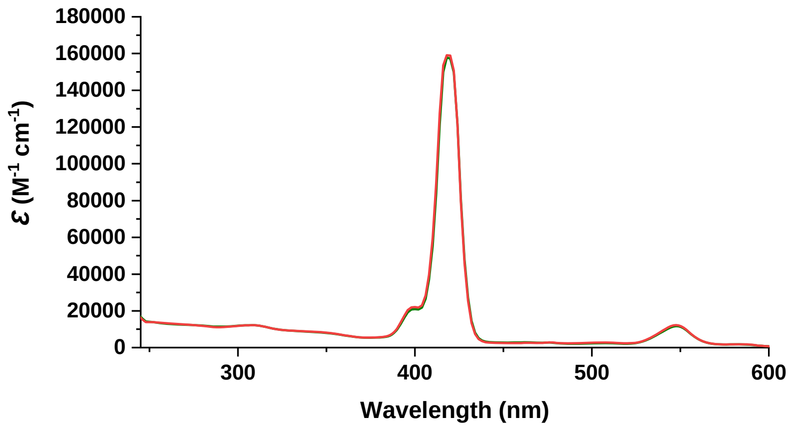

Supplementary Figure 21. UV-Vis spectra of zinc S_p-(*R*,*R*)-**4** (green line) and zinc R_p-(*S*,S)-**4** (red line), [c] = 2 × 10^{-5} M in $CHCl_3$.

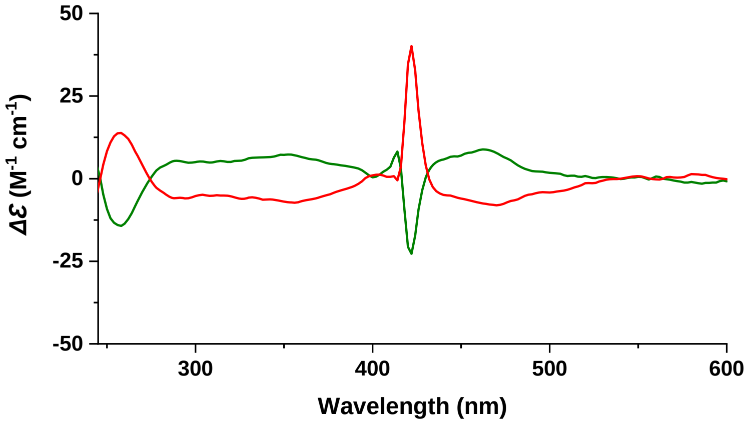

Supplementary Figure 22. CD spectra of zinc S_p-(R,R)-**4** (green line) and zinc R_p-(S,S)-**4** (red line), [c] = 2×10^{-5} M in $CHCl_3$.

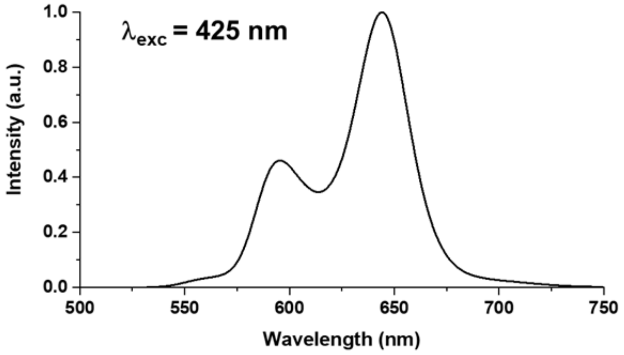

Supplementary Figure 23. Fluorescence spectrum of zinc S_p-(R,R)-**4** at 298 K in $CHCl_3$.

Synthesis of guest compounds

Guests **5** and **6** were synthesized according to literature procedures.[6-8]

Racemization studies

To study the possible racemization of the planar chiral porphyrin cages, chiral HPLC was applied. Upon injection of racemic **1** into the column, two peaks of equal area were observed (Supplementary Figure 24b). The first fraction of **1** was collected and again injected into the chiral HPLC. Only one peak was observed (Supplementary Figure 24c). Then, this solution was kept at 40 ^{o}C for 18 h, and re-injected onto the chiral HPLC. The second peak was not visible (Supplementary Figure 24d), indicating that the first fraction of **1** had not racemized and was chemically stable under this condition. For racemic **2**, a similar procedure was followed (Supplementary Figure 25). After heating, no second peak appeared, meaning that also the enantiomers of **2** are chemically stable.

Experimental procedure: the sample was dissolved in dichloromethane and injected into the chiral column. Detection was performed by UV (λ 254 nm). The flow-rate was set at 1 mL/min.

Column	Mobile Phase for 1	t1	k1	t2	k2	a	Rs
Chiralpak IA	Hexane/DCM/EtOH = 30/50/20 (v/v/v)	8.36	1.89	12.3	3.25	1.71	2.62

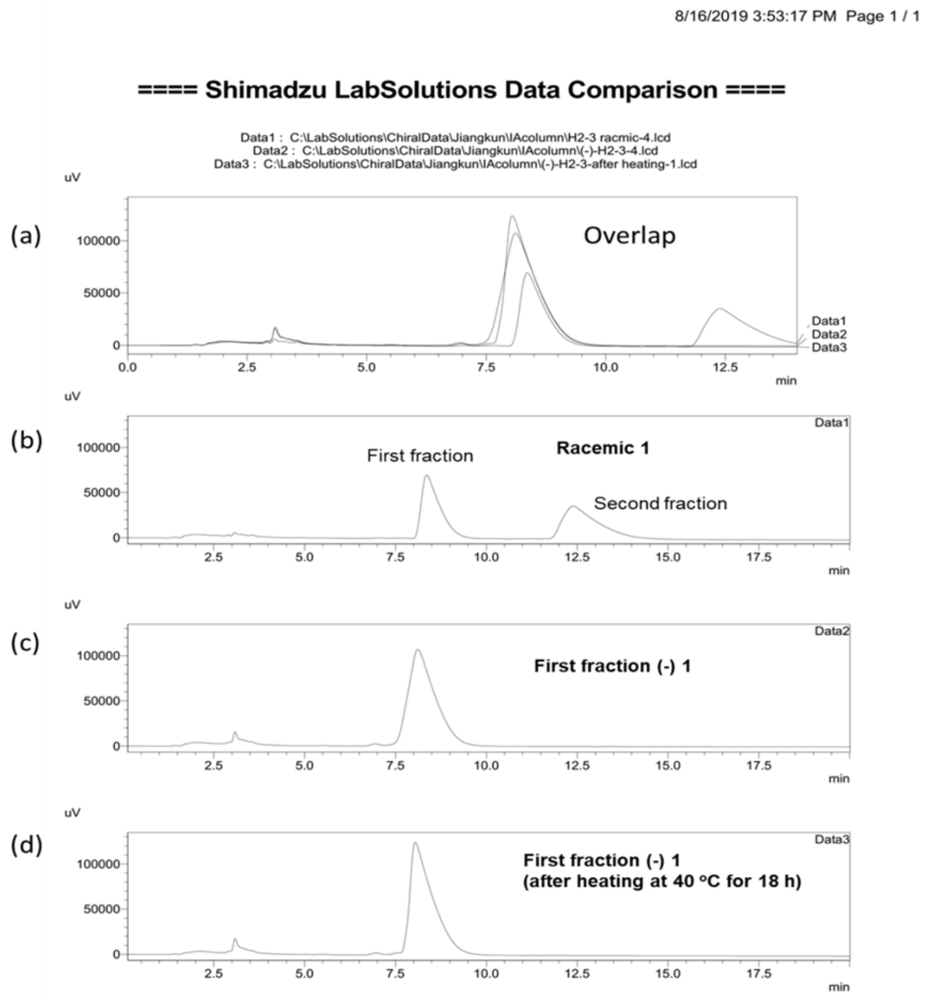

Supplementary Figure 24. (a) Overlapped HPLC traces of **1**. (b) Chiral HPLC trace of racemic **1**, (c) Chiral HPLC trace of the first collected fraction of **1**. (d) Idem, after refluxing the solution at 40 °C for 18 h. Eluent hexane/DCM/EtOH = 30/50/20 (v/v/v).

Column	Mobile Phase for 2	t1	k1	t2	k2	a	Rs
Chiralpak IA	Hexane/DCM/EtOH = 30/60/10 (vol %)	3.77	0.30	11.48	2.97	9.90	12.18

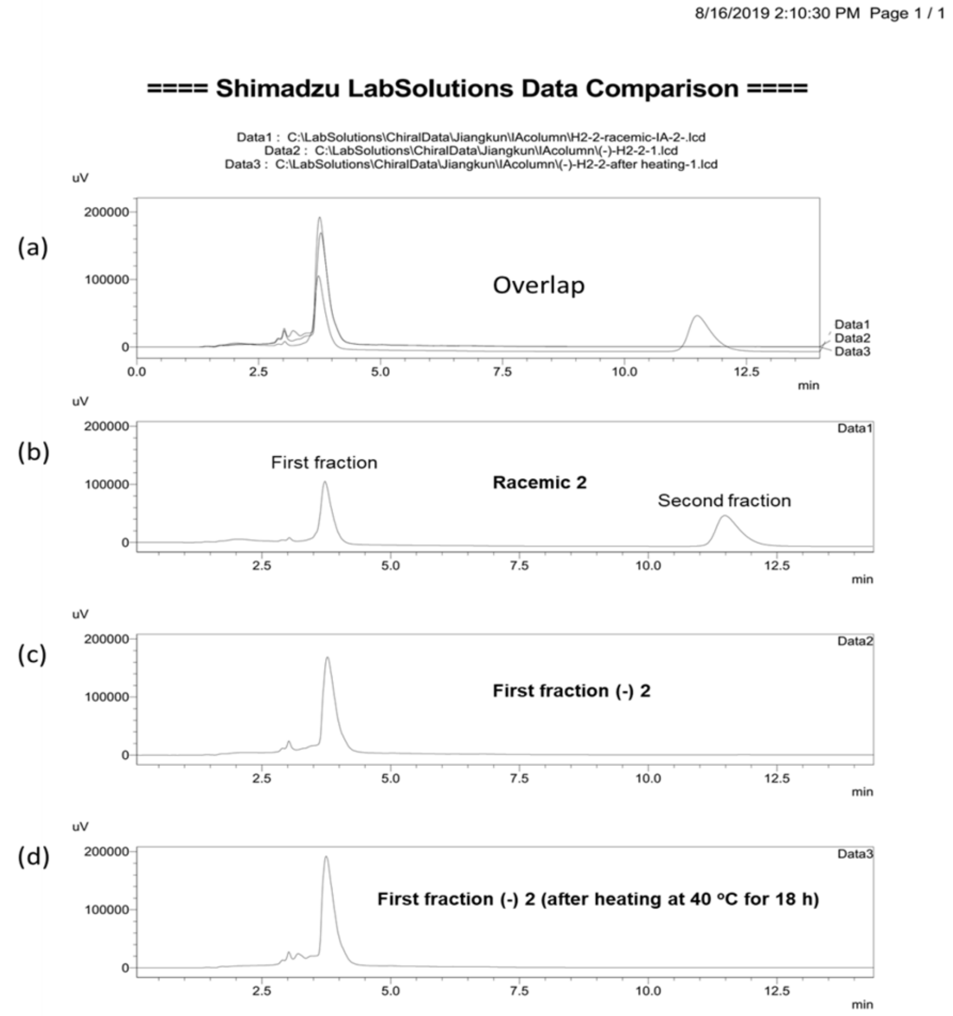

Supplementary Figure 25. (a) Overlapped HPLC traces of **2**. (b) Chiral HPLC trace of racemic **2**, (c) Chiral HPLC trace of the first collected fraction of **2**. (d) Idem, after refluxing the solution at 40 °C for 18 h. Eluent hexane/DCM/ EtOH = 30/60/10 (v/v/v).

ECD and UV-Vis spectra

General experimental procedures

A chiral porphyrin cage compound (H, 0.75 μmol ≈ 1.04-1.08 mg) was dissolved in 25.00 mL of distilled acetonitrile, and the sample was agitated by ultrasonication to dissolve all solid material, resulting in a pink solution. The stoppered flask was then inverted several times to obtain a homogenous host solution ([H] ≈ 30 μM). Viologen guests (G, 4.5 μmol ≈ 1.99-3.28 mg) were dissolved in 5.00 mL of distilled acetonitrile to obtain homogenous guest solutions ([G] ≈ 900 μM). A baseline of the empty cuvette holder, without any cuvette or sample present, was measured. Blanc spectra were using cuvettes filled with distilled acetonitrile. A titration was then carried out by first measuring 500 μL of host solution without any guest present. Guest solution was then added to obtain solutions with a total of 4, 10, 20, 50, 80, and 130 μL of guest solution and 500 μL of host solution (yielding [G]/[H]-ratios between 0.25-8). In the case of the mixed solvent system acetonitrile:chloroform = 1:1 (v/v) a titration was carried out by first measuring 500 μL of host solution without any guest present. Guest solution was then added to obtain solutions with a total of 10, 20, 80 μL of guest solution and 500 μL of host solution (yielding [G]/[H]-ratios between 0.6-4.5). After each series, the measured solution was stored to recover the host and the cuvette was flushed with distilled acetonitrile three times. When measuring a new host solution, the cuvette was flushed an additional three times with host solution and then filled with 500 μL of host solution to start a new series of measurements.[8] At the end of the experiments, reference spectra for the chiral guests were measured by mixing 400 μL of distilled acetonitrile with 300 μL of guest solution.[9] ECD- and UV-vis measurements were performed at the same time using a Jasco J-815 CD-spectrometer.

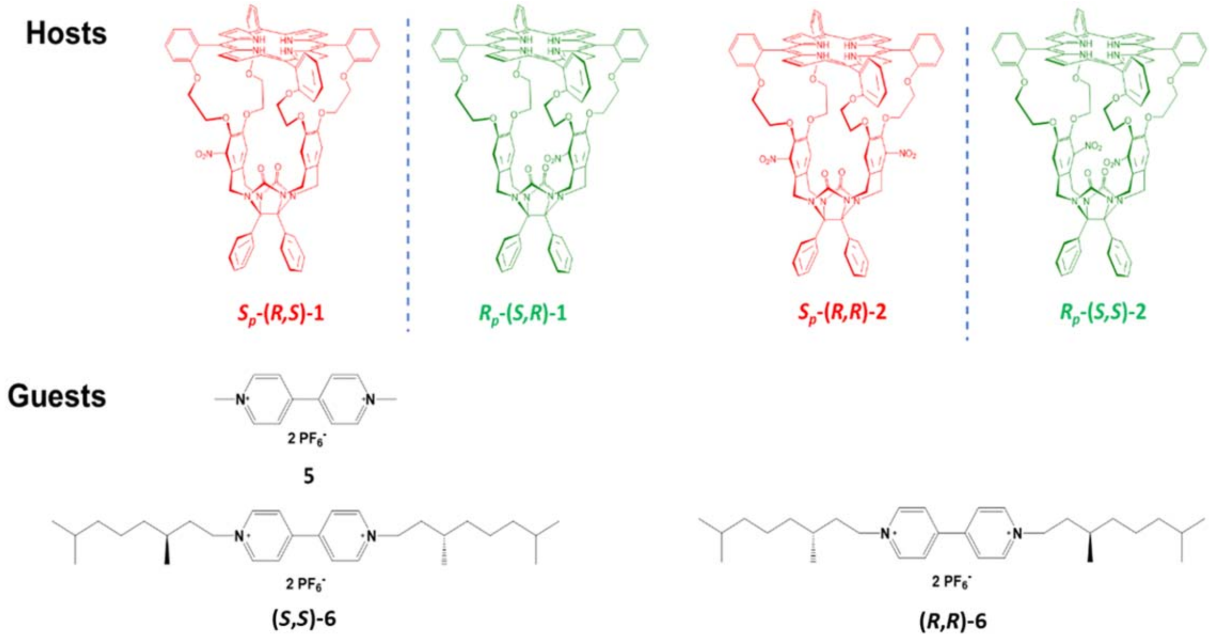

Supplementary Figure 26. Molecular structures of hosts **1-2** and guests **5** and **6**.

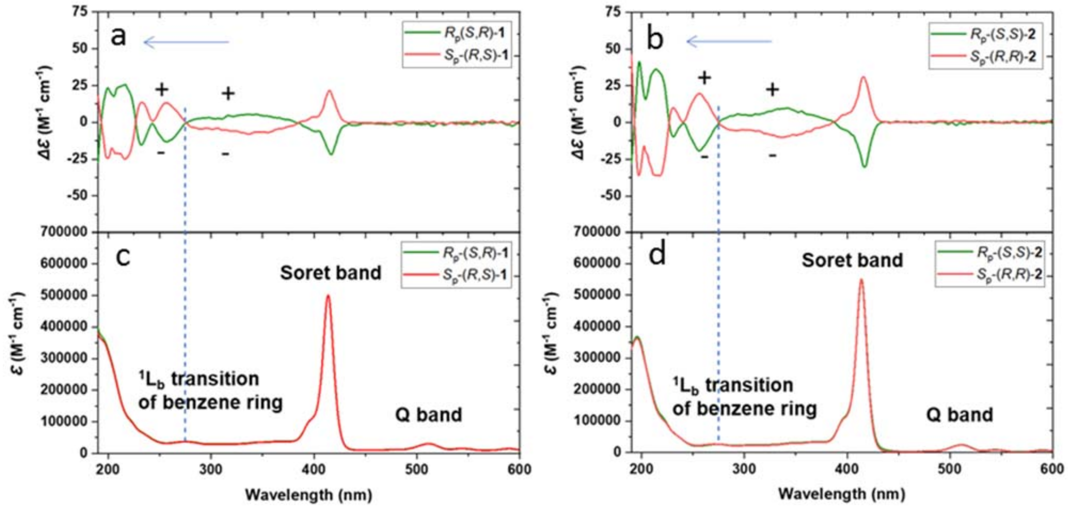

Supplementary Figure 27. ECD and UV-Vis spectra. a ECD spectra of R_p-(S,R)-**1** and S_p-(R,S)-**1**. **b** ECD spectra of R_p-(S,S)-**2** and S_p-(R,R)-**2**. **c** UV-Vis spectra of R_p-(S,R)-**1** and S_p-(R,S)-**1**. **d** UV-Vis spectra of R_p-(S,S)-**2** and S_p-(R,R)-**2**.

Supplementary Table 1 ECD signals of R_p-(*S*,*R*)**-1,** S_p-(*R*,*S*)**-1,** R_p-(*S*,*S*)**-2** and S_p-(*R*,*R*)**-2** in acetonitrile.

	$\Delta\varepsilon$ (M^{-1}. cm^{-1})			$\Delta\varepsilon$ (M^{-1}. cm^{-1})	
Wavelength (nm)	R_p-(*S*,*R*)**-1**	S_p-(*R*,*S*)**-1**	Wavelength (nm)	R_p-(*S*,*S*)**-2**	S_p-(*R*,*R*)**-2**
508	−0.4	+0.4	508	−1.2	+1.8
415	−21.8	+21.6	415	−31.1	+31.1
335	+5.5	−7.3	339	+ 9.5	−9.8
256	−13.2	+13.2	256	−19.2	+19.8
231	−14.7	+13.4	231	−9.7	+9.9
215	+25.0	−24.4	213	+36.2	−36.0
196	+23.2	−14.1	197	+41.3	−35.6

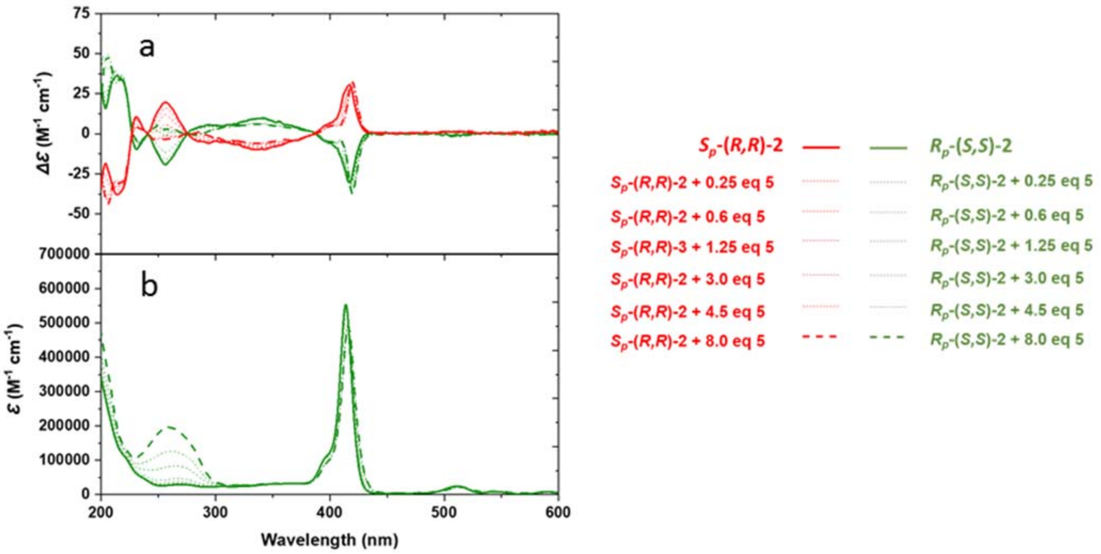

Supplementary Figure 28. ECD and UV-Vis spectra. a ECD spectra of S_p-(*R*,*R*)**-2** and R_p-(*S*,*S*)**-2** upon the addition of guest **5**. **b** UV-Vis spectra of S_p-(*R*,*R*)**-2** and R_p-(*S*,*S*)**-2** upon the addition of guest **5**. Solvent acetonitrile.

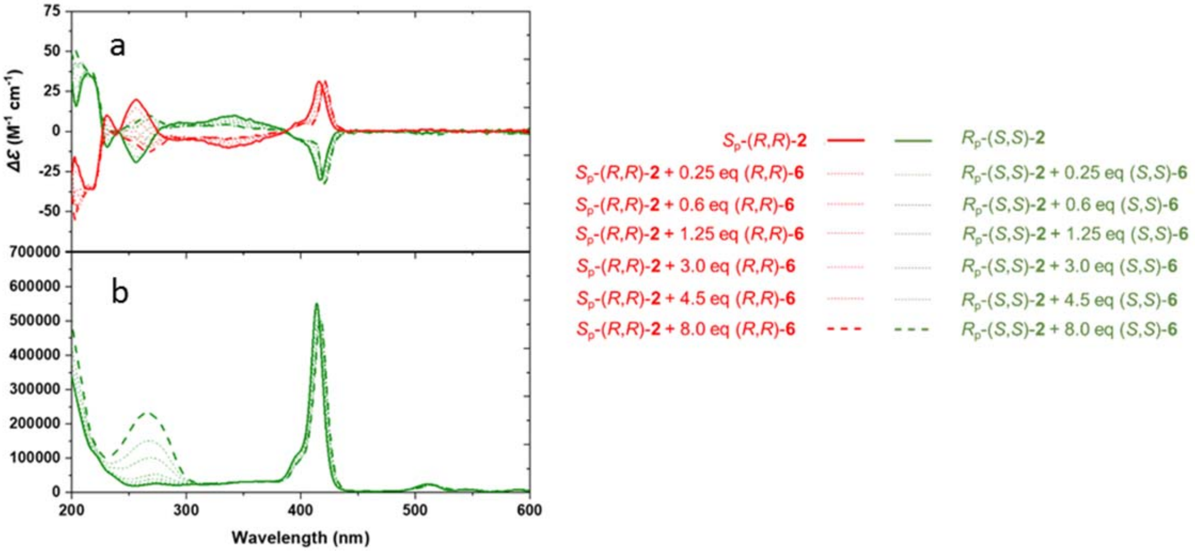

Supplementary Figure 29. ECD and UV-Vis spectra. a ECD spectra of S_p-(*R*,*R*)-**2** upon the addition of (*R*,*R*)-**6** and R_p-(*S*,*S*)-**2** upon the addition of (*S*,*S*)-**6**. **b** UV-Vis spectra of S_p-(*R*,*R*)-**2** upon the addition of (*R*,*R*)-**6** and R_p-(*S*,*S*)-**2** upon the addition of (*S*,*S*)-**6.** Solvent acetonitrile.

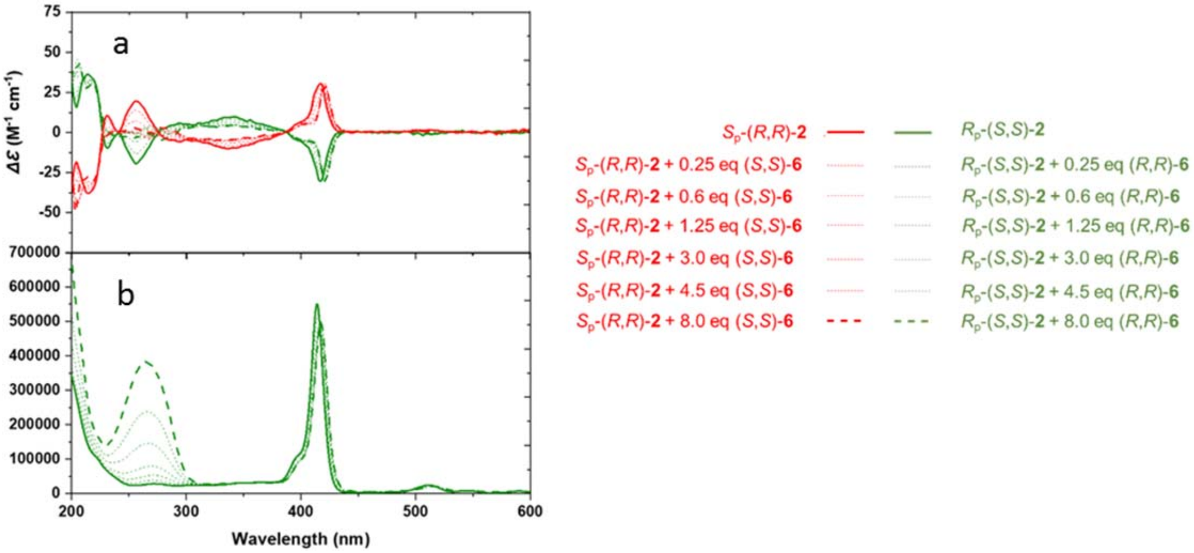

Supplementary Figure 30. ECD and UV-Vis spectra. a ECD spectra of S_p-(*R*,*R*)-**2** upon the addition of guest (*S*,*S*)-**6** and R_p-(*S*,*S*)-**2** upon the addition of guest (*R*,*R*)-**6**. **b** UV-Vis spectra of S_p-(*R*,*R*)-**2** upon the addition of guest (*S*,*S*)-**6** and R_p-(*S*,*S*)-**2** upon the addition of guest (*R*,*R*)-**6**. Solvent acetonitrile.

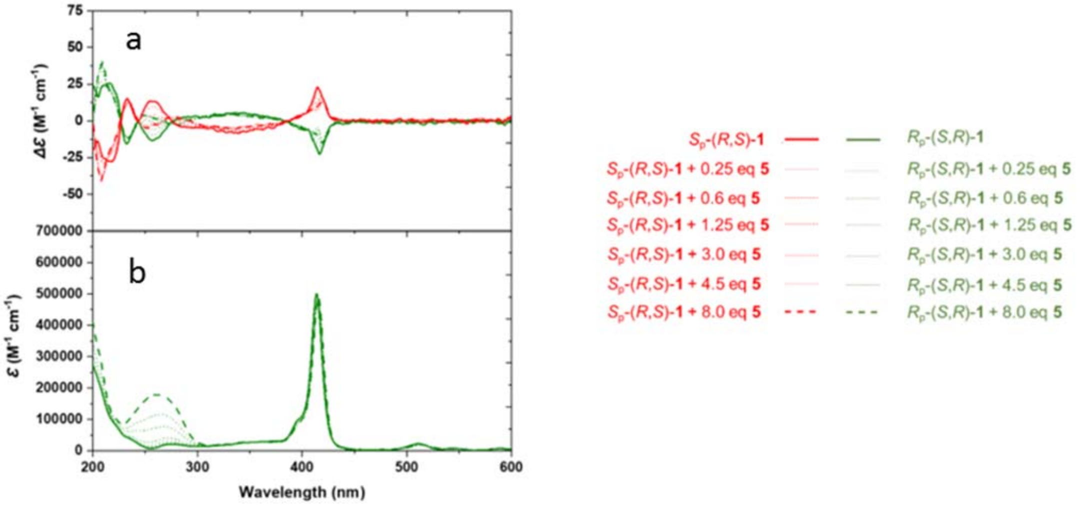

Supplementary Figure 31. ECD and UV-Vis spectra. a ECD spectra of S_p-(*R*,*S*)-**1** and R_p-(*S*,*R*)-**1** upon the addition of guest **5**. **b** UV-Vis spectra of S_p-(*R*,*S*)-**1** and R_p-(*S*,*R*)-**1** upon the addition of guest **5**. Solvent acetonitrile.

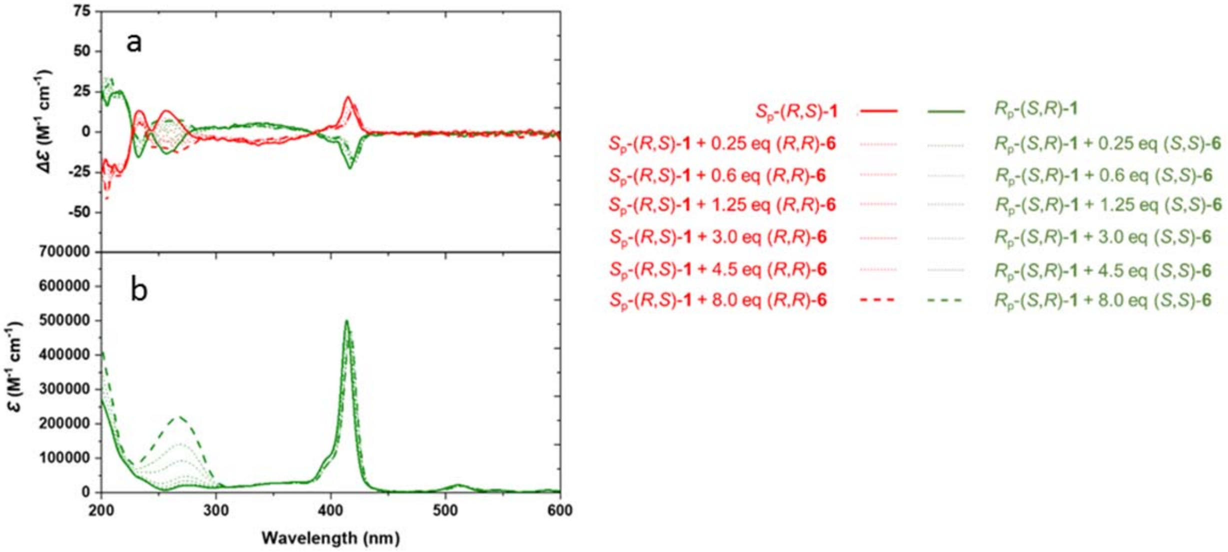

Supplementary Figure 32. ECD and UV-Vis spectra. a ECD spectra of S_p-(*R*,*S*)-**1** upon the addition of guest (*R*,*R*)-**6** and R_p-(*S*,*R*)-**1** upon addition of guest (*S*,*S*)-**6** guest. **b** UV-Vis spectra of S_p-(*R*,*S*)-**1** upon the addition of guest (*R*,*R*)-**6** and R_p-(*S*,*R*)-**1** upon addition of guest (*S*,*S*)-**6**. Solvent acetonitrile.

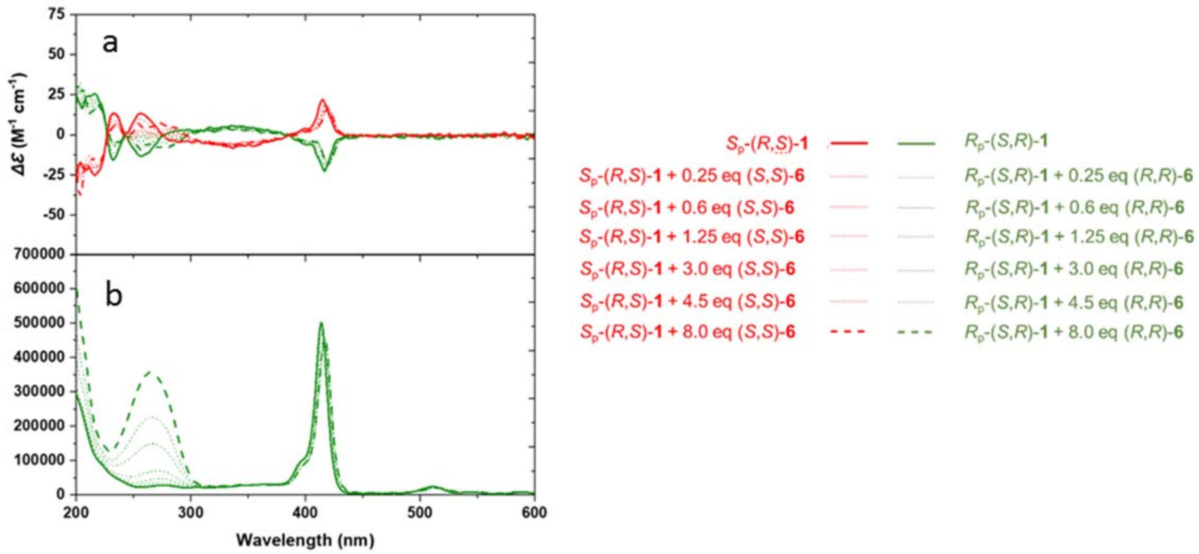

Supplementary Figure 33. ECD and UV-Vis spectra. a ECD spectra of S_p-(*R*,*S*)-**1** upon the addition of guest (*S*,*S*)-**6** and R_p-(*S*,*R*)-**1** upon the addition of guest (*R*,*R*)-**6**. **b** UV-Vis spectra of S_p-(*R*,*S*)-**1** upon the addition of guest (*S*,*S*)-**6** and R_p-(*S*,*R*)-**1** upon the addition of guest (*R*,*R*)-**6**. Solvent acetonitrile.

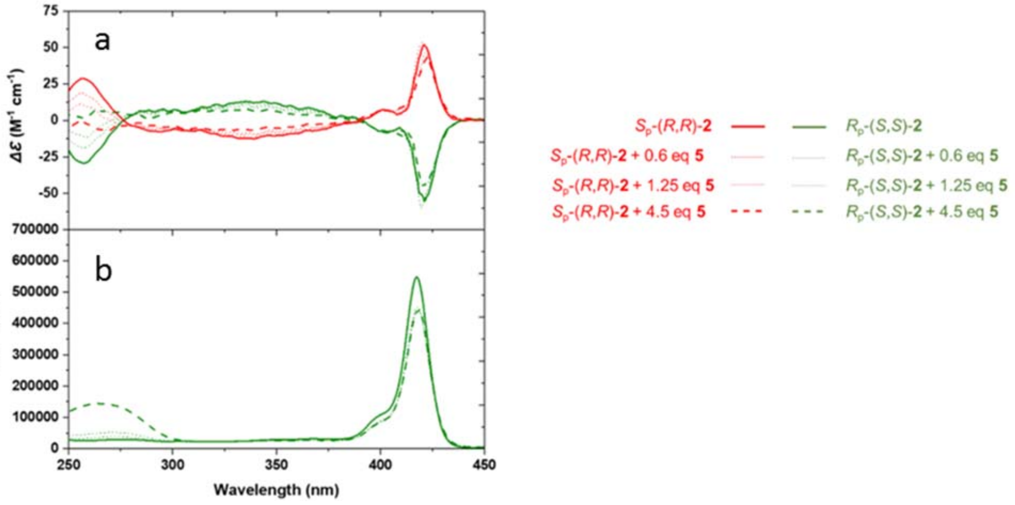

Supplementary Figure 34. ECD and UV-Vis spectra. a ECD spectra of S_p-(*R*,*R*)-**2** and R_p-(*S*,*S*)-**2** upon the addition of guest **5**. **b** UV-Vis spectra of S_p-(*R*,*R*)-**2** and R_p-(*S*,*S*)-**2** upon the addition of guest **5**. Solvent $CHCl_3$/MeCN (1:1 (v/v).

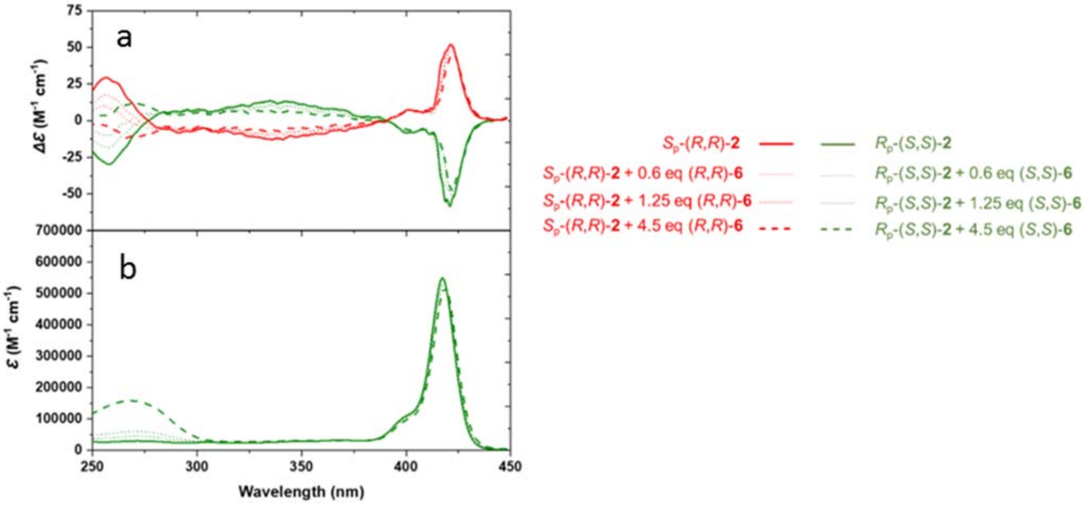

Supplementary Figure 35. ECD and UV-Vis spectra. a ECD spectra of S_p-(*R*,*R*)-**2** upon the addition of guest (*R*,*R*)-**6** and R_p-(*S*,S)-**2** upon the addition of guest (*S*,*S*)-**6**. **b** UV-Vis spectra of S_p-(*R*,*R*)-**2** upon the addition of guest (*R*,*R*)-**6** and R_p-(*S*,S)-**2** upon the addition of guest (*S*,*S*)-**6**. Solvent $CHCl_3$/MeCN (1:1 (v/v).

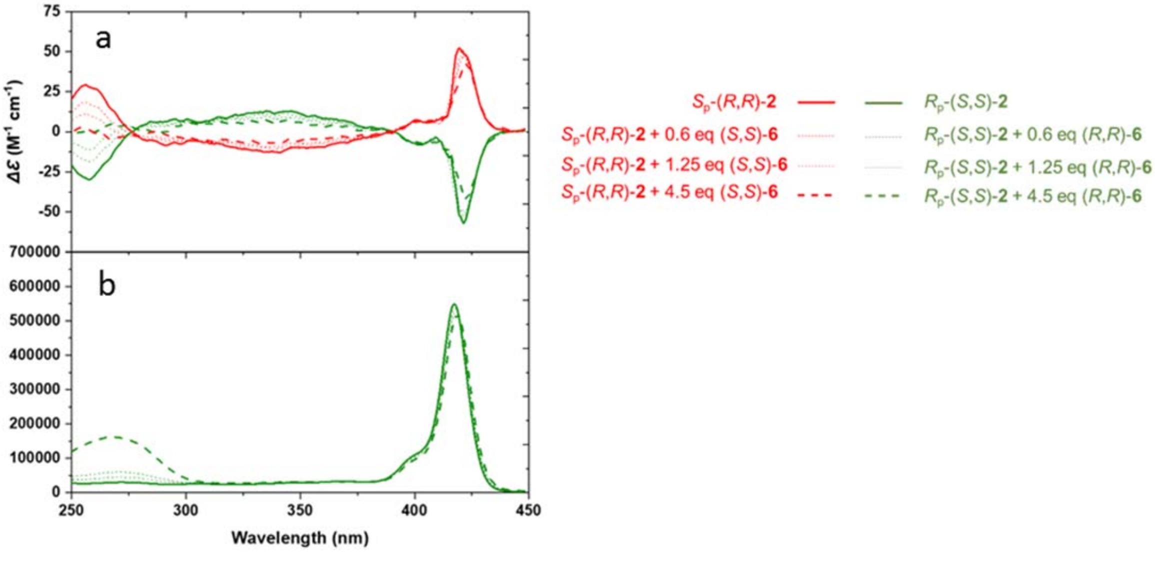

Supplementary Figure 36. ECD and UV-Vis spectra. a ECD spectra of S_p-(*R*,*R*)-**2** upon the addition of guest (*S*,*S*)-**6** and R_p-(*S*,S)-**2** upon the addition of guest (*R*,*R*)-**6**. **b** UV-Vis spectra of S_p-(*R*,*R*)-**2** upon the addition of guest (*S*,*S*)-**6** and R_p-(*S*,S)-**2** upon the addition of guest (*R*,*R*)-**6**. Solvent $CHCl_3$/MeCN (1:1 (v/v).

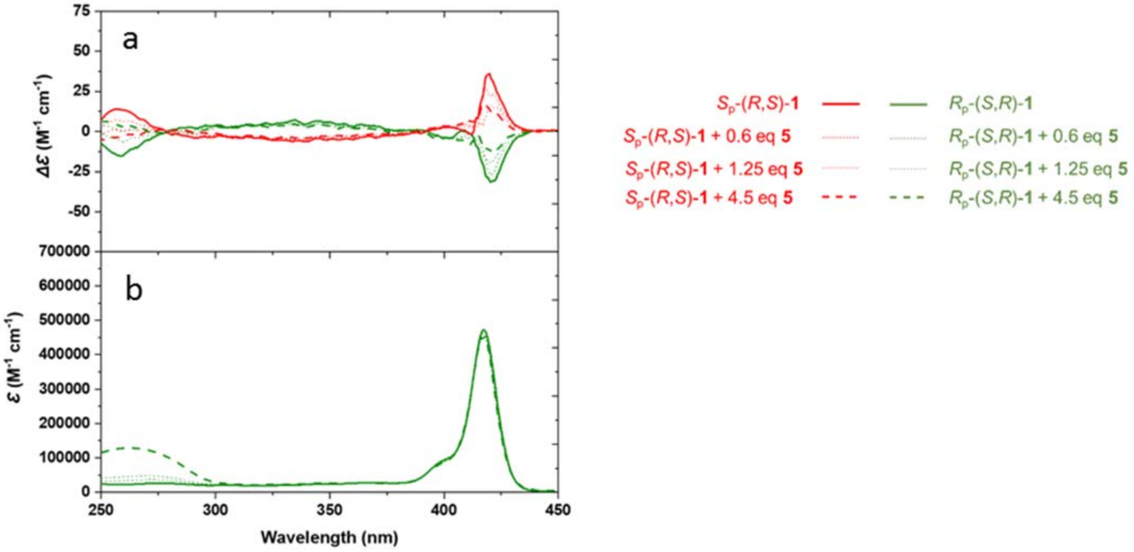

Supplementary Figure 37. ECD and UV-Vis spectra. a ECD spectra of S_p-(*R*,*S*)-**1** and R_p-(*S*,*R*)-**1** upon the addition of guest **5**. **b** UV-Vis spectra of S_p-(*R*,*S*)-**1** and R_p-(*S*,*R*)-**1** upon the addition of guest **5**. Solvent $CHCl_3$/MeCN (1:1 (v/v).

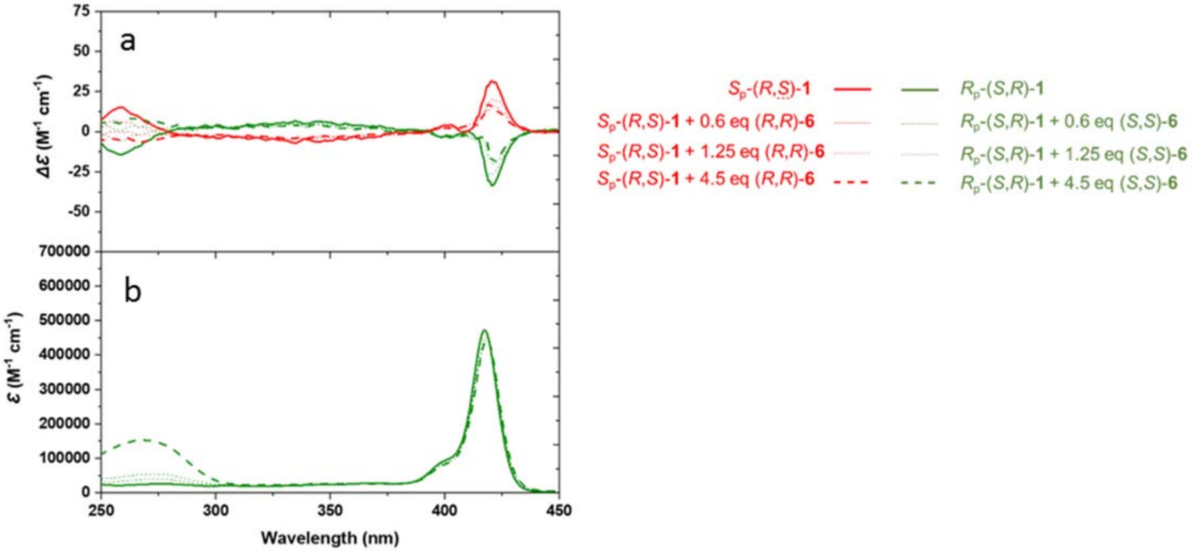

Supplementary Figure 38. ECD and UV-Vis spectra. a ECD spectra of S_p-(*R*,*S*)-**1** upon the addition of guest (*R*,*R*)-**6** and R_p-(*S*,R)-**1** upon the addition of guest (*S*,*S*)-**6** guest. **b** UV-Vis spectra of S_p-(*R*,*S*)-**1** upon the addition of guest (*R*,*R*)-**6** and R_p-(*S*,R)-**1** upon the addition of guest (*S*,*S*)-**6** guest. $CHCl_3$/MeCN (1:1 (v/v).

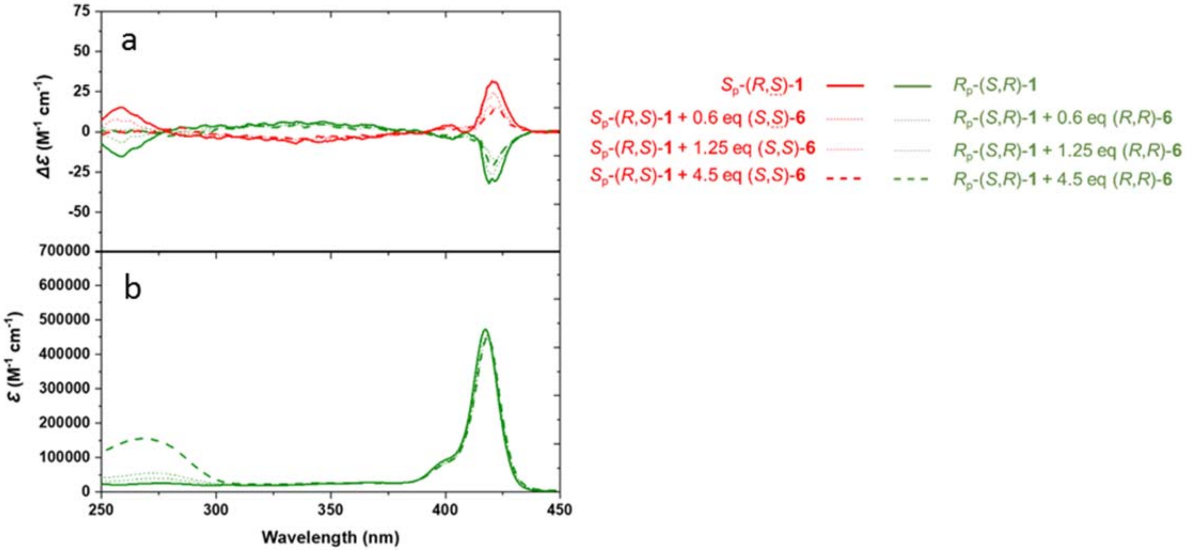

Supplementary Figure 39. ECD and UV-Vis spectra. a ECD spectra of S_p-(*R*,*S*)-**1** upon the addition of guest (*S*,*S*)-**6** and R_p-(*S*,*R*)-**1** upon the addition of guest (*R*,*R*)-**6**. **b** UV-Vis spectra of S_p-(*R*,*S*)-**1** upon the addition of guest (*S*,*S*)-**6** and R_p-(*S*,*R*)-**1** upon the addition of guest (*R*,*R*)-**6**. Solvent $CHCl_3$/MeCN (1:1 (v/v).

Fluorescence spectra

Titration of planar chiral porphyrin cage compounds with viologen guests

The experiments were carried out in a mixture of $CHCl_3$/MeCN (1:1 (v/v)) to keep both the host and guest soluble. The starting concentration of host was 1.5 µM, to which in ca. 30 steps at least 10 equivalents of guest were added from a concentrated stock solution.

Fluorescence-measurements were performed using a Jasco FP-8300 spectrophotometer, equipped with a Peltier temperature controller. The machine was allowed to warm at least half an hour before the start of the measurement. All samples were measured in quartz fluorescence cuvettes of 1×1 cm path length, with an internal volume of 3500 µL. Titrations were carried out using the *Spectral Measurement*-option, using 418 nm as the excitation wavelength and by observing the emission between 425-800 nm. The temperature was kept at 25.0 ± 0.1 °C. An excitation bandwidth of 5 nm and an emission bandwidth of 5 nm were used to obtain a good signal-noise ratio, and the scanning speed was set to 1000 nm/min.

Experimental procedure

A dry 1:1 (v/v) mixture of chloroform and acetonitrile was prepared in a volumetric flask. A host-stock solution of 50 μM was prepared by dissolving the host in the solvent mixture, which was weighed to determine the density. Three host-measurement solutions of 1.5 μM were prepared from a weighed amount of host solution (300 μL), which was diluted to a total volume of 10.00 mL. A guest-stock solution of 200 μM was prepared in 5.00 mL of the same solvent mixture. Three guest-measurement solutions were prepared, each containing 1.5 μM host and 45 μM guest, which were made by mixing weighed amounts of host-stock (150 μL) and guest-stock (1125 μL) solutions and diluted to a total volume of 5.00 mL.

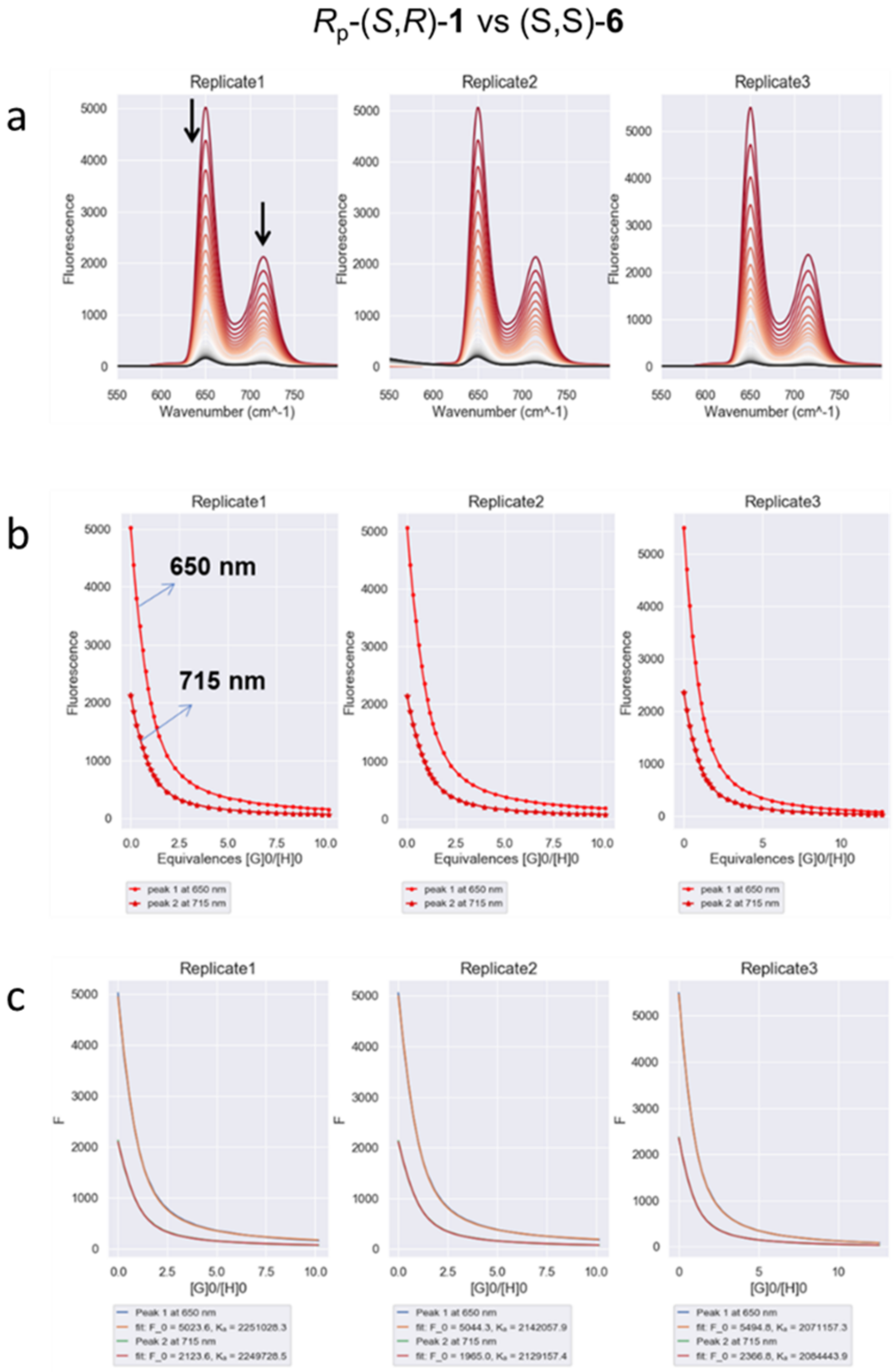

Supplementary Figure 40. Fluorescence titration experiments a Fluorescence spectra of R_p-(*S*,*R*)-**1** upon titration with (*S*,*S*)-**6** (triplo). **b** Binding isotherms of R_p-(*S*,*R*)-**1** upon titration with (*S*,*S*)-**6** at 650 and 715 nm (triplo). **c** Fitting curves (blue line at 650 nm, green line at 715 nm) and binding isotherms (orange line at 650 nm, and red line at 715 nm) (triplo).

R_p-(S,R)-**1** vs (R,R)-**6**

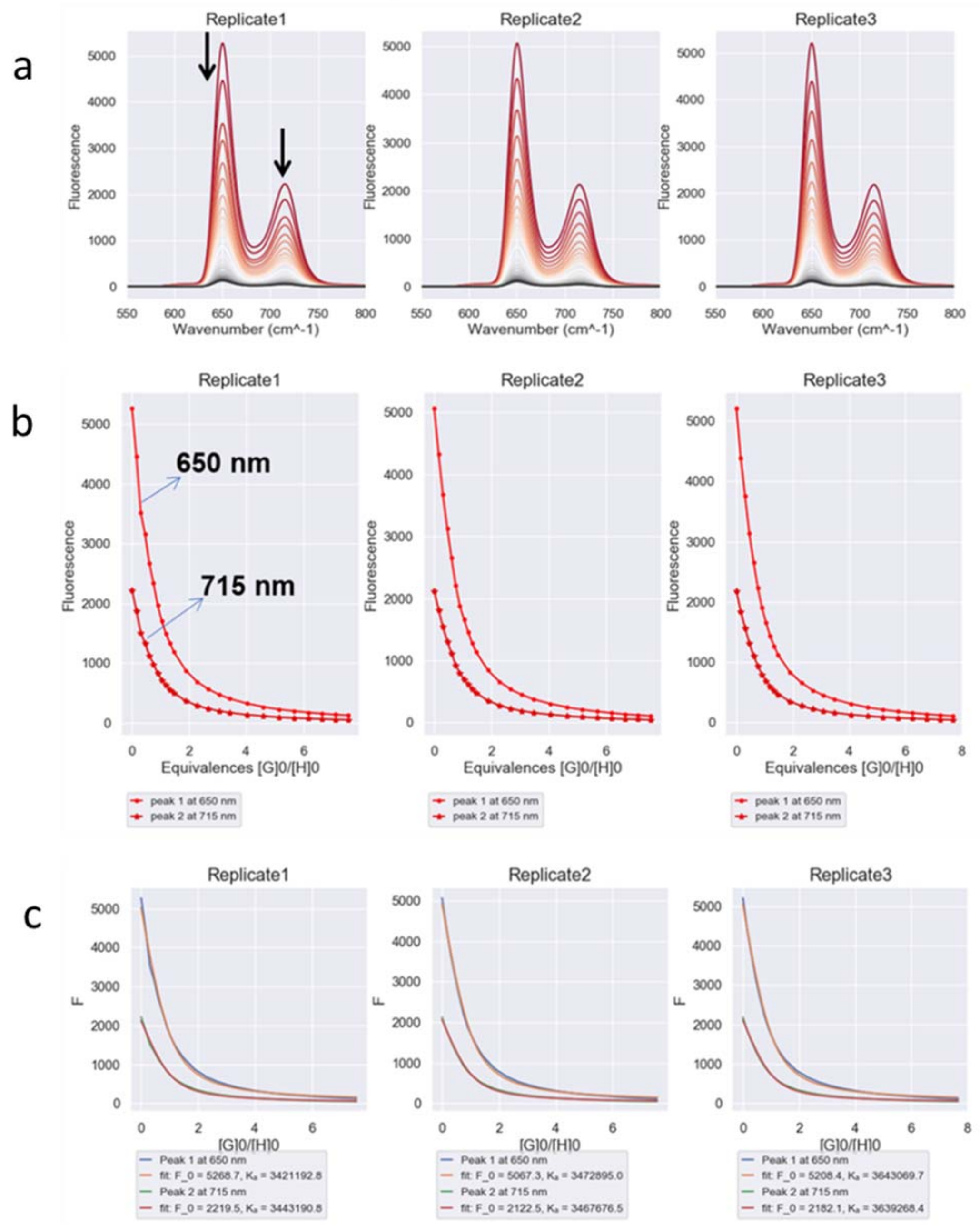

Supplementary Figure 41. Fluorescence titration experiments. a Fluorescence spectra of R_p-(S,R)-**1** upon titration with (R,R)-**6** (triplo). **b** Binding isotherms of R_p-(S,R)-**1** upon titration with (R,R)-**6** at 650 and 715 nm (triplo). **c** Fitting curves (blue line at 650 nm, green line at 715 nm) and binding isotherms (orange line at 650 nm, and red line at 715 nm) (triplo).

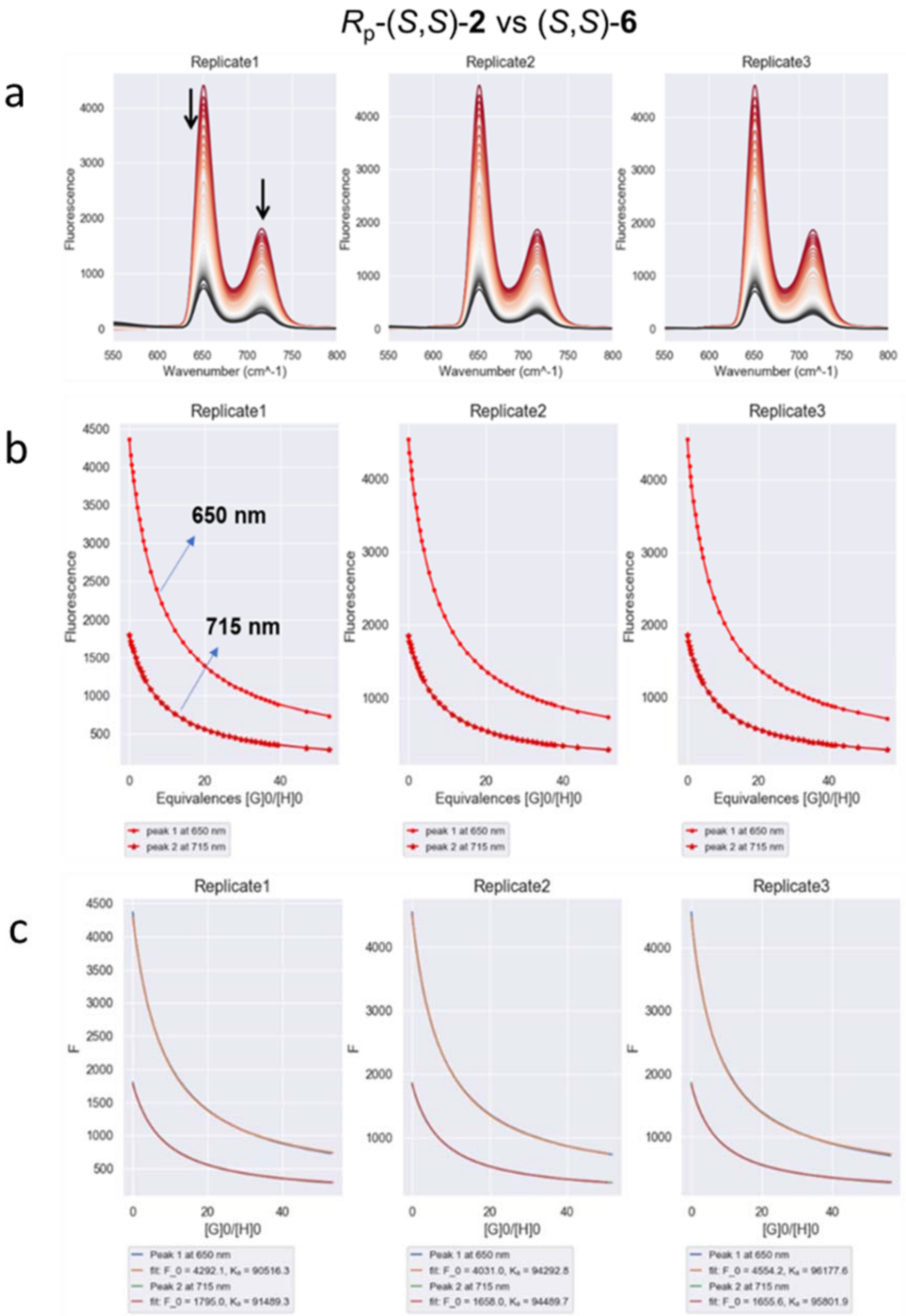

Supplementary Figure 42. Fluorescence titration experiments. a Fluorescence spectra of R_p-(S,S)-**2** upon titration with (S,S)-**6** (triplo). **b** Binding isotherms of R_p-(S,S)-**2** upon titration with (S,S)-**6** at 650 and 715 nm (triplo). **c** Fitting curves (blue line at 650 nm, green line at 715 nm) and binding isotherms (orange line at 650 nm, and red line at 715 nm) (triplo).

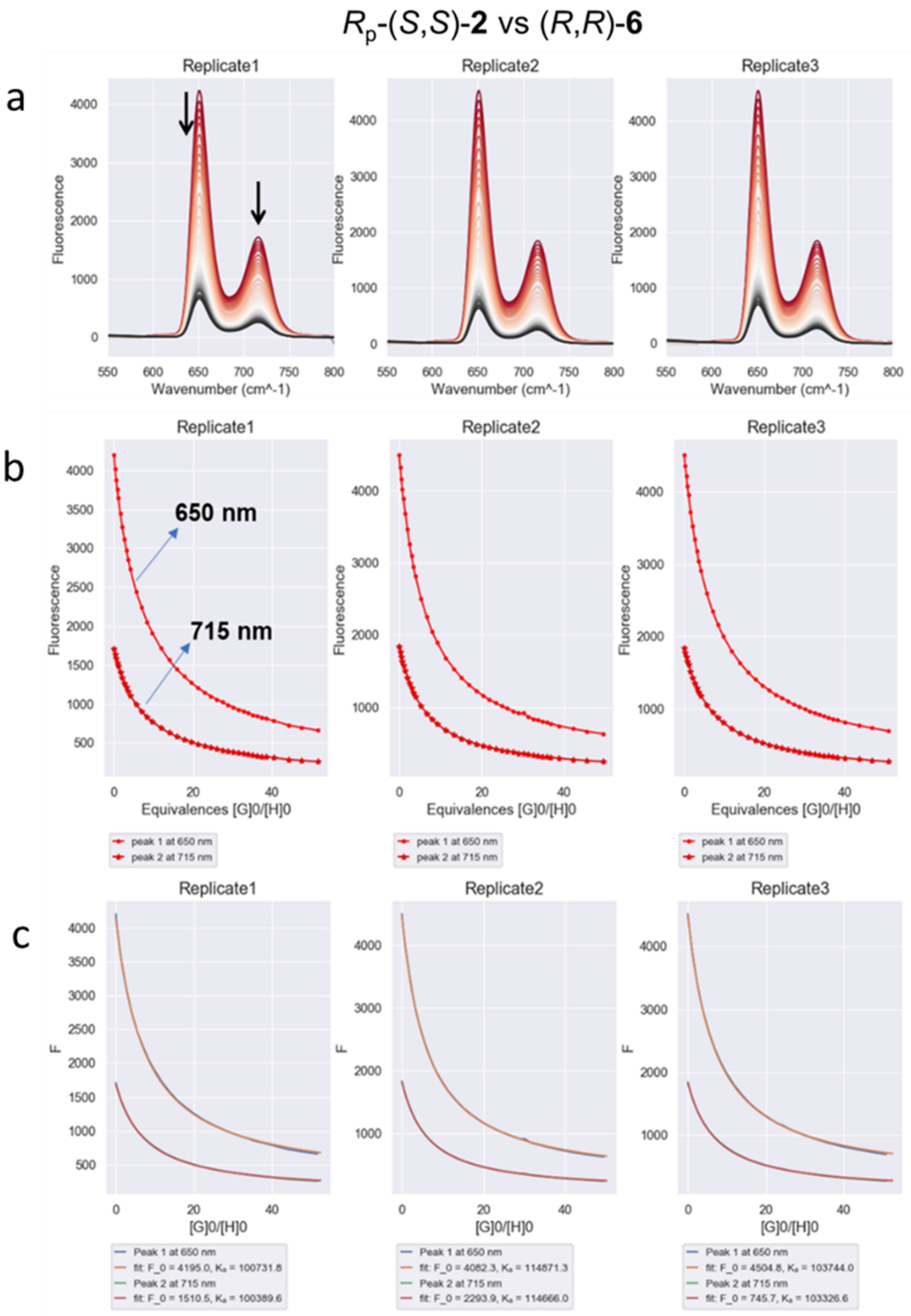

Supplementary Figure 43. Fluorescence titration experiments. a Fluorescence spectra of R_p-(*S*,*S*)-**2** upon titration with (*R*,*R*)-**6** (triplo). **b** Binding isotherms of R_p-(*S*,*S*)-**2** upon titration with (*R*,*R*)-**6** at 650 and 715 nm (triplo). **c** Fitting curves (blue line at 650 nm, green line at 715 nm) and binding isotherms (orange line at 650 nm, and red line at 715 nm) (triplo).

Supplementary Table 2. Association constants of complexes between chiral host and chiral guest compounds in acetonitrile/chloroform 1:1 (v/v) at 298 K.

	(*S*,*S*)-**6**		(*R*,*R*)-**6**	
	K_a at 650 nm ($\times 10^4$ M^{-1})	ΔG^o [$kJmol^{-1}$]	K_a at 650 nm ($\times 10^4$ M^{-1})	K_a at 715 nm ($\times 10^4$ M^{-1})
R_p-(*S*,*R*)-**1**	262 ± 12	−36.6	325 ± 19	323 ± 19
R_p-(*S*,*S*)-**2**	9.4 ± 0.2	−28.3	10.6 ± 0.6	10.6 ± 0.6

IR and VCD measurements

Infrared (IR) spectra and vibrational circular dichroism (VCD) spectra were recorded using two instruments. The first one was a Vertex70 spectrometer to which a PMA50 optical bench was coupled, both being supplied by Bruck company. In the PMA50 set-up the infrared radiation (3800-600 cm^{-1} range) is focused by a BaF_2 lens on a ZnSe photo-elastic modulator (PEM, 50 kHz frequency). The circularly polarized beam is then directed onto the sample and finally collected by a D313/QMTC detector. A calibration of the PEM at a fixed wavenumber had been performed before recording any VCD spectrum, to ensure a proper chiroptical signal within a spectral region of 600 cm^{-1} around the tuning wavenumber. Typically, calibrations at 1450 cm^{-1} allowed us to obtain a spectrum over the most meaningful region for conjugated organic systems. The second instrument was a JASCO FVS-6000 spectrometer, equipped with a MCT-V detector. The spectrometers were allowed to warm up over the course of three hours before a measurement was performed. The detector was cooled with liquid nitrogen before and during the measurement. Samples were measured in a BaF_2 liquid sample cell with a 200 μm optical path length, which was stored in a closed box in the presence of desiccant when not in use. Spectra were acquired with a scan range of 2000-850 cm^{-1}, a resolution of 4 cm^{-1}, and the aperture set at 5.0 mm. A total of 16 accumulations were obtained for the IR spectra and 3000 accumulations for the VCD spectra. Spectra were processed by the spectrometer software by applying zero filling and apodization using a cosine function.

VCD sample preparation

Reference samples for the host were obtained by preparing a 19.5 mM solution of S_p-(*R*,*R*)-**2** (or R_p-(*S*,*S*)-**2**) in $CDCl_3$. Reference samples for the guests were obtained by dissolving (*S*,*S*)-**6** (or (*R*,*R*)-**6)** in CD_3CN with a concentration [**6**] ≈ 40 mM.

The host-guest mixtures were prepared from separately prepared host and guest solutions. Four host solutions were prepared by dissolving 3.0 mg of S_p-(*R*,*R*)-**2** (or R_p-(*S*,*S*)-**2**) in 250 µl of $CDCl_3$. Then, guest solutions were prepared by dissolving (*S*,*S*)-**6** (or (*R*,*R*)-**6)** in CD_3CN with a concentration [**6**] ≈ 40 mM. Then, mixtures were prepared by adding 50 µL of the (*S*,*S*)-**6** (or (*R*,*R*)-**6**) solution (1 equiv.) to the desired S_p-(*R*,*R*)-**2** (or R_p-(*S*,*S*)-**2**) solution. The mixtures were briefly swirled to mix the contents and the solvent was then removed from each mixture by blowing a gentle stream of argon gas over the solution. Subsequently, the mixtures were each redissolved in 250 µl of $CDCl_3$ and the solvent removed again by blowing a gentle stream of argon gas over the solution. This step was repeated one more time. Immediately before measurement, the desired mixture was redissolved in 150 µl of $CDCl_3$ and transferred to the sample cell for measurement ([**2**] ≈ [**6**] ≈ 14 mM). Due to the low signal, for host S_p-(*R*,*S*)-**1** (or R_p-(*S*,*R*)-**1**), the applied concentration was 28 mM.

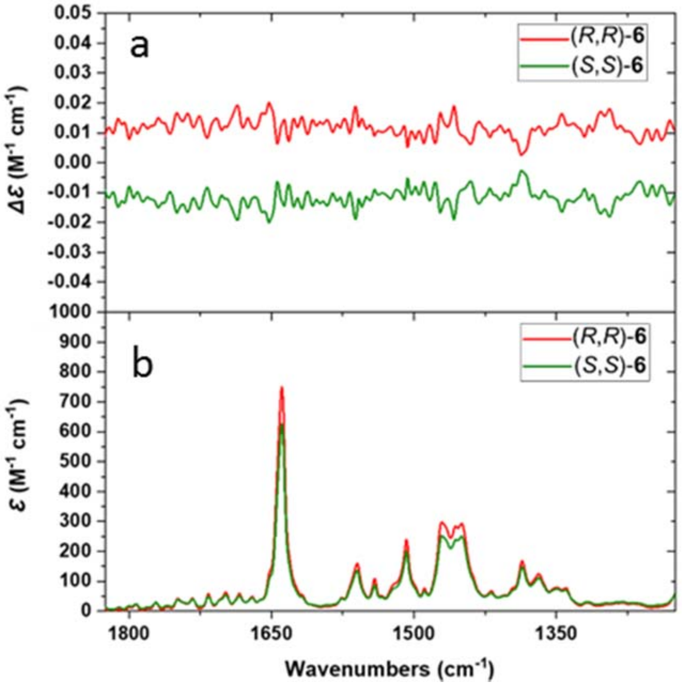

Supplementary Figure 44. VCD and IR spectra. a VCD spectra (1825 cm^{-1} – 1225 cm^{-1}) of (*R*,*R*)-**6** (red line) and (*S*,*S*)-**6** (green line). **b** Corresponding IR spectra. Solvent $CDCl_3$.

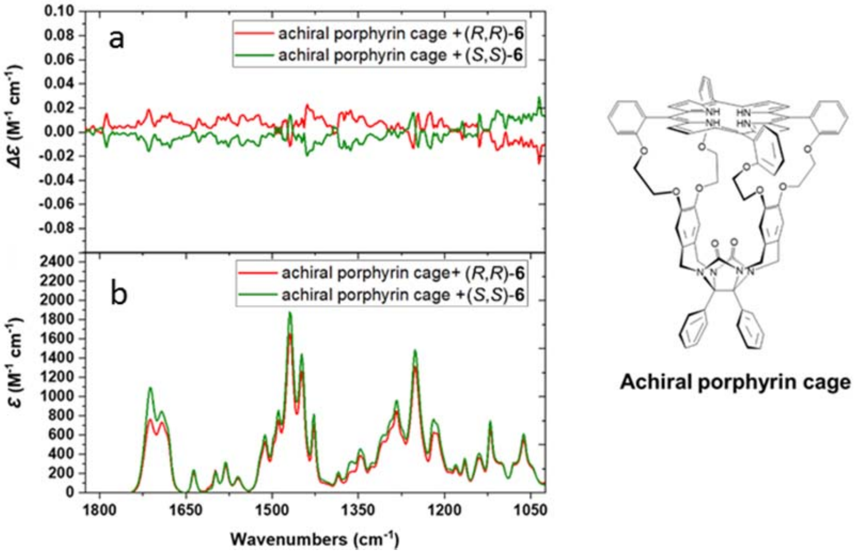

Supplementary Figure 45. VCD and IR spectra. a VCD spectra (1825 cm^{-1} – 1025 cm^{-1}) of (*R*,*R*)-**6** (red line) with the achiral porphyrin cage and (*S*,*S*)-**6** with the achiral porphyrin cage (green line). **b** Corresponding IR spectra. Solvent $CDCl_3$.

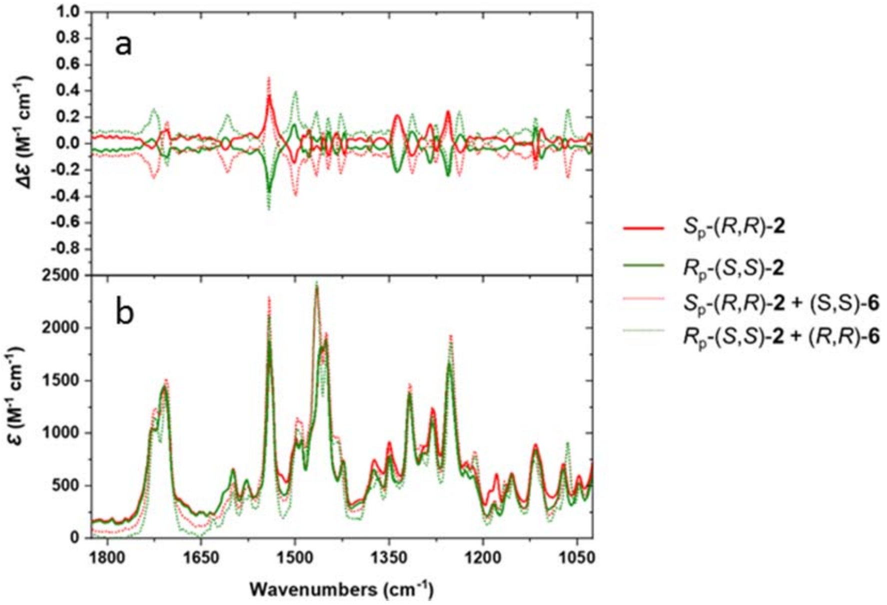

Supplementary Figure 46. VCD and IR spectra. a VCD spectra (1825 cm^{-1} – 1025 cm^{-1}) of S_p-(*R*,*R*)-**2** upon the addition of (*S*,*S*)-**6** and R_p-(*S*,*S*)-**2** upon the addition of (*R*,*R*)-**6**. **b** Corresponding IR spectra. Solvent $CHCl_3$.

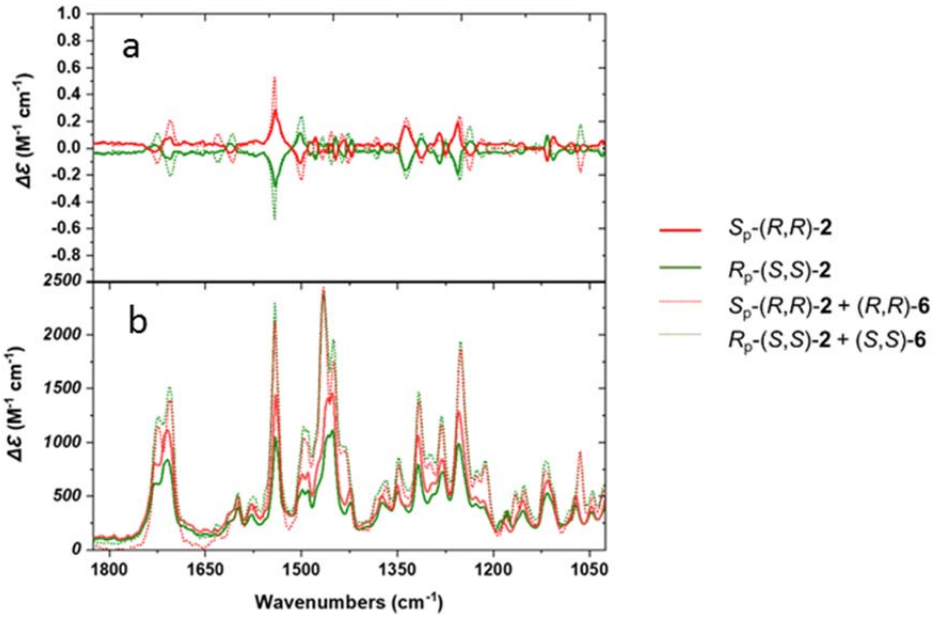

Supplementary Figure 47. VCD and IR spectra. a VCD spectra (1825 cm^{-1} – 1025 cm^{-1}) of S_p-(*R*,*R*)-**2** upon the addition of (*R*,*R*)-**6** and R_p-(*S*,*S*)-**2** upon the addition of (*S*,*S*)-**6**. **b** Corresponding IR spectra. Solvent $CHCl_3$.

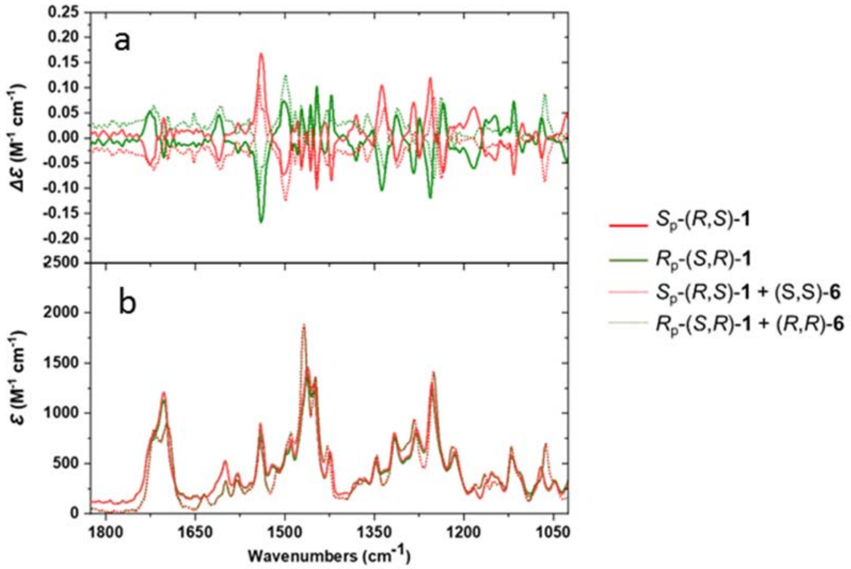

Supplementary Figure 48. VCD and IR spectra. a VCD spectra (1825 cm^{-1} – 1025 cm^{-1}) of S_p-(*R*,*S*)-**1** upon the addition of (*S*,*S*)-**6** and R_p-(*S*,*R*)-**1** upon the addition of (*R*,*R*)-**6**. b Corresponding IR spectra. Solvent $CHCl_3$.

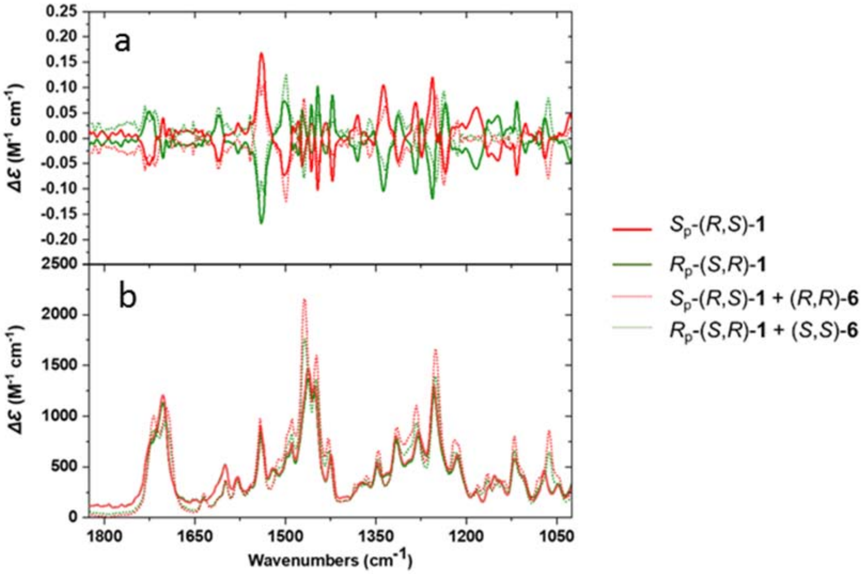

Supplementary Figure 49. VCD and IR spectra. a VCD spectra (1825 cm^{-1} – 1025 cm^{-1}) of S_p-(*R*,*S*)-**1** upon the addition of (*R*,*R*)-**6** and R_p-(*S*,*R*)-**1** upon the addition of (*S*,*S*)-**6**. **b** corresponding IR spectra. Solvent $CHCl_3$.

NMR studies

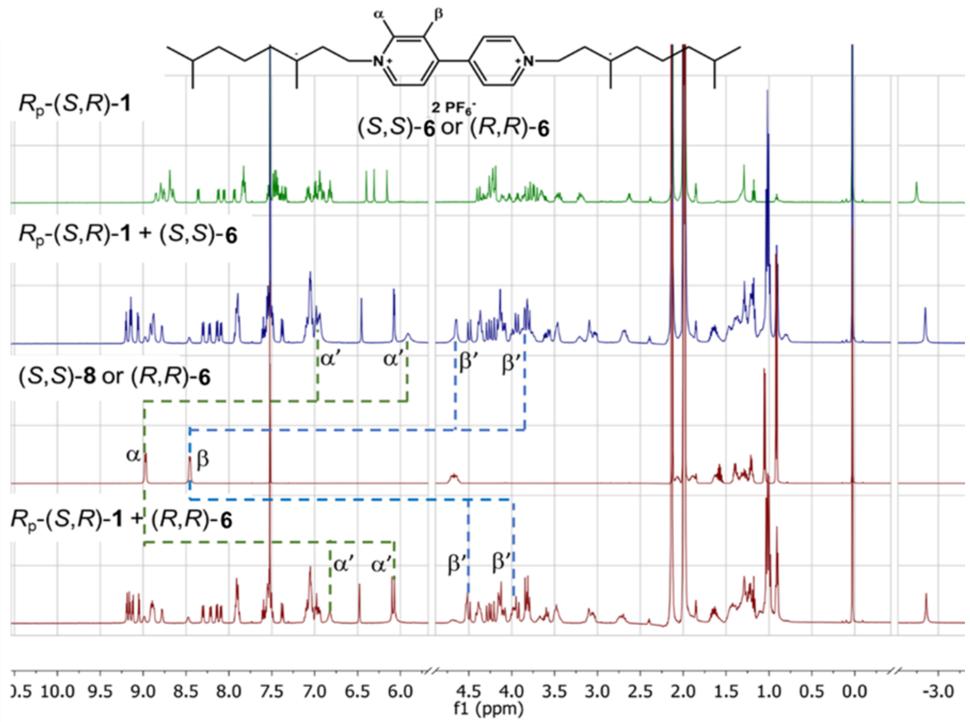

Supplementary Figure 50. ^{1}H NMR spectra (500 MHz, 298 K) of R_p-(*S*,*R*)-**1**, (*S*,*S*)-**6**, complexes R_p-(*S*,*R*)-**1** + (*S*,*S*)-**6** (1:1.02) and R_p-(*S*,*R*)-**1** + (*R*,*R*)-**6** (1:1.02) in mixed solution ($CDCl_3$/CD_3CN, 1:1 v/v) **.**

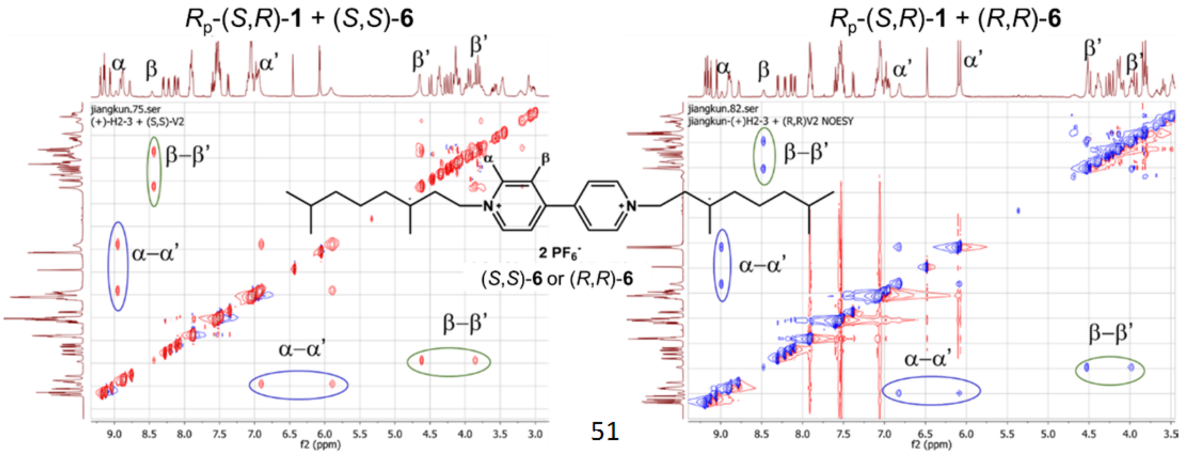

Supplementary Figure 51. 2D NOESY spectra (500 MHz, 298 K) of complexes R_p-(*S*,*R*)-**1** + (*S*,*S*)-**6** (1:1.02) and R_p-(*S*,*R*)-**1** + (*R*,*R*)-**6** (1:1.02) in mixed solution ($CDCl_3/CD_3CN$, 1:1 v/v).

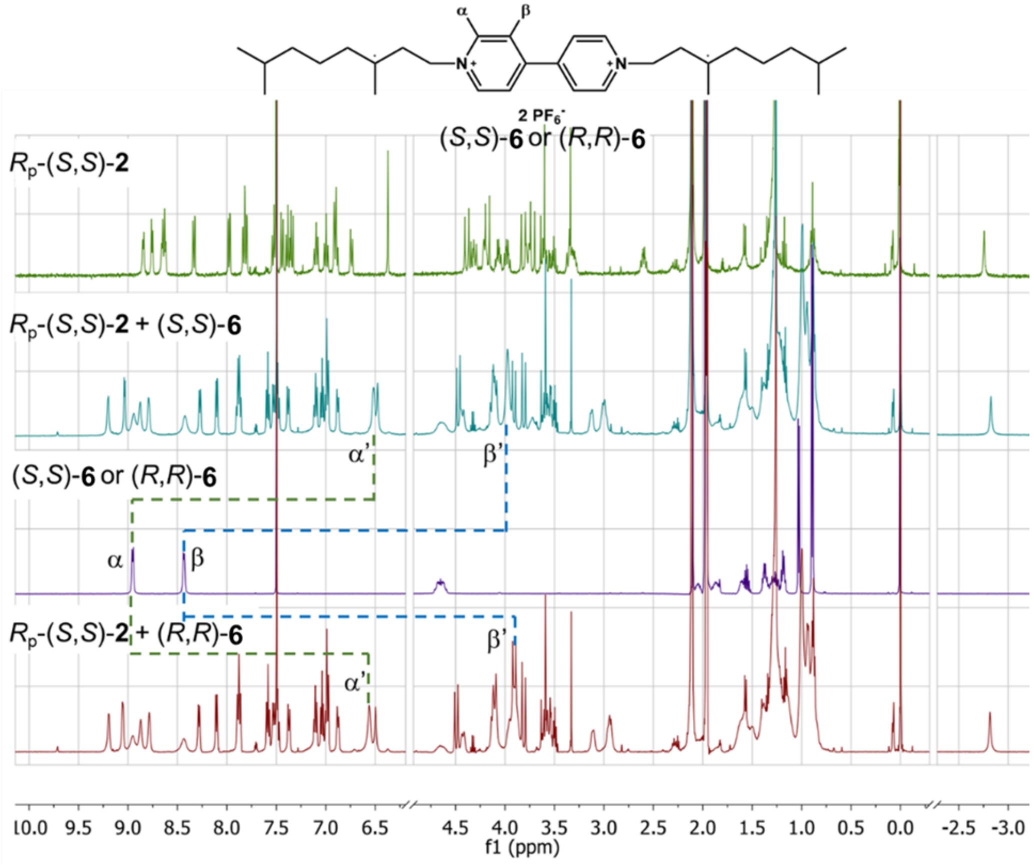

Supplementary Figure 52. ^{1}H NMR spectra (500 MHz, 298 K) of R_p-(*S*,*S*)-**2**, (*S*,*S*)-**6**, complexes R_p-(*S*,*S*)-**2** + (*S*,*S*)-**6** (1:1.02) and R_p-(*S*,*S*)-**2** + (*R*,*R*)-**6** (1:1.02) in mixed solution ($CDCl_3/CD_3CN$, 1:1 v/v).

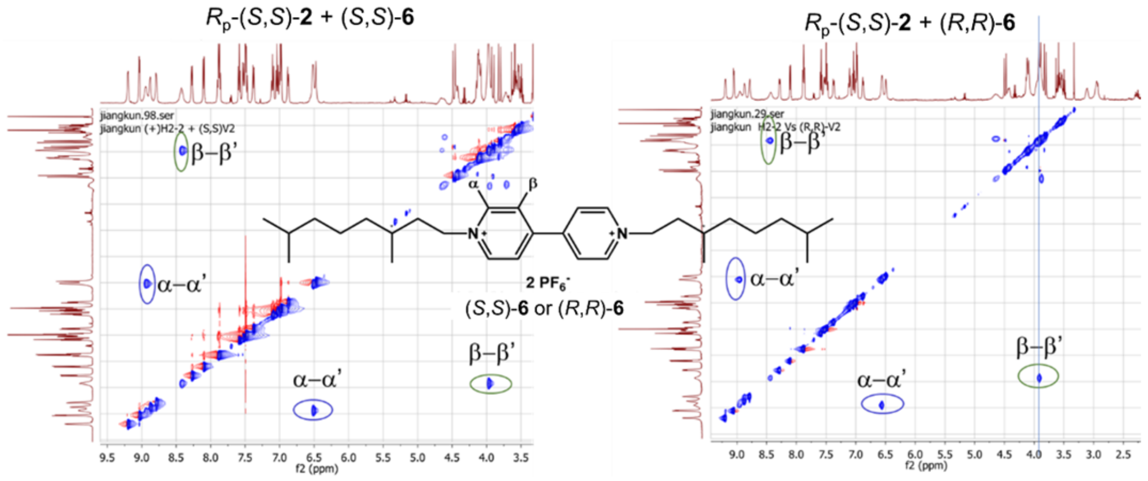

Supplementary Figure 53. 2D NOESY spectra (500 MHz, 298 K) of complexes R_p-(*S*,*S*)-**2** + (*S*,*S*)-**6** (1:1.02) and R_p-(*S*,*S*)-**2** + (*R*,*R*)-**6** (1:1.02) in mixed solution ($CDCl_3$/CD_3CN, 1:1 v/v)**.**

Computations

Calculations of the IR, VCD, UV, and ECD spectra

Firstly, spectra of isolated molecules without explicit solvent molecules were calculated for the *S*p-(*R*,*R*)-**2** enantiomer. Only the average effects of the solvent were taken into account using the implicit solvation model SMD ("Solvation Model based on Density").[9] Each geometry was optimized using Density Functional Theory with the B3LYP functional and triple zeta 6-311G(d) basis set. Empirical dispersion was added with the D3 version of Grimme's dispersion with Becke-Johnson damping (GD3BJ).[10] The vibrational frequencies, IR absorption, and VCD intensities were calculated using the same level of theory. Frequencies were scaled by a factor of

0.975. IR absorption and VCD spectra were constructed from calculated dipole and rotational strengths assuming Lorentzian band shape with a half-width at half maximum of 8 cm^{-1}. All calculations were performed using the Gaussian16 package.[11] The conformations selected for the calculations of the averaged IR/VCD spectra of *S*p-(*R*,*R*)-**2** were obtained by molecular dynamics calculations, see below. Eight conformations were selected and optimized. Boltzmann populations estimated from enthalpies calculated at 298K revealed that the 5 most stable conformations among the 8 found are required for the calculation of the averaged spectra (Supplementary Table 3, Supplementary Figure 54).

Supplementary Table 3. Enthalpies and Boltzmann population of conformations A_{1-8} of diastereomers of *Sp*-(*R*,*R*)-**2** calculated using SMD(dichloromethane)/GD3BJ-B3LYP/6-311G(d) level

Conformations	H^{298K} (in a.u)	ΔH^{298K} (in kcal.mol^{-1})	Boltzmann Distribution
**A_1**	-4838.955018	0.000	**0.35**
**A_2**	-4838.954889	0.081	**0.30**
**A_3**	-4838.953848	0.734	**0.10**
**A_4**	-4838.953819	0.752	**0.10**
**A_5**	-4838.953511	0.946	**0.07**
A_6	-4838.953059	1.229	0.04
A_7	-4838.952896	1.332	0.04
A_8	-4838.950171	3.042	0.00

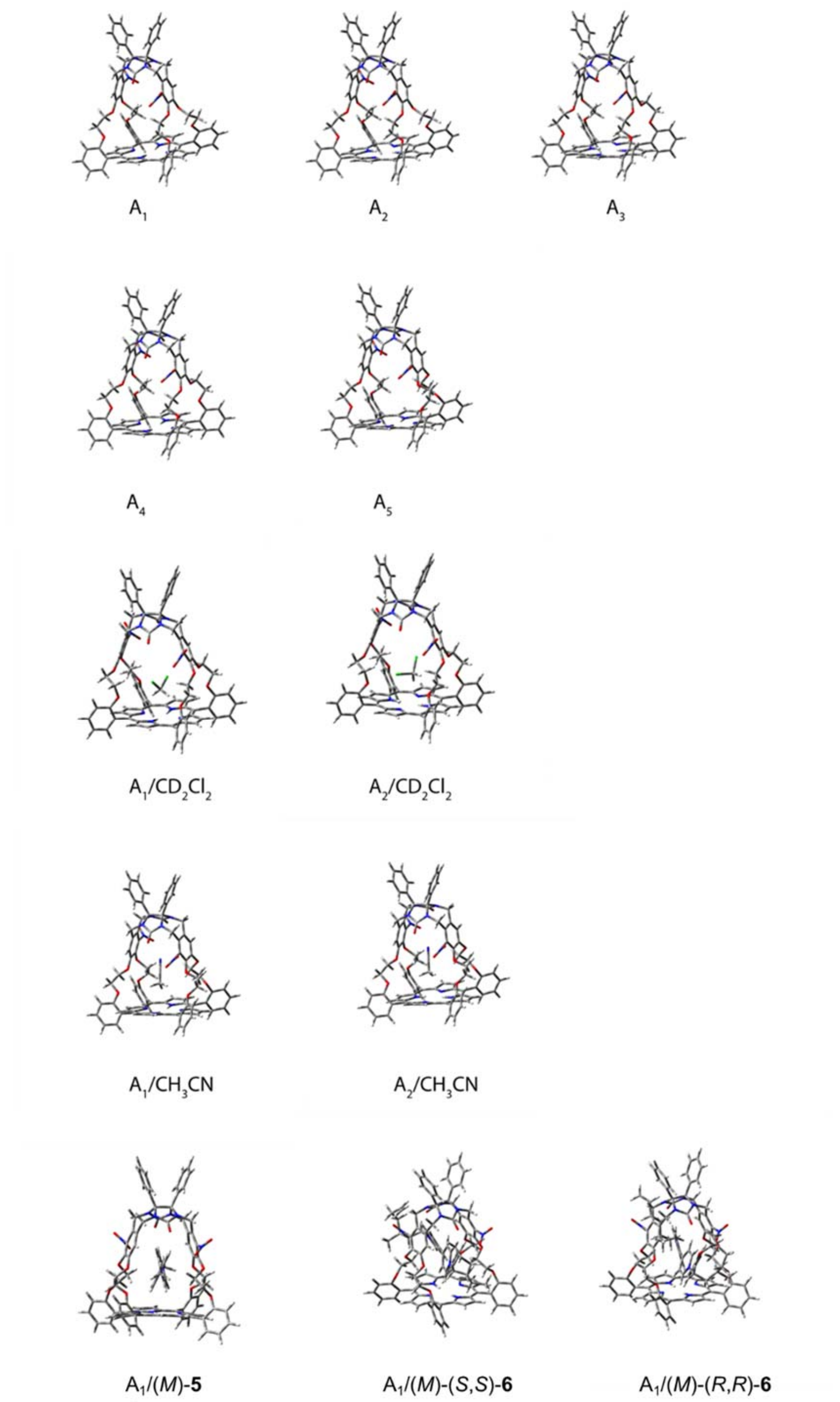

Supplementary Figure 54. DFT Calculated structures.

In this way, an acceptable but not satisfying agreement was obtained between the spectra measured for the enantiomer (+)-**2** and calculated for the enantiomer *S*p-(*R*,*R*)-**2** (Supplementary Figure 55).

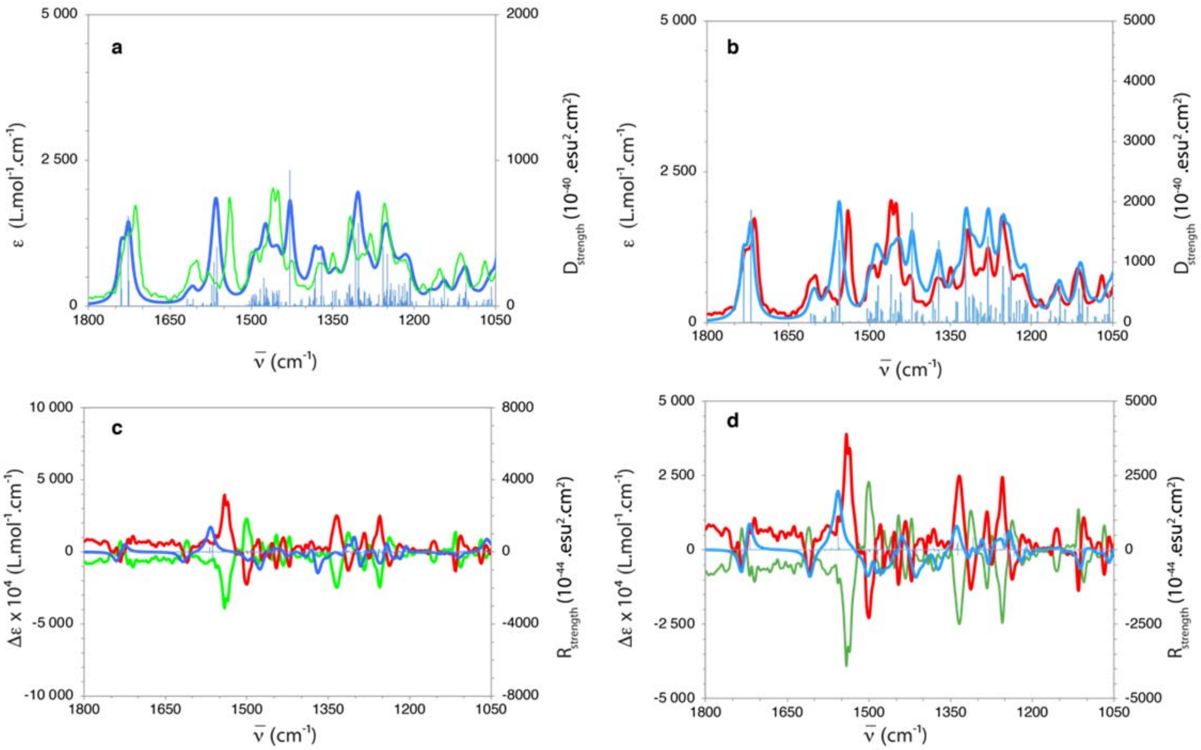

Supplementary Figure 55. Experimental and calculated IR and VCD spectra. a IR spectra measured for (-)-**1** (green line) and calculated for S_p-(*R*,*R*)-**2** (blue line and blue bars: dipole strengths). **b** IR spectra measured for (+)-**2** (red line) and calculated for S_p-(*R*,*R*)-**2**/CD_2Cl_2 (blue and blue bars: dipole strengths). **c** VCD spectra measured for (–)-**1** (green line), (+)-**1** (red line), and calculated for S_p-(*R*,*R*)-**2** (blue line and blue bars: rotational strengths). **d** VCD spectra measured for (–)-**2** (green line), (+)-**2** (red line), and calculated for S_p-(*R*,*R*)-**2**/CD_2Cl_2 (blue line and blue bars: rotational strengths). Solvent CD_2Cl_2, c = 1 × 10^{-1} M.

Calculations performed by introducing an explicit solvent molecule inside the two most stable conformations found for *S*p-(*R*,*R*)-**2** made the agreement between measurement and calculation significantly more satisfactory. The position of the explicit solvent molecule (CD_2Cl_2) in the

cage of porphyrin *S*p-(*R*,*R*)-**2** was initially determined using molecular dynamics calculations and thereafter the complex was optimized using the SMDCD$_2$Cl$_2$/GD3BJ-B3LYP/6-311G(d) DFT level. Given the quality of this result with only two conformations and considering the large size of the studied system for which a significant amount of calculation time is required, this model was not extended to other conformations. The results obtained for compound **2** were also used for the calculations of the spectra of the *S*p-(*R*,*S*)-**1** enantiomer, as well as for the calculations of the UV and ECD spectra of both compounds **1** and **2**. The geometries used for the *S*p-(*R*,*S*)-**1** compound were optimized from the geometries of the retained conformations of *S*p-(*R*,*R*)-**2** in which one of the two nitro groups was replaced by an hydrogen atom. As with **2**, a good balance between accuracy of the results and the consumed cpu time was obtained by considering only two conformations with one explicit solvent molecule using the SMD_{CD2Cl2}/GD3BJ-B3LYP/6-311G(d) theoretical level. For the calculations of the UV and ECD spectra a similar approach was applied but with an explicit CH_3CN molecule instead of CD_2Cl_2. The geometry optimizations were performed using WB97XD functional associated with the 6-31G(d) basis set and the SMD implicit solvent model. Based on these geometries, the ECD and UV spectra were calculated using the time dependent density functional theory (TD-DFT) with LC-WhPBE functional and def2SVP basis set. Calculations were performed for vertical 1A singlet excitation using 100 states. For a comparison between theoretical results and the experimental values, the calculated UV and ECD spectra were modeled with a Gaussian function using a half-width of 0.37 eV. Due to the approximations of the used theoretical model, an almost constant offset was observed between measured and calculated wavelengths. Using UV spectra, all calculated wavelengths were calibrated by a factor of 1.05.

Molecular dynamics calculations

Molecular dynamics studies were used to find the stable conformations of the *S*p-(*R*,*R*)-**2** enantiomer and to determine where to put the explicit solvent molecules within the host. The starting geometries that were used for the calculations of the spectra at DFT level were obtained by performing molecular dynamics simulations using the GAFF force field implemented in Gromacs 5. This computational method provides detailed information on the fluctuations and the conformational changes of the porphyrin molecule. The solute-solvent superstructures of the

porphyrin with one dichloromethane or acetonitrile molecule were considered as the starting geometries for further DFT calculations.

Parametrization of the porphyrin for molecular dynamics simulations

The Generalized Amber Force Field (GAFF) was used to model the porphyrin molecule. The Antechamber and Tleap modules in the AMBERTools18 suite of programs was employed to generate the initial parameter topology files. The restrained electrostatic potential (RESP) charge was determined from quantum chemical calculation at the HF/6-31G (d) level using the Gaussian16 package. Prior to calculating RESP charge, we optimized the geometry of the porphyrin molecule at the B3LYP/6-31G (d) level. Except for RESP calculations, all the quantum chemical calculations were performed using the "Solvation Model based on Density" (SMD) implicit solvation model, in consideration of the solvation effect of the dichloromethane or acetonitrile.

Molecular Dynamic simulations*:*

Molecular dynamics (MD) simulations were performed starting from the atomistic coordinates of the optimized structure obtained from quantum chemical calculation. The porphyrin molecule was inserted in a cubic box whose sides measured 15 Å and solvated with around 1000 molecules of dichloromethane or acetonitrile. We initially minimized the energy and then performed equilibrium MD under periodic boundary conditions in NPT ensemble. MD trajectory was followed for 100ns in NVT ensemble. The temperature during the simulation was held constant at 300K using Berendsen thermostat. Fast smooth Particle-Mesh Ewald summation was used for long-range electrostatic interactions, with a cutoff of 1.0 nm for the direct interactions.

Search of conformations using the cluster method of Gromacs

We used the clustering analysis tool of GROMACS (gmx cluster) to explore the different conformations from the MD trajectory. The GROMOS clustering algorithm with a RMSD cut-off was used to determine the structurally similar clusters. This approach allowed us to select 8

geometries that were optimized and used for the calculations of the average IR and VCD spectra (Supplementary Figure 55). The same approach was used to the selection of geometries of the cage porphyrin with a solvent molecule inside (Supplementary Figure 54).

VCD bands amplification and Induced CD phenomenon

The geometries the host *S*p-(*R*,*R*)-**2** and guests **5**, (*S*,*S*)-**6**, and (*R*,*R*)-**6** were optimized using the WB97XD functional software associated with the 6-311G(d) basis set but without implicit solvent effects in order to keep a reasonable use of cpu time. In order to better understand the Induced CD phenomenon, we optimized one conformation of the complexes formed by S_p-(*R,R*)-**2** and the two enantiomorphic conformations of viologen **5**. Two possibilities were considered: viologen **5** is hosted in a 'horizontal' orientation (perpendicular to the xylylene sidewalls and parallel to the porphyrin ring) or in a 'vertical' position orientation (parallel to the xylylene side walls and vertical to the porphyrin ring) in the cavity of the host. Attempts to optimize the complex S_p-(*R*,*R*)-**2**/(*M*)-**5** starting from geometries in which the guest is in a 'horizontal' position all converged towards a conformation in which **5** had rotated to a 'vertical' position. Similarly, all attempts to optimize the S_p-(*R*,*R*)-**2**/(*P*)-**5** complex converged to the S_p-(*R*,*R*)-**2**/(*M*)-**5** complex. Based on the optimized geometries of (*M*)-**5**, S_p-(*R*,*R*)-**2**, and the complex S_p-(*R*,*R*)-**2**/(*M*)-**5**, the corresponding ECD and UV spectra were calculated using time dependent density functional theory (TD-DFT) with the LC-WhPBE functional and def2SVP basis set. Calculations were performed for vertical 1A singlet excitation using 50 states. In order to compare the theoretical results with the experimental values, the calculated UV and ECD spectra were modeled with a Gaussian function using a half-width of 0.37 eV. Due to the approximations of the used theoretical model an almost constant offset was observed between the measured and calculated wavelengths. Using UV spectra, all calculated wavelengths were calibrated by a factor of 1.05.

For the S_p-(*R*,*R*)-**2**/(*M,S,S*)-**6** and S_p-(*R*,*R*)-**2**/(*M,R,R*)-**6** complexes, we looked for the most stable conformations adopted by the side chains of the viologens complexed in the cavity. We used a methodology combining DFT calculations and simulated annealing performed at the semi-empirical level. From an initial geometry of the complex optimized using the WB97XD/6-

311G(d) DFT level, we carried out a simulated annealing at the AM1 semi-empirical level, allowing the side chains of the viologen to adopt different conformations on the dihedral angles only. The bond lengths, valence angles, and dihedral angles not involved in the conformational mobility of the viologen side chains were fixed in these simulated annealing calculations. The lower energy conformation found by this approach was then fully optimized using the WB97XD/6-311G(d) level (Supplementary Figure 54) before calculating the IR and VCD spectra (Supplementary Figure 56). The AMPAC10 program was used for all the semi-empirical simulated annealing calculations.[12]

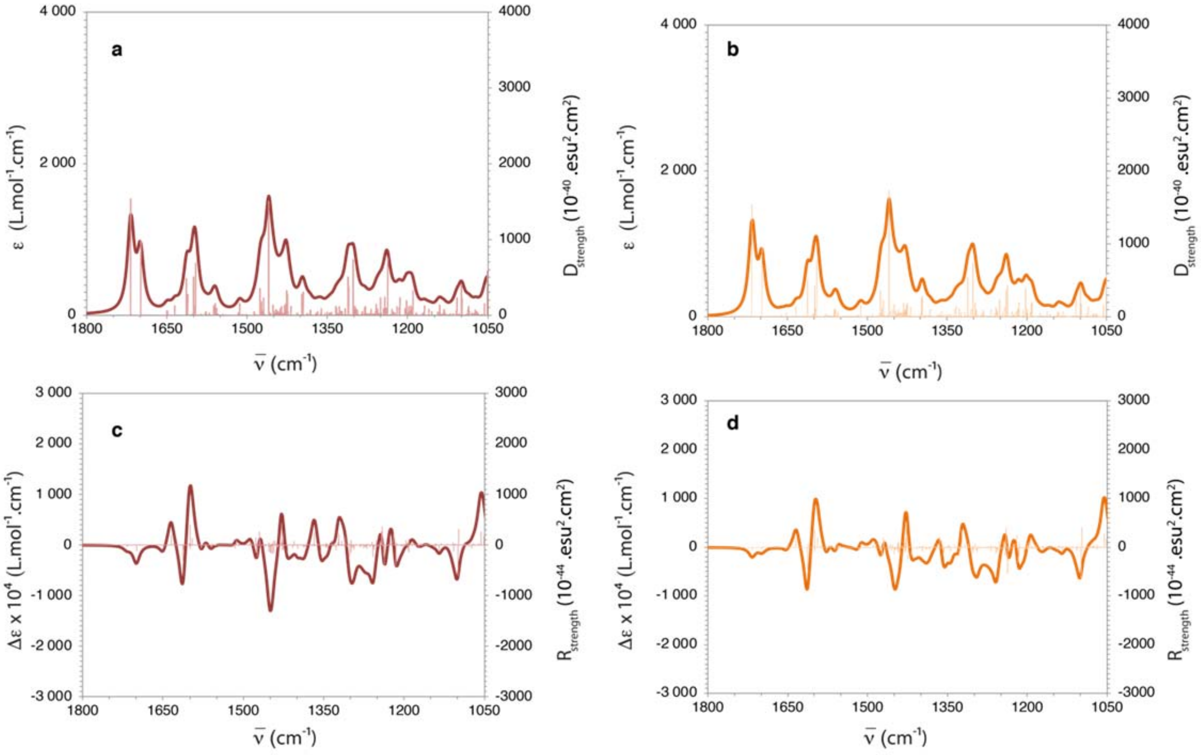

Supplementary Figure 56. Calculated IR and VCD spectra. a IR spectra calculated for S_p-(*R*,*R*)-**2** /(*M*,*S*,*S*)-**6** (brown line and brown bars: dipole strengths). **b** IR spectra calculated for S_p-(*R*,*R*)-**2** /(*M*,*R*,*R*)-**6** (orange and orange bars: dipole strengths). **c** VCD spectra calculated for S_p-(*R*,*R*)-**2** /(*M*,*S*,*S*)-**6** (brown line and brown bars: rotational strengths). **d** VCD spectra calculated for S_p-(*R*,*R*)-**2** /(*M*,*R*,*R*)-**6** (orange and orange bars: rotational strengths).

X-ray structures

Single crystals of **(-)-*R*$_p$-(*S*,*R*)-1** and **(+)-*S*$_p$-(*R*,*S*)-3** were both grown from a mixture of $CHCl_3$ and CD_3CN (1:1 v/v).

Reflections were measured on a Bruker D8 Quest diffractometer with sealed tube and Triumph monochromator (λ = 0.71073Å). Software package used for the intensity integration was Saint.[13] Absorption correction was performed with SADABS.[14] The structures were solved with direct methods using SHELXT.[15] Least-squares refinement was performed with SHELXL-2014[16] against $|F_h^o|^2$ of all reflections. Non-hydrogen atoms were refined freely with anisotropic displacement parameters. Hydrogen atoms were placed on calculated positions or located in difference Fourier maps. All calculated hydrogen atoms were refined with a riding model.

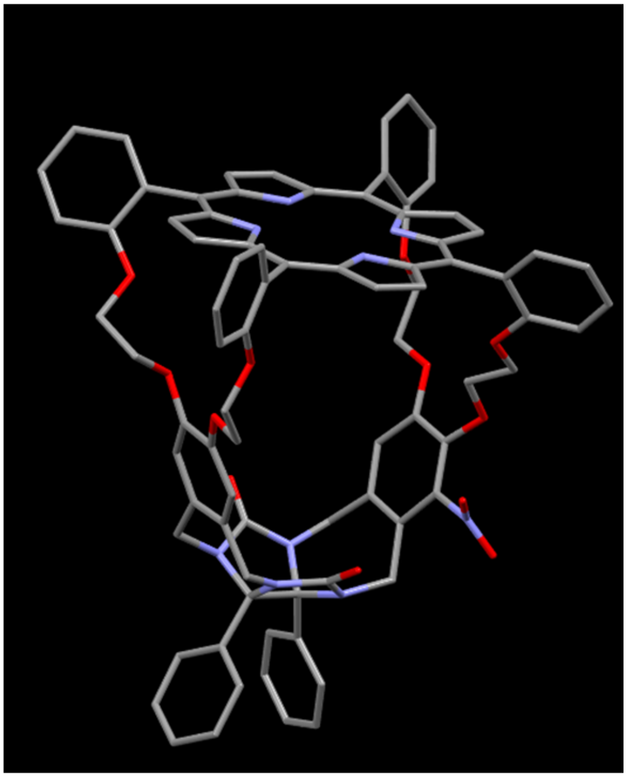

Supplementary Figure 57 Solid state structure of **(-)-**R_p-(S,R)-**1.** The cif-file was deposited in the Cambridge Structural Database under identifier CCDC 1989977.

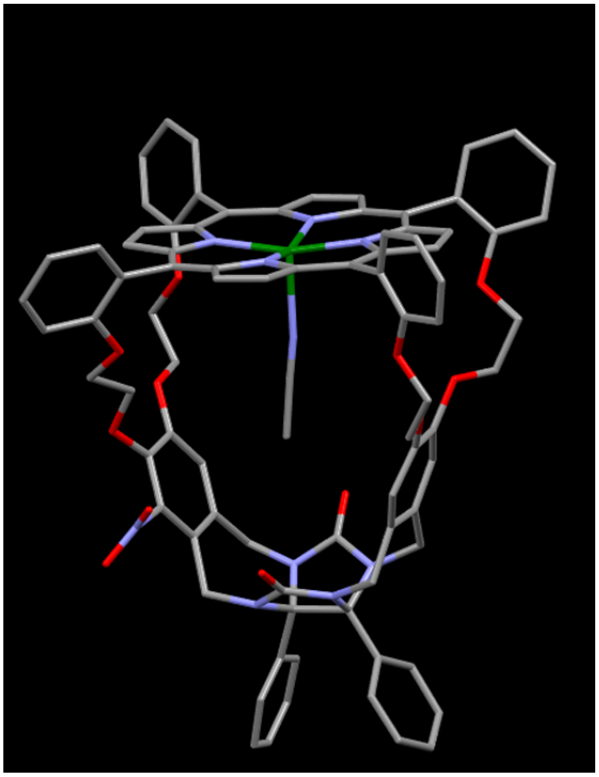

Supplementary Figure 58. Solid state structure of **(+)-**S_p-(R,S)-**3**. The cif-file was deposited in the Cambridge Structural Database under identifier CCDC 1989978.

Supplementary Table 4 Crystal data

**Crystal structure and structure refinement**		
**General information**	R_p-(*S*,*R*)-**1**	S_p-(*R*,*S*)-**3***
Identification code RU	p2021a	p1943a
Crystal colour	blue	blue
Crystal dimensions [mm] / shape	0.04 x 0.18 x 0.57/ needle	0.05 x 0.07 x 0.40 / needle
Crystallization solvent	Chloroform and acetonitrile (v/v 1:1)	Chloroform and acetonitrile (v/v 1:1)
Formula moiety	$C_{84}H_{63}N_9O_{12}$, 2.305($CHCl_3$), C_2H_3N	$C_{86}H_{60}N_{10}O_{12}Zn$, C_2H_3N + solvent
Formula weight [g/mol]	1706.42	1531.86
**Crystal Data**		
Crystal system	Orthorhombic	Orthorhombic
Space group	$P2_12_12_1$ (No. 19)	$P2_12_12_1$ (No. 19)
Unit cell dimensions a, b, c [Å] α, β, γ [°]	12.5790(4), 22.9476(8), 28.5212(9) 90, 90, 90	12.8091(8), 24.3430(17), 25.708(2) 90, 90, 90
Volume [$Å^3$]	1396.9(4)	8016.1(10)
Z	4	4
Density (calculated) [g/cm^3]	1.377	1.269
Absorption coefficient (MoKα) [mm^{-1}]	0.308	0.375
F(000)	3527	3176
**Data Collection**		
Temperature during experiment [K]	150	150
Wavelength [Å]	0.71073	0.71073
θ Min-Max [°]	2.0, 28.3	2.4, 22.0
Index range	$-16 \leq h \leq 16$; $-30 \leq k \leq 30$; $-38 \leq l \leq 38$	$-13 \leq h \leq 13$; $-25 \leq k \leq 25$; $-27 \leq l \leq 20$
Tot., Uniq. Data, R(int)	159791, 20449, 0.065	19788, 9462, 0.097
Observed Data [I > 2.0 σ(I)]	17027	5531
**Refinement**		
Nref, Npar	20449, 1085	9462, 1011
R, wR2, S	0.0622, 0.1764, 1.03	0.0761, 0.1936, 1.02
Min. and Max. Resd. Dens. [e/ $Å^3$]	-0.52, 1.36	-0.56, 0.67

**Bijvoet analysis**		
Number of Bijvoet Pairs	9337 (100%)	4104 (93%)
Flack x	0.032(14)	0.010(15)
Parsons z	0.035(14)	0.091(11)
P2(true)	1.000	1.000
P3(true)	1.000	1.000
P3(rac-twin)<br>G	0.0E+00<br>0.96(3)	0.0E+00<br>0.98(2)
Hooft y	0.020(13)	0.012(11)

*It was not possible to obtain high quality crystals of S_p-(*R,S*)-**3** for the structure determination. Despite the limited resolution of the crystal structure we do believe that its chirality is properly assigned.

Supplementary References

1. Testa, B., *Helvetica Chimica Acta* **2013**, *96*, 351-374.
2. Hirschmann, H.; Hanson, K. R., *J. Org. Chem.* **1971**, *36*, 3293-3306.
3. Cahn, R. S.; Ingold, C.; Prelog, V., *Angew. Chem. Int. Ed.* **1966**, *5*, 385-415.
4. Prelog, V.; Helmchen, G., *Angew. Chem. Int. Ed.* **1982**, *21*, 567-583.
5. Lemiere, G. L.; Alderweireldt, F. C., *J. Org. Chem.* **1980**, *45*, 4175-4179.
6. Elemans, J. A. A. W.; Claase, M. B.; Aarts, P. P. M.; Rowan, A. E.; Schenning, A. P. H. J.; Nolte, R. J. M., *J. Org. Chem.* **1999**, *64*, 7009-7016.
7. Masci, B.; Pasquale, S.; Thuéry, P., *Org. Lett.* **2008**, *10*, 4835-4838.
8. Nikitin, K.; Müller-Bunz, H., *New. J. Chem.*, **2009**, *33*, 2472-2478.
9. Marenich, A. V. ; Cramer, C. J. ; Truhlar, D. G. *J. Phys. Chem. B* **2009**, 113, 6378-6396. 10. Grimme, S. ; Ehrlich, S.; Goerigk, L. *J. Comp. Chem.* **2011**, *32*, 1456-1465.
11. Gaussian 16, Revision A.03, M. J. Frisch, G. W. Trucks, H. B. Schlegel, G. E. Scuseria, M. A. Robb, J. R. Cheeseman, G. Scalmani, V. Barone, G. A. Petersson, H. Nakatsuji, X. Li, M. Caricato, A. V. Marenich, J. Bloino, B. G. Janesko, R. Gomperts, B. Mennucci, H. P. Hratchian, J. V. Ortiz, A. F. Izmaylov, J. L. Sonnenberg, D. Williams-Young, F. Ding, F. Lipparini, F. Egidi, J. Goings, B. Peng, A. Petrone, T. Henderson, D. Ranasinghe, V. G. Zakrzewski, J. Gao, N. Rega, G. Zheng, W. Liang, M. Hada, M. Ehara, K. Toyota, R. Fukuda, J. Hasegawa, M. Ishida, T. Nakajima, Y. Honda, O. Kitao, H. Nakai, T. Vreven, K. Throssell, J. A. Montgomery, Jr., J. E. Peralta, F. Ogliaro, M. J. Bearpark, J. J. Heyd, E. N. Brothers, K. N. Kudin, V. N. Staroverov, T. A. Keith, R. Kobayashi, J. Normand, K. Raghavachari, A. P. Rendell, J. C. Burant, S. S. Iyengar, J. Tomasi, M. Cossi, J. M. Millam, M. Klene, C. Adamo, R. Cammi, J. W. Ochterski, R. L. Martin, K. Morokuma, O. Farkas, J. B. Foresman, and D. J. Fox, Gaussian, Inc., Wallingford CT, 2016.

12. AMPAC 10, 1992-2013 Semichem, Inc. 12456 W 62nd Terrace - Suite D, Shawnee, KS 66216.
13. SAINT V8.38A. Bruker AXS Inc., Madison, Wisconsin, USA.
14. SADABS-2016/2, Krause, L.; Herbst-Irmer, R.; Sheldrick, G. M., and Stalke, D. *J. Appl. Crystallogr.* **2015**, 48, 3-10.
15. Sheldrick, G. M. *Acta Cryst.* **2015**, A71, 3-8.
16. Sheldrick, G. M. *Acta Cryst.* **2015**, C71, 3-8.
